# Phylogenetic history influences convergence for a specialized ecology: comparative skull morphology of African burrowing skinks (Squamata; Scincidae)

**DOI:** 10.1186/s12862-021-01821-w

**Published:** 2021-05-16

**Authors:** Natasha Stepanova, Aaron M. Bauer

**Affiliations:** 1 Department of Biology and Center for Biodiversity and Ecosystem Stewardship, Villanova University, Villanova, PA USA; 2 Present Address: Department of Ecology and Evolutionary Biology, University of Michigan, Ann Arbor, MI USA

**Keywords:** Squamates, Computed tomography, Macroevolution, Anatomy, Convergent evolution, Fossorial, Cranium

## Abstract

**Background:**

Skulls serve many functions and as a result, are subject to many different evolutionary pressures. In squamates, many fossorial species occupy a unique region of skull morphospace, showing convergence across families, due to modifications related to head-first burrowing. As different substrates have variable physical properties, particular skull shapes may offer selective advantages in certain substrates. Despite this, studies of variation within burrowers have been limited and are typically focused on a single origin of fossoriality. We focused on seven skink genera (*Acontias*, *Typhlosaurus*, *Scelotes*, *Sepsina*, *Feylinia*, *Typhlacontias*, and *Mochlus*; 39 sp.) from southern Africa, encompassing at least three independent evolutions of semi-fossoriality/fossoriality. We used microCT scans and geometric morphometrics to test how cranial and mandibular shape were influenced by phylogenetic history, size, and ecology. We also qualitatively described the skulls of four species to look at variation across phylogenetic and functional levels, and assess the degree of convergence.

**Results:**

We found a strong effect of phylogenetic history on cranial and mandibular shape, with size and substrate playing secondary roles. There was a clear gradient in morphospace from less specialized to more specialized burrowers and burrowers in sand were significantly different from those in other substrates. We also created an anatomical atlas for four species with each element described in isolation. Every bone showed some variation in shape and relative scaling of features, with the skull roofing bones, septomaxilla, vomer, and palatine showing the most variation. We showed how broad-scale convergence in traits related to fossoriality can be the result of different anatomical changes.

**Conclusions:**

Our study used geometric morphometrics and comparative anatomy to examine how skull morphology changes for a highly specialized and demanding lifestyle. Although there was broad convergence in both shape and qualitative traits, phylogenetic history played a large role and much of this convergence was produced by different anatomical changes, implying different developmental pathways or lineage-specific constraints. Even within a single family, adaptation for a specialized ecology does not follow a singular deterministic path.

**Supplementary Information:**

The online version contains supplementary material available at 10.1186/s12862-021-01821-w.

## Background

Biologists have long been interested in the patterns and processes that underlie morphological diversity. Two major approaches in studying morphology are morphometrics and comparative anatomy. Morphometrics uses measurements or landmarks to quantify morphological variation, and has been used to study topics including ecomorphology [1, 2], modularity [2, 3], and convergent evolution [4, 5]. Comparative anatomy is often more time-intensive but offers a detailed view of structures that can provide further insight into systematics [6], ontogeny [7], biomechanics/function [8, 9], and adaptation [10, 11]. Increasingly, studies incorporate both methods [7, 8, 12, 13], which together provide a deeper understanding of evolution. For instance, geometric morphometrics can identify general convergence in shape, whereas anatomical study can investigate whether that convergence is the result of the same anatomical changes, implying similar developmental pathways, or different changes that may point to historical contingency. This imperfect convergence may imply variation in genetic pathways, functional trade-offs, many-to-one mapping, different ancestral states, or other lineage-specific constraints [4]. Here, we employ both approaches to study how skull morphology has changed for a specialized ecological lifestyle and examine convergence across multiple levels.

The skull serves many functions including protection of the brain and sensory organs, manipulation and breakdown of food, attachment for masticatory and axial muscles, and in some cases, locomotion or defense [14]. As a result, the skull is subject to many different evolutionary pressures, including ones that conflict with one another (e.g. burrowing performance and bite force; [15, 16]). In squamates (lizards and snakes), the skull has been found to vary with phylogenetic history [17, 18], allometry [19, 20], diet [21, 22], and habitat use [1, 2, 20, 23]. Burrowing squamates, in particular, occupy unique regions of morphospace and show convergence across families [2, 6, 24, 25]. In addition to having elongate bodies and reduced or no limbs, they possess many skull modifications related to head-first burrowing [26]. Common modifications include closure of the braincase, increased compactness of the skull roof, loss or reduction of various structures, and an overall streamlining of the skull [10, 24, 27, 28].

Despite this unique anatomy, studies of variation within burrowing squamates have been relatively limited. Soil properties, especially those related to hardness and penetrability, should impact an organism’s ability to burrow through it and thus different head shapes may offer advantages in particular substrates. Within squamates, most work on this topic has focused on amphisbaenians, the largest and most specialized group of fossorial squamates. Early work identified three specialized head shapes, in addition to the more generalized head shape, which are typically associated with different substrates [29]. More recent work has revealed that changes in skull width can impact burrowing performance between two closely related species [30] and that skull variation within the genus *Leposternon* is correlated with biogeographical variables related to soil type [31]. On the other hand, a study on Caribbean amphisbaenians found no relationship between skull shape and location (a proxy for soil type), and instead found a tight link with phylogenetic history and skull shape [18]. Together, these results indicate that although variation reflecting specific microhabitats can exist, this might be overshadowed by variation related to phylogenetic history in more distantly related species. Outside of amphisbaenians, gymnophthalmid lizards and acontine skinks have also shown a relationship between head shape and microhabitat [23, 32]. These studies indicate the existence of skull specialization for different substrates, but do not test it across independent derivations of fossoriality.

Scincidae (i.e., skinks) is one of the most speciose squamate families with more than 1700 species worldwide [33]. The family is also morphologically and ecologically diverse, occurring in a range of habitats on every continent apart from Antarctica. Whereas the typical skink has four well-developed limbs, limb reduction and loss are common, with numerous independent events of limb reduction estimated in the group [34, 35], including multiple times within some genera [36, 37]. Limb reduction is typically correlated with a fossorial lifestyle and accompanied by a suite of other adaptations [38, 39]. Despite this diversity, cranial descriptions of the group are few, with only ~ 250 species (~ 16% of the family) having osteological descriptions for complete skulls and/or individual disarticulated bones [11].

**Fig. 1:**
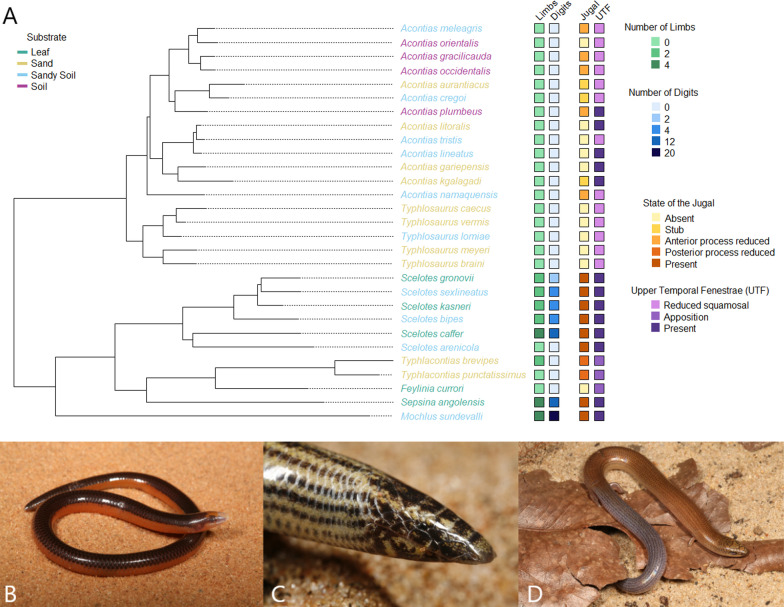
**A** Multi-locus squamate phylogeny [39] trimmed to show species in this study. Species names are colored by substrate. The data matrix shows number of limbs, number of digits, jugal condition, and condition of the upper temporal fenestrae, with boxes color-coded to indicate character state. Apposition refers to the closure of the upper temporal fenestrae through close contact of the squamosal with the parietal. Photos: **B** *Acontias litoralis*, **C** *Typhlacontias brevipes*, and **D** *Sepsina angolensis*. Credit to Johan Marais (**B** and **C**) and Luis M. P. Ceríaco (**D**)

We focused on skinks from southern Africa because of the repeated evolution of burrowing forms in this region. Other regions like southeast Asia and Australia also show diversity in fossorial skinks, although it tends to be limited to a single lineage [38, 40]. We looked at the genera *Acontias*, *Typhlosaurus*, *Scelotes*, *Sepsina*, *Feylinia*, *Typhlacontias*, and *Mochlus* (Fig. 1), which encompass three subfamilies and at least three independent derivations of semi-fossoriality/fossoriality [41]. *Acontias* and *Typhlosaurus* are in the subfamily Acontinae, which is the sister group to all other skinks and contains only limbless species [36, 41]. *Scelotes*, *Sepsina*, *Feylinia*, and *Typhlacontias* are in the subfamily Scincinae. The southern African scincines are all semi-fossorial and fossorial, and show great variation in specialization for burrowing and limb reduction [36]. *Mochlus* retains all four limbs and is within a clade of lygosomine skinks that includes semi-fossorial and non-fossorial genera [42]. Previous anatomical studies have looked at cranial osteology in *Acontias* [10, 43–47], *Typhlosaurus* [10, 43], *Scelotes* [48], and *Feylinia* [10, 49, 50]. By focusing on African skinks, where there have been multiple independent origins of fossoriality, we can tease apart the interplay of phylogenetic history, body size, and ecology on skull morphology and better understand the evolution of a specialized ecology.

We used micro-computed tomography (CT) scans to untangle the effects of the evolutionary history and ecological adaptation on skull morphology in seven genera of African skinks. First, using geometric morphometrics and phylogenetic comparative methods, we tested how cranial and mandibular shape varied with phylogenetic history, size, substrate, and degree of limb reduction, which is often used as a proxy for the degree of burrowing specialization [51]. Next, we described the skull anatomy of four African skinks, examining each bone in detail, to create an anatomical atlas and investigate variation across phylogenetic and functional levels. We looked at variation within a single genus (*Typhlacontias brevipes* and *Typhlacontias gracilis*) and compared them with a less specialized burrower in the same subfamily (*Sepsina alberti*) and with a more distantly related species that shares a more similar lifestyle (*Acontias occidentalis*). None of these species have been the subject of a detailed anatomical study before and two of the genera (*Typhlacontias* and *Sepsina*) have no such studies for the genus [11]. We also used these descriptions to assess whether fossorial adaptations show perfect convergence (similar overall traits due to the same changes to anatomy, implying similar developmental or genetic shifts).

## Results

**Fig. 2:**
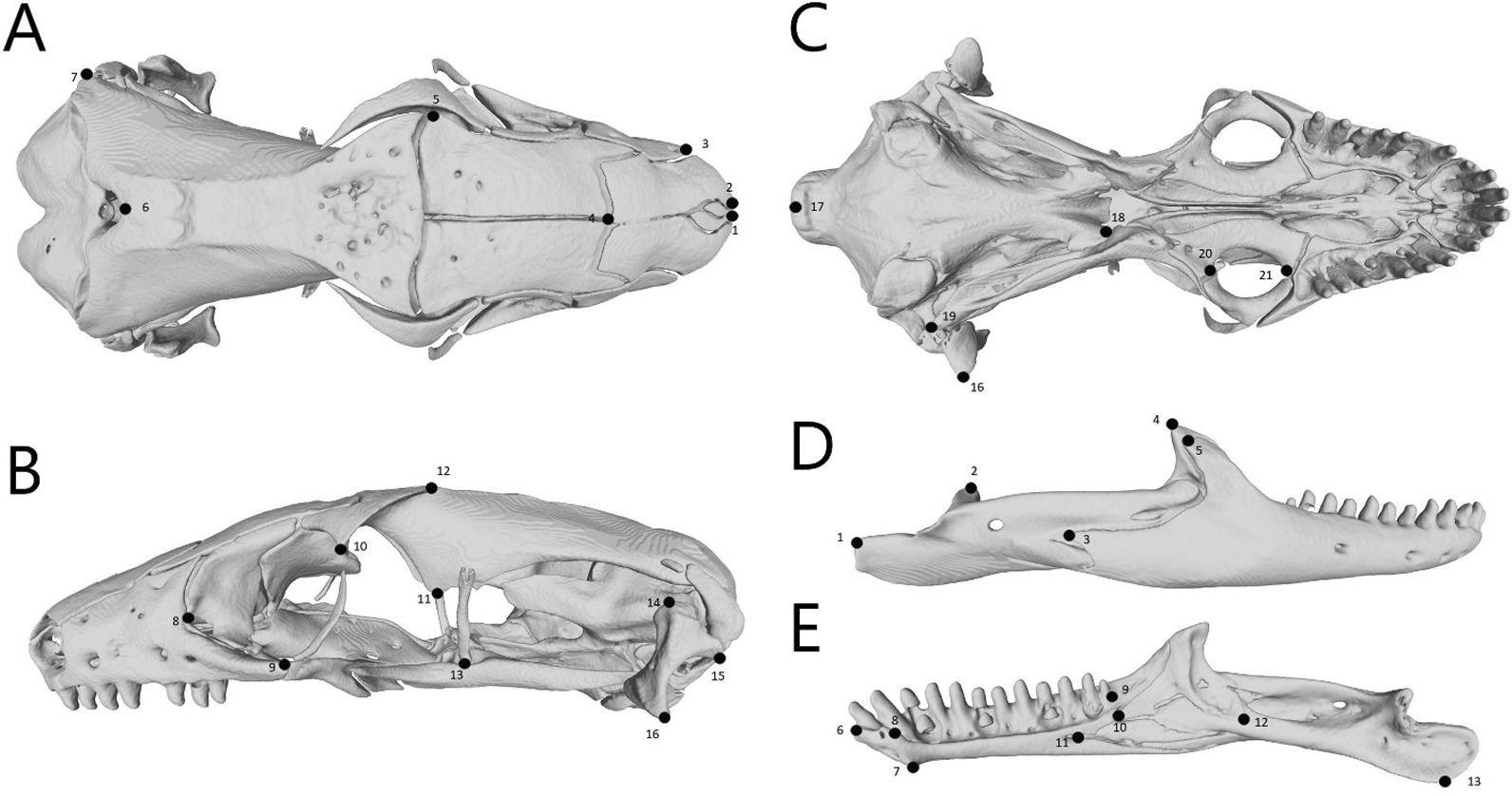
Location of landmarks on the cranium and right mandible of *Acontias occidentalis* (CAS 196430) in **A** dorsal; **B** lateral; **C** ventral; **D** labial; and **E** lingual views. We placed 21 fixed landmarks on the cranium and 13 on the mandible

We CT scanned 39 species from seven genera and segmented the cranium and lower jaw of each specimen. Image stacks, models, and metadata are available on Morphosource (https://www.morphosource.org/projects/00000C482). For our morphometrics, we used 21 fixed landmarks on the cranium and 13 on the mandible (Fig. 2). Because not all species were represented on the phylogeny, we ran analyses on three datasets for the cranium and mandible each. The first included all species and was analyzed with non-phylogenetic methods. The second excluded *Mochlus sundevallii* (the only species in its genus included in study) to run pairwise comparisons between genera and subfamilies. The third included only the species in the phylogeny and was analyzed with both non-phylogenetic and phylogenetic methods. For our phylogenetic analyses, we used a tree trimmed from a multi-locus squamate phylogeny [41]. We mapped burrowing-related traits on the tree to show their distribution in our sampling (Fig. 1). After, we provide anatomical descriptions for the four species we examined in greater detail. Details on scanning, segmentation, and analyses can be found in “Methods” section.

### Cranial morphometrics

The first two principal components PC1 and PC2 explained 42% and 13% of the cranial shape variation respectively (Additional file 1: Table S1). Species with a negative PC1 score possess taller crania with relatively short snouts and long frontals, and species with a positive score have depressed skulls with relatively long snouts and short frontals. The PC2 axis is related to the length of the braincase: species with negative scores have a relatively long braincase and species with positive scores have shorter braincases. The increase in length can be the result of a longer sphenoid, longer basipterygoid processes, or a combination of both features.

**Fig. 3:**
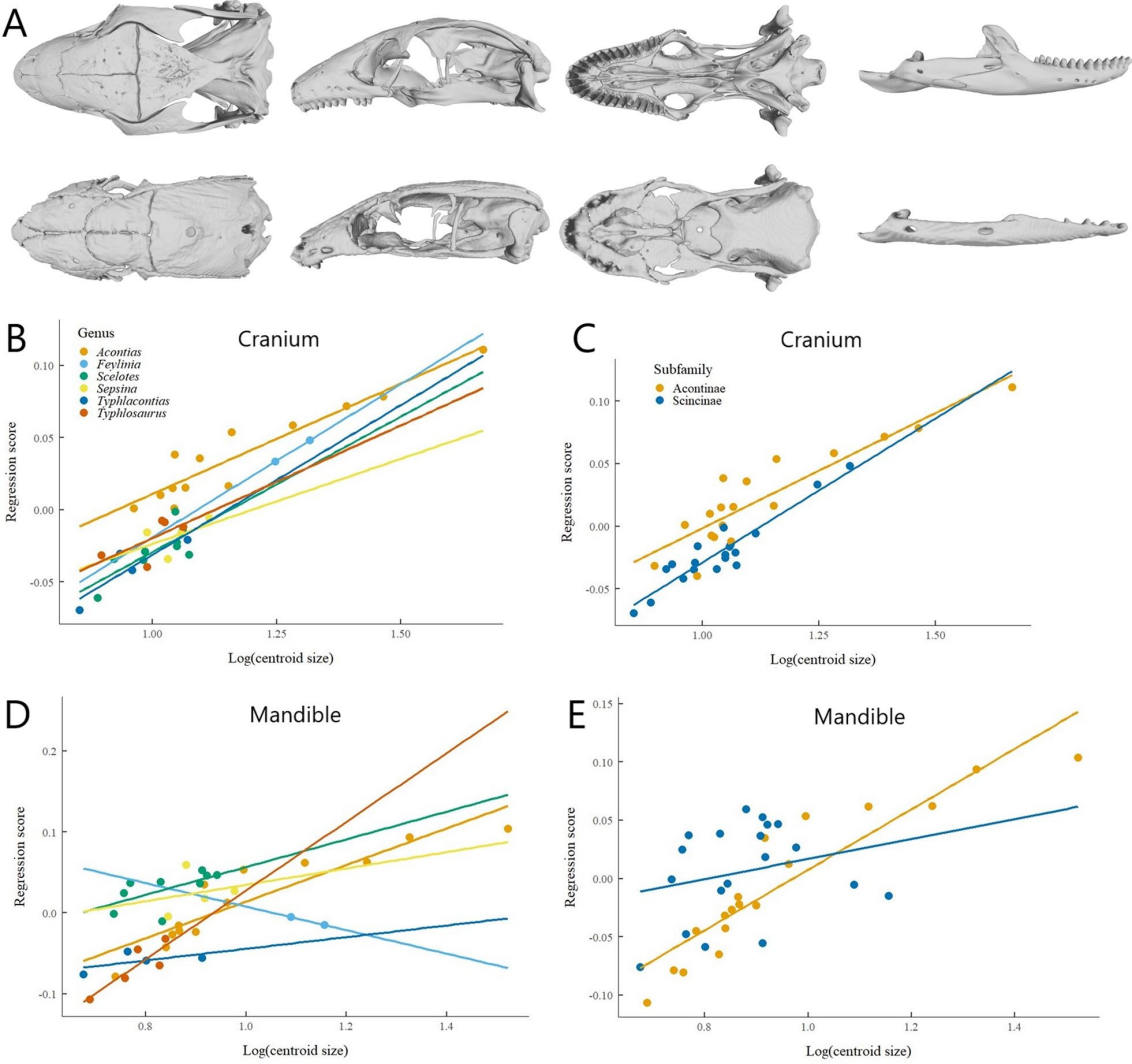
The relationship between size and shape in African burrowing skinks. **A** The largest skull *Acontias plumbeus* (MCZ R-18358, top row, skull length = 31 mm) and the smallest skull *Typhlacontias rohani* (MCZ R-190458, bottom row, skull length = 5.13 mm) in dorsal, lateral, ventral, and labial views. Multivariate regression between centroid size and cranial shape (Regression score). Ordinary least squares regression lines are displayed for each **B** genus, excluding *Mochlus* which only had one species; and **C** subfamily except for Lygosominae, which only had one species. Multivariate regression between centroid size and mandibular shape (Regression score). Ordinary least squares regression lines are displayed for each **D** genus; and **E** subfamily

The multivariate regression found a significant relationship between cranial shape and centroid size (*p* = 0.0034). In general, smaller skinks have relatively longer braincases, less distance between the quadrate and braincase, and narrow rectangular parietals. Larger skinks have relatively short braincases, greater distance between the quadrate and braincase, and square or hourglass-shaped parietals (Fig. 3A). When comparing patterns of allometry between genera (Additional file 1: Table S2), *Acontias* and *Scelotes* both significantly differed in slope vector length from *Typhlacontias* and *Typhlosaurus*, and *Sepsina* differed in slope vector length from *Typhlosaurus* (Fig. 3B). The allometric patterns for Acontinae and Scincinae significantly differed both in slope vector length (*p* = 0.0011) and slope vector orientation (*p* = 0.0082; Fig. 3C). In the reduced dataset, both the multivariate and phylogenetic regressions found a significant relationship between cranial shape and centroid size (Additional file 1: Table S4). There were significant interactions between centroid size and genus, subfamily, digits, and substrate (Additional file 1: Tables S3, S4); therefore, pairwise comparisons were run both with and without centroid size as a covariate to account for this.

**Fig. 4:**
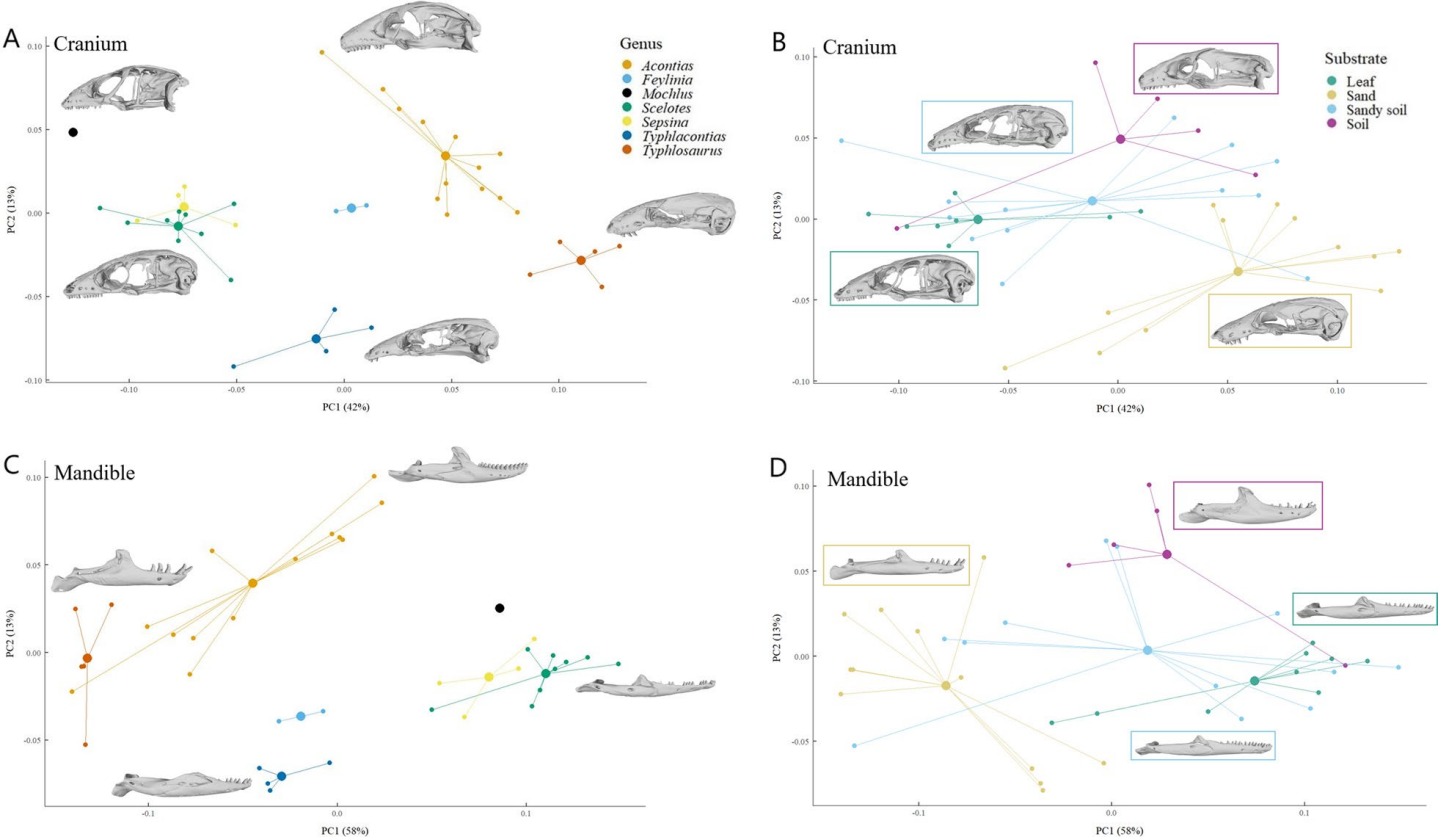
PCA plots showing the relationship between cranial shape and **A** genus and **B** substrate; and mandibular shape and **C** genus and **D** substrate. Lines connect each species point to the group mean. For the genus PCAs, the pictured crania, from top to bottom and left to right, are *Mochlus sundevallii* (MCZ R-28682), *A. plumbeus*, *Scelotes gronovii* (CAS 173306), *Typhlacontias brevipes* (CAS 224004), and *Typhlosaurus vermis* (CAS 196406). The pictured jaws are *A. plumbeus*, *T. vermis*, *Scelotes sexlineatus* (CAS 206813), and *T. brevipes*. For the substrate plots, the specimen closest to the group mean is shown. The crania are *Scelotes arenicola* (MCZ R-21292), *Acontias gracilicauda* (CAS 147466), *Scelotes kasneri* (CAS 207016), and *Acontias kgalagadi* (CAS 125809). The jaws are *A. gracilicauda*, *Acontias litoralis* (CAS 206810), *Sepsina alberti* (CAS 263923), and *Sepsina copei* (CAS 263921)

There was a significant relationship between cranial shape and both genus (*p* < 0.001) and subfamily (*p* < 0.001) in the full dataset. Acontinae and Scincinae retained significantly different cranial shapes after size correction (*p* < 0.001). Every genus was significantly different from every other genus prior to size correction except for *Scelotes* and *Sepsina* (Additional file 1: Table S5). Most results remained unchanged after size correction except *Acontias* and *Typhlosaurus* were no longer different. The PCA supported these results, as genera formed discrete clusters in morphospace except for *Scelotes* and *Sepsina* (Fig. 4A). The ANCOVA for the reduced dataset also showed a significant relationship between cranial shape and both genus and subfamily; however, the PGLS did not (Additional file 1: Table S4). The K-statistic’s generalization for multivariate data suggested that less phylogenetic signal was present than expected under a Brownian motion (BM) model of trait evolution for cranial shape (K = 0.57592, *p* < 0.001).

We found a significant relationship between cranial shape and both limbs (*p* < 0.001) and digits (*p* < 0.001) in the full dataset. This result was driven by a large significant difference between limbless skinks and the other limb states (*p* < 0.001). Skinks with two limbs and four limbs did not differ from each other. Although both limbs and digits showed a significant relationship with cranial shape in the ANCOVA for the reduced dataset, these relationships ceased to be significant when corrected for phylogeny (Additional file 1: Table S4).

A significant relationship was found between cranial shape and substrate in the full dataset (*p* < 0.001; Fig. 4B). Before size correction, all but two pairs, sandy soil with soil and with leaf litter, showed a significant difference (Additional file 1: Table S6). After size correction, the only significant differences left were between sand with leaf litter and with sandy soil (Additional file 1: Table S6). The ANCOVA and PGLS both found a significant relationship between cranial shape and substrate in the reduced dataset (Additional file 1: Table S4). In the ANCOVA (Additional file 1: Table S7), prior to size correction, sand burrowers were significantly different from the other three substrates and skinks in leaf litter were different from those in soil. When corrected for size, sand burrowers were significantly different from those in leaf litter and sandy soil, but not soil. In the PGLS (Additional file 1: Table S8), prior to size correction, sand was significantly different from sandy soil and soil. After size correction, only sand and sandy soil were significantly different.

### Mandibular morphometrics

The first two principal components PC1 and PC2 explained 58% and 13% of the mandibular shape variation respectively in the full dataset (Additional file 1: Table S9). The PC1 axis describes the dentary. Species with a negative score have a longer dentary, with the most posterior point along the lateral side of the mandible. They also have a relatively short tooth row. Species with a positive score have a shorter dentary, with the most posterior point along the ventral side of the mandible and a long tooth row extending to the coronoid. The PC2 axis describes both the coronoid process of the dentary and the coronoid. Species with a negative score have a very short coronoid and species with a positive score have a tall coronoid and coronoid process.

The multivariate regression for the full dataset showed a significant relationship between mandibular shape and centroid size (*p* = 0.0167). Smaller skinks have relatively narrow jaws and larger skinks have thicker, more robust jaws (Fig. 3A). When comparing patterns of allometry between genera, there were significant pairwise differences between *Acontias* with *Typhlacontias* and *Typhlosaurus*, *Feylinia* with *Typhlosaurus*, *Scelotes* with *Typhlacontias* and *Typhlosaurus*, and *Sepsina* with *Typhlosaurus* (Fig. 3D; Additional file 1: Table S10). The patterns of allometry between Acontinae and Scincinae did not differ in slope vector length (*p* = 0.2739), but they were significantly different in slope vector orientation (*p* = 0.039; Fig. 3E). In the reduced dataset, both the multivariate and phylogenetic regressions showed a significant relationship between cranial shape and centroid size (Additional file 1: Table S12). There were also significant interactions between centroid size and all other variables (Additional file 1: Tables S11, S12).

We found a significant relationship between mandibular shape and both genus (*p* < 0.001) and subfamily (*p* < 0.001) in the full dataset. Acontinae and Scincinae had significantly different mandibular shapes even after size correction (*p* < 0.001). Prior to size correction, there were many significant pairwise differences between genera (Additional file 1: Table S13) including *Acontias* and all genera except *Feylinia*, *Feylinia* from *Scelotes* and *Typhlosaurus*, *Scelotes* from *Typhlacontias* and *Typhlosaurus*, *Sepsina* from *Typhlacontias* and *Typhlosaurus*, and *Typhlacontias* from *Typhlosaurus*. After size correction, the remaining differences were: *Acontias* from *Scelotes* and *Sepsina*, *Feylinia* from *Scelotes* and *Sepsina*, *Scelotes* from *Typhlacontias* and *Typhlosaurus*, *Sepsina* from *Typhlosaurus*, and *Typhlacontias* from *Typhlosaurus*. The PCA supported these relationships, with many genera occupying unique regions of morphospace with low overlap (Fig. 4C). The ANCOVA, but not the PGLS, for the reduced dataset supported a significant relationship between mandibular shape and both genus and subfamily (Additional file 1: Table S12). The K-statistic’s generalization for multivariate data suggested that less phylogenetic signal was present than expected under a BM evolutionary model of trait evolution for mandibular shape (K = 0.62051, p-value < 0.001).

In the full dataset, we found a significant relationship between mandibular shape and the number of limbs (*p* < 0.001) and digits (*p* < 0.001). Species with no limbs were significantly different from species with either two or four limbs (*p* < 0.001), but species with two limbs were not different from species with four limbs. Although both limbs and digits showed a significant relationship with mandibular shape in the ANCOVA for the reduced dataset, these relationships were not significant when corrected for phylogeny (Additional file 1: Table S12).

There was a significant relationship between mandibular shape and substrate in the full dataset (*p* < 0.001; Fig. 4D). Skinks in sand were significantly different from skinks in leaf litter, sandy soil, and soil before and after size correction (Additional file 1: Table S14). The ANCOVA and PGLS both found a significant relationship between mandibular shape and substrate in the reduced dataset (Additional file 1: Table S12). In the ANCOVA (Additional file 1: Table S15), sand burrowers had significantly different mandibles from those in leaf litter, sandy soil, and soil. After size correction, sand burrowers only varied from those in leaf litter and sandy soil. In the PGLS (Additional file 1: Table S16), prior to size correction, burrowers in sand were significantly different from those in sandy soil and soil. After size correction, they were different only from those in sandy soil.

### Anatomical descriptions

 Below, we provide anatomical descriptions for the skulls of four species—*T. brevipes* (CAS 224004), *T. gracilis* (MCZ R-18023), *S. alberti* (CAS 263923), and *A. occidentalis* (CAS 196430)—based on examination of a single adult specimen. We begin with a general overview of the skull before moving on to descriptions of each individual bone.

#### General skull morphology

**Fig. 5:**
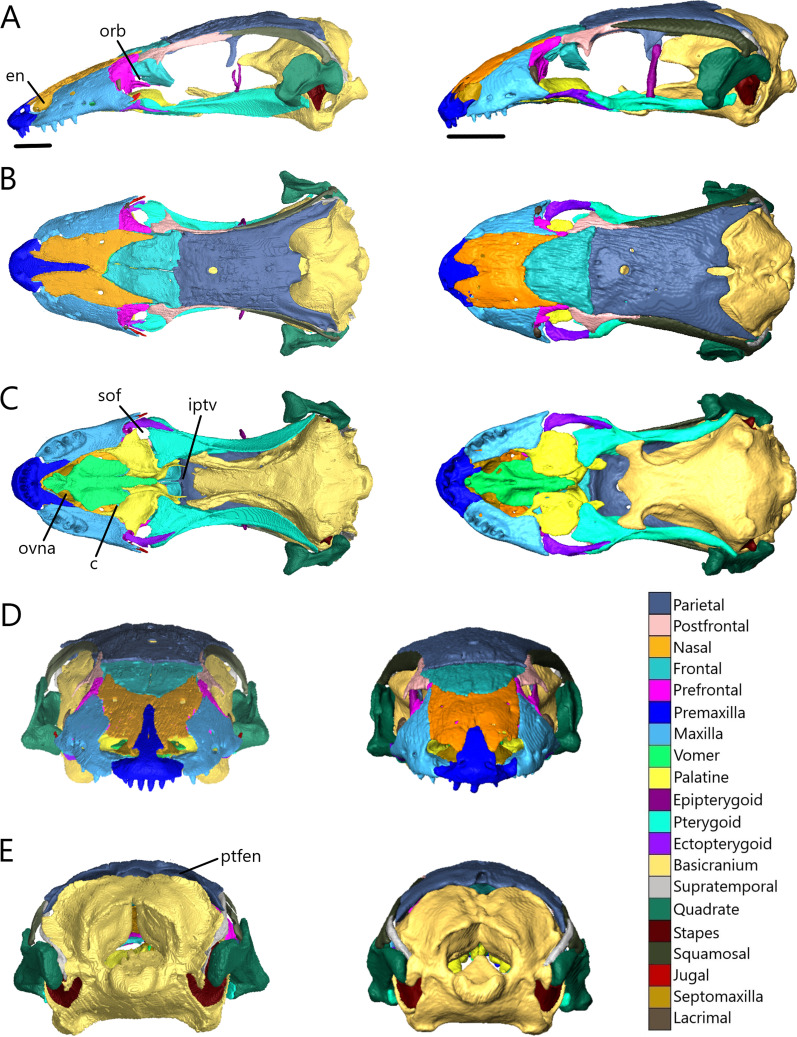
Articulated skulls of *T. brevipes* (left) and *T. gracilis* (right). Views are: **A** lateral; **B** dorsal; **C** ventral; **D** anterior; **E** posterior. c, choanae; en, external nares; fov, fenestra ovalis; iptv, interpterygoid vacuity; orb, orbit; ovna, opening for the vomeronasal apparatus; ptfen, posttemporal fenestra; sof, suborbital fenestra. Scale bars = 1 mm

**Fig. 6:**
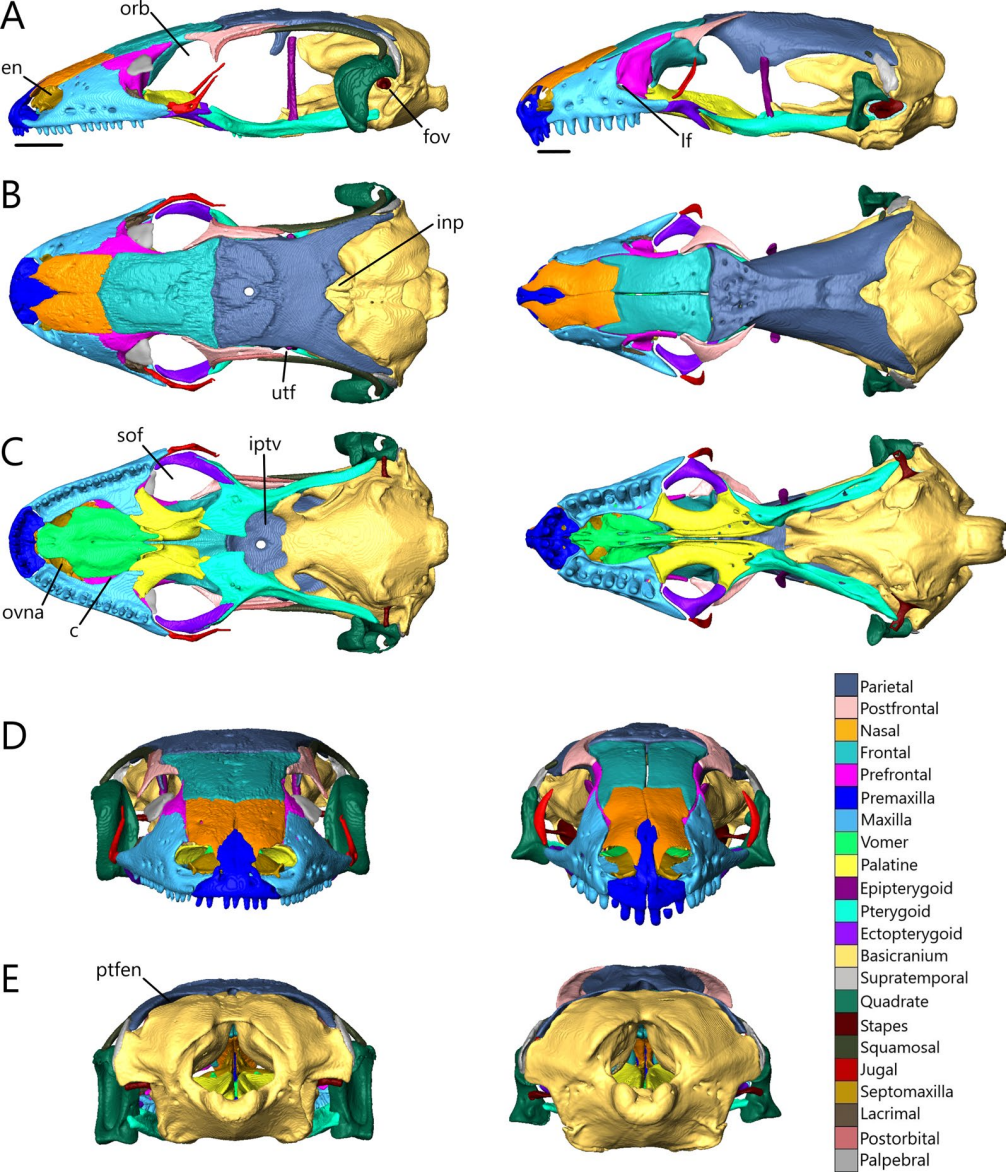
Articulated skulls of *S. alberti* (left; CAS 263923) and *A. occidentalis* (right; CAS 196430). **A** Lateral; **B** dorsal; **C** ventral; **D** anterior; **E** posterior. c, choanae; en, external nares; fov, fenestra ovalis; inp, interparietal; iptv, interpterygoid vacuity; lf, lacrimal foramen; orb, orbit; ovna, opening for the vomeronasal apparatus; ptfen, posttemporal fenestra; sof, suborbital fenestra; utf, upper temporal fenestra. Scale bars = 1 mm

The skull length (from the most anterior point of the premaxilla to the most posterior point of the braincase on the ventral side) and width (from the lateral extent of one quadrate to the other) respectively are 8.33 mm (8.4% SVL) and 4.26 mm for *T. brevipes*, 6.03 mm (7.6% SVL) and 2.93 mm for *T. gracilis*, 9.06 mm (11.2% SVL) and 4.5 mm for *S. alberti*, and 13.51 mm (7.5% SVL) and 6.25 mm for *A. occidentalis*. The skull is similarly proportioned with the length twice or more the width. The skull of *T. brevipes* is triangular and wedge-shaped and the skull of *T. gracilis* forms a rounded wedge (Fig. 5A). The lateral view of *S. alberti* is similar to *T. gracilis* although with a longer snout (Fig. 6A). The skull of *A. occidentalis* has a more robust, rectangular snout (Fig. 6A). In dorsal view, the snout is more rounded in *S. alberti* and more pointed in the other three (Figs. 5B and 6B). The palate shows closure in all species with the most closure in *A. occidentalis* and *T. brevipes*, and the least in *S. alberti* (Figs. 5C and 6C).

Most bones are present in all examined species. Exceptions are the postorbitals, interparietals, and palpebrals, which are only present in *S. alberti*. Osteoderms are present in all species. Some are fused to the skull roofing bones, and digitally removing them has created grooves and pits across the dorsal surface of the nasals, frontals, and parietal.

The external nares (Figs. 5 and 6A) are oblong and flattened in *T. brevipes*, rounded in *T. gracilis*, oblong in *S. alberti*, and oval with a domed dorsal margin in *A. occidentalis*. They are separated by the premaxilla and nasals, and extend back to the nasal-maxillary suture. The orbits are delimited by the prefrontal anteriorly, postfrontal posteriorly, frontal medially, jugal laterally (also posteriorly in *S. alberti* and *A. occidentalis*), and maxilla and ectopterygoid ventrally. The frontal does not close the medial wall of the orbit in *S. alberti*. The orbits are circular in *S. alberti* (21% of skull length), and ovoid in *A. occidentalis* (20%), *T. gracilis* (18%) and *T. brevipes* (13%). The lacrimal foramen is bordered by the prefrontal medially and ventrally, and lacrimal and maxilla laterally. In *T. gracilis*, the prefrontal does not curve as far and the lacrimal foramen is delimited by the maxilla and jugal ventrally. The suborbital fenestrae (Figs. 5, 6C) are smallest in *T. brevipes* and largest in *S. alberti*. They are bordered by the ectopterygoid laterally, palatine anteromedially, and pterygoid posteromedially (except in *A. occidentalis* where the pterygoid does not participate). In *S. alberti*, they are also bordered by the maxilla anterolaterally. The choanae are bordered by the vomer anteromedially and palatine posteriorly, and the openings for the vomeronasal apparatus are bordered by the vomer medially and maxilla laterally. The interpterygoid vacuity begins between the medial margins of the palatines and widens posteriorly following the curvature of the pterygoids to the sphenoid. It is largest in *S. alberti* and narrowest in *A. occidentalis*. The upper temporal fenestrae (Fig. 6B) are narrow and subtriangular in *S. alberti*. They are bordered by the parietal medially, postfrontal and postorbital anteriorly, and squamosal laterally. The posterior region of the squamosal and parietal contact one another, closing the fenestrae and making them smaller compared to non-fossorial skinks [14]. These fenestrae are absent in *Typhlacontias*, where they are closed by the apposition of the parietal and squamosal, and in *A. occidentalis*, where the postorbital and squamosal have been reduced. The upper temporal arch is present in the scincines. The posttemporal fenestrae (Figs. 5, 6E) are largest in *T. brevipes*, then in *S. alberti*, and smallest in *T. gracilis* and *A. occidentalis* where the parietal nearly contacts the braincase. The fenestra ovalis (located behind the stapes) is large and oval in *Typhlacontias*, small and circular in *S. alberti*, and large and elliptical in *A. occidentalis*.

**Fig. 7:**
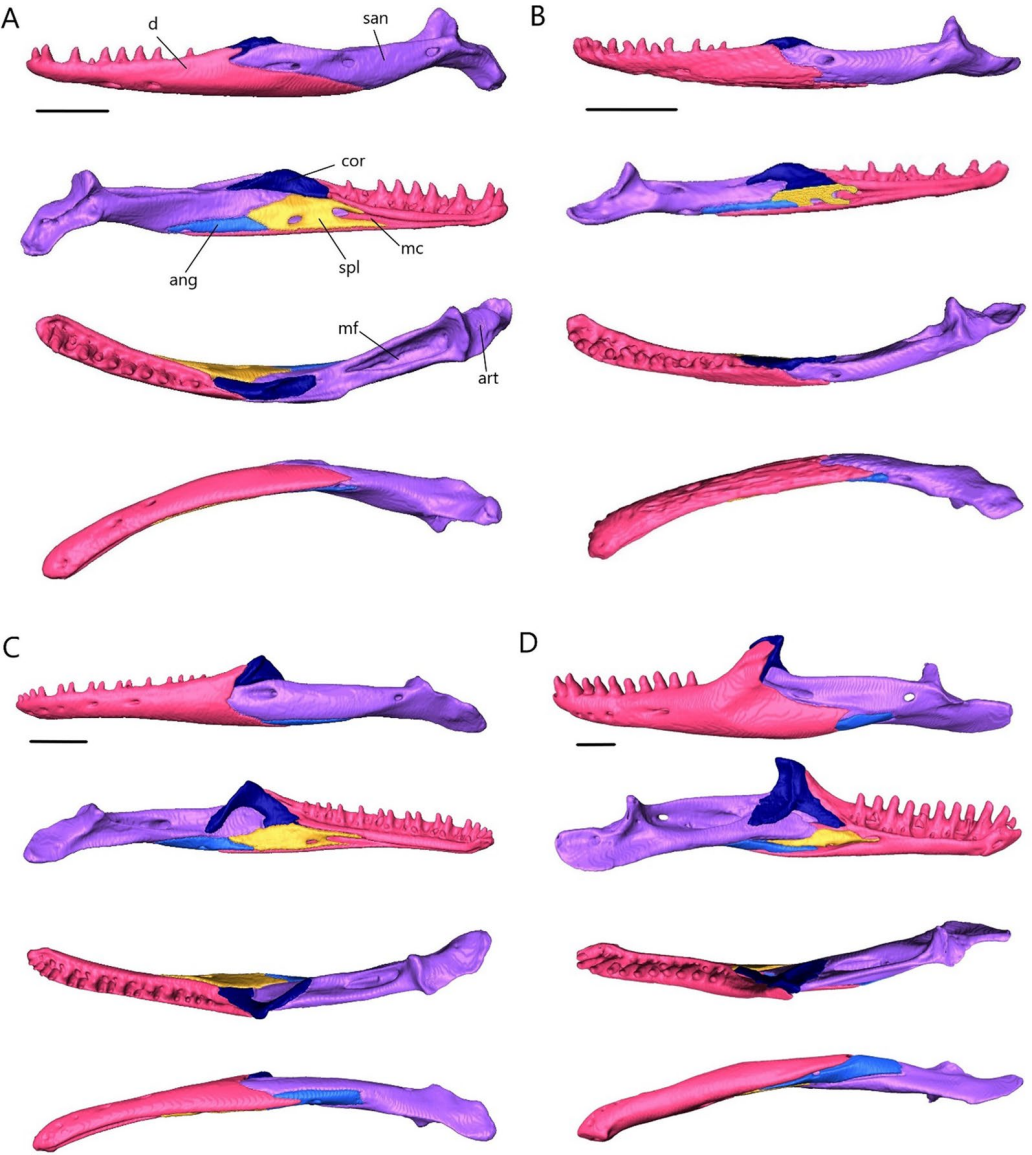
Articulated mandibles of **A** *T. brevipes*; **B** *T. gracilis*; **C** *S. alberti*; **D** *A. occidentalis*; in lateral, medial, dorsal, and ventral views from top to bottom. ang, angular; art, articular of compound bone; cor, coronoid; d, dentary; mc, Meckelian canal; mf, mandibular fossa; san, surangular of compound bone; spl, splenial. Scale bars = 1 mm

The lower jaw (Fig. 7) consists of four discrete elements: the angular, coronoid, dentary, and splenial, and two fused elements forming the compound bone: the articular and surangular. The lower jaw shows the most curvature in *Typhlacontias* and the least in *S. alberti*. The Meckelian canal runs open along the lingual side of the dentary in the scincines and is then enclosed by the splenial and surangular where it continues to the mandibular fossa. It is enclosed along its entire length in *A. occidentalis*. The mandibular fossa is larger in *T. brevipes* and *A. occidentalis* and smaller in *T. gracilis* and *S. alberti*.

#### Description of isolated dermatocranial bones

##### Premaxilla

**Fig. 8:**
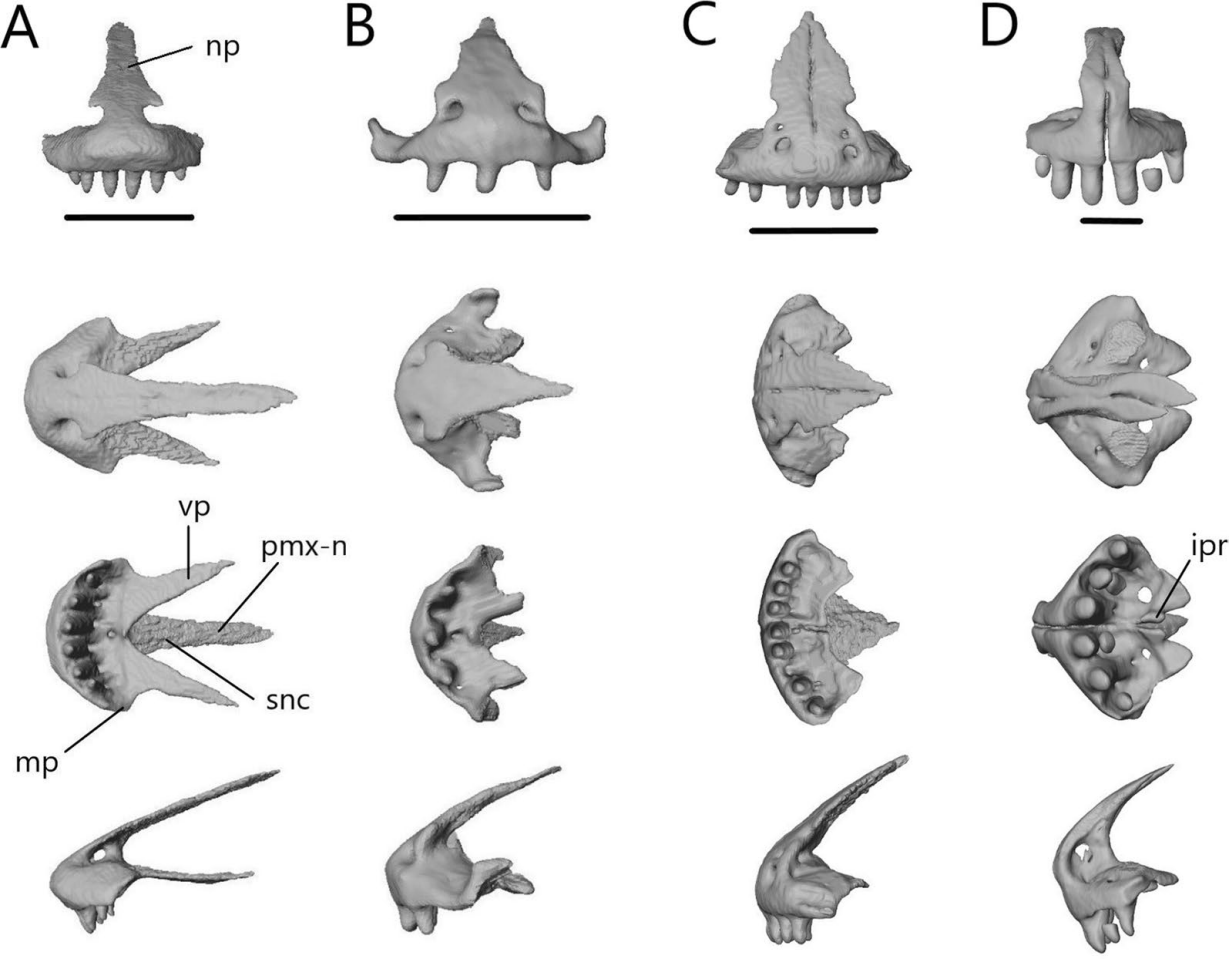
Isolated premaxillae of **A** *T. brevipes*; **B** *T. gracilis*; **C** *S. alberti*; **D** *A. occidentalis*; in anterior, dorsal, ventral, and lateral views from top to bottom. mp, maxillary process; np, nasal process; pmx-n, nasal facet of the premaxilla; snc, septonasal crest; vp, vomerine process. Scale bars = 1 mm

The premaxilla (Fig. 8) is the anterior-most bone of the skull and can be fused or paired. It consists of a curved, ventral alveolar portion and a dorsal nasal process. It contacts the nasals posterodorsally and the maxillae posteroventrally. In *A. occidentalis*, the premaxilla also contacts the vomer and septomaxilla posteriorly whereas in *T. gracilis*, it contacts the septomaxilla posteriorly. The teeth are pleurodont, isodont, and cylindrical.

*Typhlacontias brevipes* has a premaxilla fused at the base with a slight suture on the nasal process (Fig. 8A) whereas *T. gracilis* possesses a fused premaxilla with no trace of a suture (Fig. 8B). The nasal process is triangular. The base of the nasal process is wider in *T. gracilis* with rounded corners and narrow in *T. brevipes* with pointed corners. The nasal process overlaps the nasals, extending roughly half their length in *T. brevipes* and one-third in *T. gracilis*. The septonasal crest is a raised ridge on the ventral side of the nasal process. The vomerine processes extend posterolaterally. They are longer in *T. brevipes* where they border the opening to the vomeronasal apparatus. In *T. gracilis*, the vomerine process is much shorter. In both species, they are overlapped by the maxillae dorsally. Lateral to the vomerine processes, the premaxilla has small maxillary processes which articulate with the anterolateral processes of the maxilla. They are larger and curve dorsally in *T. gracilis*. *Typhlacontias brevipes* has 6 tooth loci (6 teeth present) and *T. gracilis* has 7 tooth loci (3 teeth present).

The premaxilla of *S. alberti* is paired (Fig. 8C). The nasal process is triangular and broad. It overlaps roughly one-third of the nasal length. Anteriorly, each premaxilla has two foramina, a smaller dorsal one and a larger ventral one. The vomerine process is small, triangular, and overlapped by the maxillae. As in *T. gracilis*, it reaches the anterior margin of the opening for the vomeronasal apparatus. Laterally, the premaxilla has a small maxillary process, which has a distinct facet dorsally where the maxilla meets it. *Sepsina alberti* has 9 tooth loci (7 teeth present). The teeth have slight constrictions near the middle and are smaller than in *Typhlacontias*.

The premaxilla is paired in *A. occidentalis* (Fig. 8D). The nasal process has a constriction near its anterior end, after which it assumes a teardrop shape. The nasal process extends roughly halfway up the nasals. The vomerine process is broad and triangular. It contacts the vomer dorsally and maxilla laterally. Medially, the premaxillae have small ventral knobs which form the incisive process. There is no distinct maxillary process. Instead, the lateral edge has a slight indent which the maxilla fits into. There are 7 premaxillary tooth loci (7 teeth present). The teeth are similar to *Typhlacontias* although more robust.

##### Nasal

**Fig. 9:**
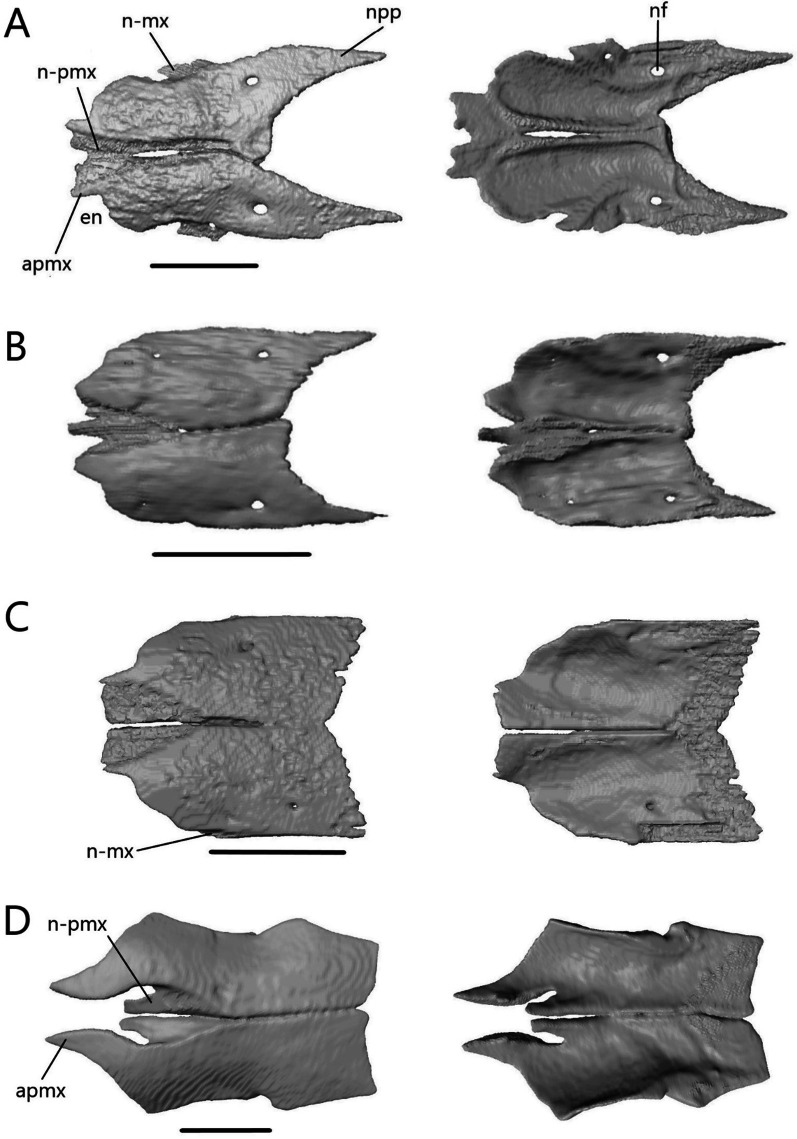
Isolated nasals of **A** *T. brevipes*; **B** *T. gracilis*; **C** *S. alberti*; **D** *A. occidentalis*; in dorsal (left) and ventral (right) views. apmx, anteromedial premaxillary process; en, external nares; n-mx, maxilla facet of the nasal; n-pmx, premaxilla facet of the nasal; nf, nasal foramen; npp, posterior process of the nasal. Scale bars = 1 mm

The nasal (Fig. 9) is thin and paired, with medial contact between the two. It contacts the premaxilla anteromedially, maxilla laterally, frontal posteriorly, prefrontal posterolaterally, and the septomaxilla ventromedially.

The width of a single nasal is about 32% of the length in *Typhlacontias*. The anteromedial premaxillary process has a shelf facet, which is overlapped by the nasal process of the premaxilla. This shelf extends further posteriorly in *T. brevipes* (Fig. 9A). In *T. brevipes*, the anteromedial premaxillary process is more pointed and the anterior margin bordering the nares is more sharply emarginated than in *T. gracilis* (Fig. 9B). The anteromedial premaxillary process of *T. gracilis* is rounded. The triangular posterior process overlaps a depressed region of the frontal, although this overlap is minimal in *T. gracilis*. The posterior process extends up two-thirds of the frontal in *T. brevipes* and slightly less than half in *T. gracilis*. The maxilla overlaps a small shelf on the dorsolateral surface of the nasal in *T. brevipes*, but in *T. gracilis*, the maxilla simply abuts the nasal. Both species have a nasal foramen posteriorly.

The width of a single nasal is about 42% of the length in *S. alberti*, giving the combined nasals an almost square shape (Fig. 9C). The anteromedial premaxillary process is triangular and pointed, even more than in *T. brevipes*. It bears a broad shelf facet, which contacts the nasal process of the premaxilla, and has a flattened anterior margin. The border with the external nares is emarginated though not as sharply as in *T. brevipes*. The posterior margin lacks a distinct posterior process. Anteriorly, the nasal has a very small shelf which is overlapped by the maxilla, but for most of its length, the nasal simply abuts the maxilla as in *T. gracilis*. There is a foramen on the left nasal in the same location as in *Typhlacontias* although smaller.

The width of a single nasal is about 30% of the length in *A. occidentalis* (Fig. 9D). Unlike in the scincines, where the shelf facet is nearly continuous with the anteromedial premaxillary process, the shelf facet is not connected to the anteromedial premaxillary process in *A. occidentalis*. The shelf has curved sides to accommodate the premaxilla. The anteromedial premaxillary process is larger too, extending past the shelf. It curves medially, ending in a sharp point. The posterior side is slightly curved, and lacks a distinct process. The nasal also lacks a maxillary shelf. A small indentation in the lateral margin forms a foramen that is closed ventrally by the maxilla.

##### Maxilla

**Fig. 10:**
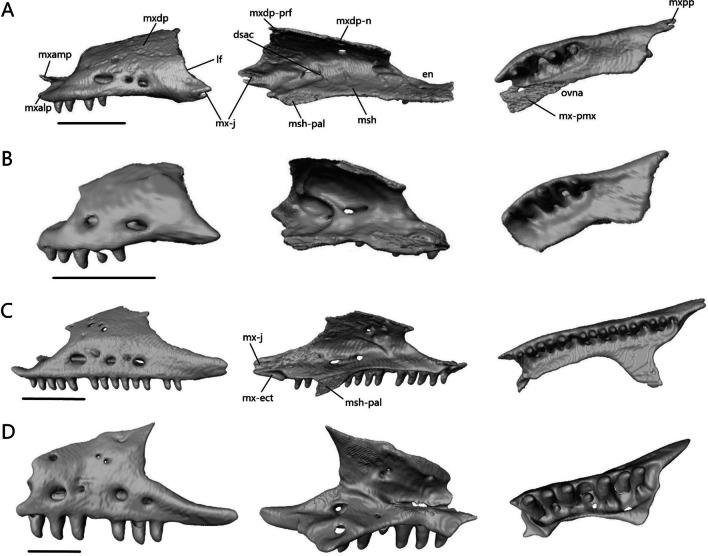
Isolated left maxilla of **A** *T. brevipes*; **B** *T. gracilis*; **C** *S. alberti*; **D** *A. occidentalis*; in lateral, medial, and ventral views from left to right. dsac, dorsal opening for the superior alveolar canal; ipr, incisive process; lf, lacrimal foramen; msh, medial shelf of the maxilla; msh-pal, palatine facet of the medial shelf; mx-ect, ectopterygoid facet of the maxilla; mx-j, jugal facet of the maxilla; mx-pmx, premaxilla facet of the maxilla; mxalp, anterolateral process of the maxilla; mxamp, anteromedial process of the maxilla; mxdp, dorsal process of the maxilla; mxdp-n, nasal facet of the dorsal process; mxdp-prf, prefrontal facet of the dorsal process; mxpp, posterior process of the maxilla; ovna, opening for the vomeronasal apparatus. Scale bars = 1 mm

The maxilla (Fig. 10) is a paired bone consisting of a tooth-bearing medial shelf and a large dorsal process that forms the lateral wall of the snout. It contacts the premaxilla anteriorly, nasal dorsomedially, prefrontal and lacrimal posteriorly, septomaxilla medially, jugal and ectopterygoid posteromedially (except in *A. occidentalis* where there is no contact with the jugal), and palatine ventromedially. The maxillary teeth are similar to the premaxillary teeth.

*Typhlacontias brevipes* has two anterior processes—the anterolateral process and the anteromedial process—which form a U-shaped indentation in ventral view (Fig. 10A). The anteromedial process curves dorsally and overlaps the premaxilla’s vomerine process. The anterolateral process contacts the maxillary process of the premaxilla. The anterolateral process of *T. gracilis* is similar, but the species lacks a distinct anteromedial process (Fig. 10B). The medial shelf forms the floor of the external naris anterodorsally, the anterolateral border of the choana, and the lateral border of the opening for the vomeronasal apparatus. Its curvature follows the shape of the vomer but never contacts it. The gaps between the vomer and maxilla are larger in *T. gracilis* (Fig. 5C). The medial shelf contacts the palatine posteromedially. The dorsal opening for the superior alveolar canal is at the junction of the medial shelf and the dorsal process, and located two-thirds of the length of the maxilla. The dorsal process curves dorsomedially to contact the nasal anteriorly and prefrontal posteriorly. In *T. gracilis*, there is greater contact with the prefrontal. The dorsal process forms the posterolateral border of the external naris and part of the lateral border of the lacrimal foramen. It tapers posteriorly to form the short and blunt posterior process. The posterior process contacts the ectopterygoid posteromedially in *T. brevipes* and posteriorly in *T. gracilis* where the posterior process is shorter. It also contacts the jugal medially. There are three large foramina along the lateral side in *T. brevipes* and two in *T. gracilis*. The teeth are limited to the anterior half. *Typhlacontias brevipes* has 4 and 4 (left and right) maxillary tooth loci (4 teeth present), and *T. gracilis* has 5 and 5 maxillary tooth loci (8 teeth present).

As in *T. brevipes*, the maxilla of *S. alberti* (Fig. 10C) has two anterior processes although the gap formed between them is broader. The anteromedial process curves dorsomedially, resting between the premaxilla and vomer on its ventral side and the septomaxilla on its dorsal side. It has a smaller area of overlap than in *T. brevipes*. The anterolateral process extends further anteriorly and contacts the maxillary process of the premaxilla. The medial shelf is narrower than in *Typhlacontias* except for the expanded posterior lappet which contacts the palatine dorsally. This lappet curves medially, is subtriangular, and provides greater contact with the palatine than seen in *Typhlacontias*. The dorsal opening for the superior alveolar canal is about halfway down the maxilla. The dorsal process curves dorsomedially, as in *Typhlacontias*, but rather than a straight dorsal edge, the dorsal edge curves posteriorly. It tapers to form the long posterior process. The posterior process contacts the jugal anteromedially and the ectopterygoid posteromedially. There are three large foramina along the lateral side with two smaller foramina between them. The teeth extend up to the posterior process. There are more than in *Typhlacontias*, 16 and 15 tooth loci (27 teeth present), although they are similar in shape and size.

Like *T. brevipes* and *S. alberti*, *A. occidentalis* has anterolateral and anteromedial processes, which are separated by a shallow W-shaped indent (Fig. 10D). The anteromedial process overlaps the vomerine process of the premaxilla and is overlapped by the septomaxilla. It has a wide base with a triangular point. The anterolateral process ends in a rounded knob which contacts the premaxilla. The medial shelf is narrower than in the others and taken up almost entirely by the tooth loci. The vomer overlaps the medial shelf, closing the anterior palate except for the openings for the vomeronasal apparatus and choanae (Fig. 6C). The depressed shelf for the palatine is not as large as in *S. alberti*, but it is more distinct than in *Typhlacontias*. The dorsal opening for the superior alveolar canal is located at two-thirds of the length of the maxilla. The dorsal process has an almost vertical anterior edge. It then curves dorsomedially, contacting the nasal anterodorsally, frontal posterodorsally, and prefrontal posteriorly. It ends in a sharp point. The posterior process is long, but shorter than in *S. alberti*. It contacts the ectopterygoid medially along its entire length. There are three large foramina on the lateral side, with another small foramen near the anterior-most of these. The teeth extend up to the posterior process. In total, *A. occidentalis* has 8 and 7 maxillary tooth loci (12 teeth present).

##### Prefrontal

**Fig. 11:**
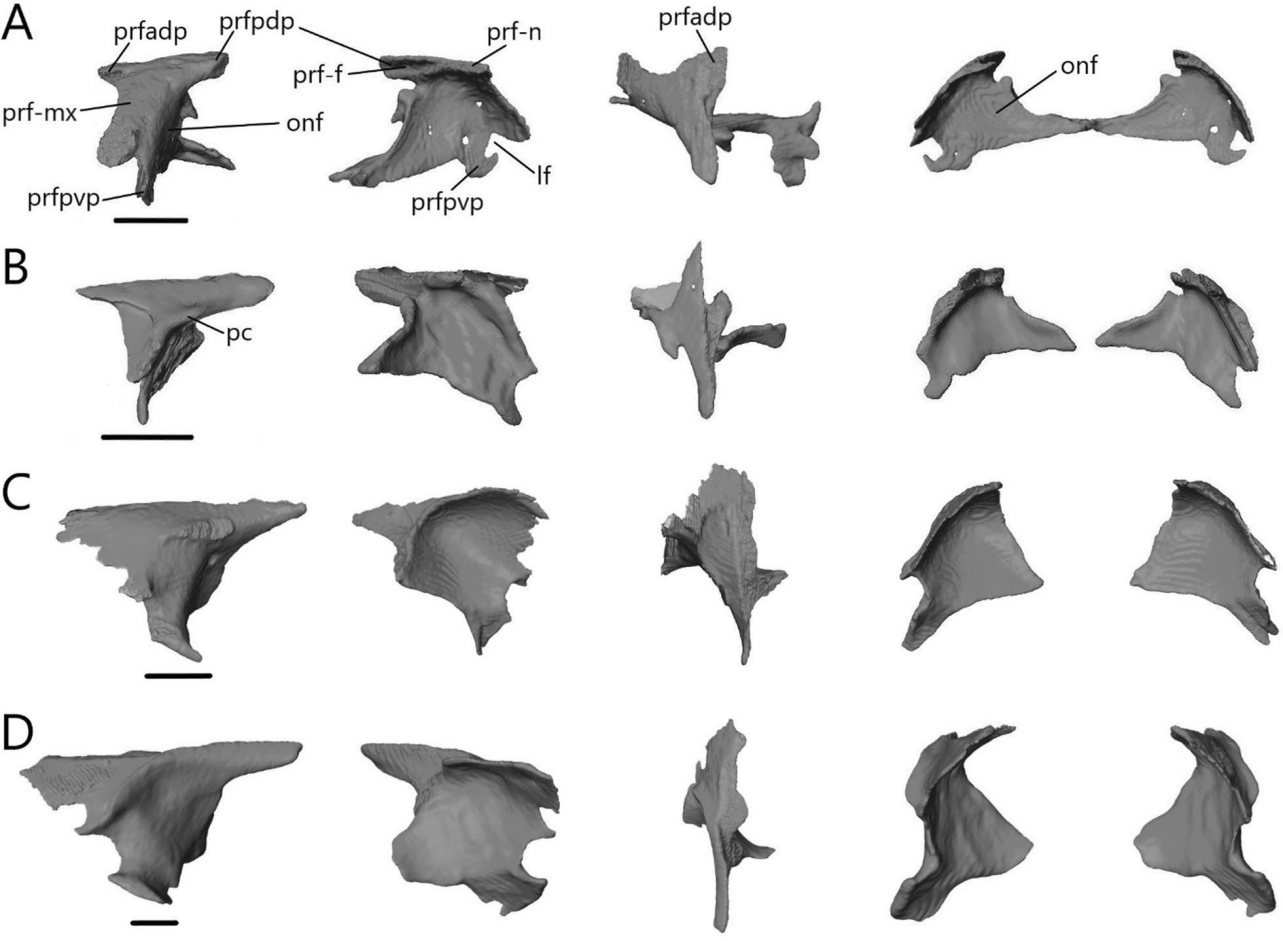
Isolated left prefrontal of **A** *T. brevipes*; **B** *T. gracilis*; **C** *S. alberti*; **D** *A. occidentalis*; in lateral, anteromedial, dorsal, and anterior views from left to right. lf, lacrimal foramen; onf, orbitonasal flange projection; pc, palpebral crest; prf-f, frontal facet of the prefrontal; prf-mx, maxillary facet of the prefrontal; prf-n, nasal facet of the prefrontal; prfadp, anterodorsal process of the prefrontal; prfpdp, posterodorsal process of the prefrontal; prfpvp, posteroventral process of the prefrontal. Scale bars = 0.5 mm

The prefrontal (Fig. 11) is a curved, paired bone that forms the anterior wall of the orbit. It contacts the maxilla anterolaterally, nasal dorsally, frontal dorsally and medially, and lacrimal laterally. There is additional ventromedial contact with the frontal in *T. brevipes*, *S. alberti*, and *A. occidentalis* and posterior contact with the postfrontal in both *Typhlacontias*.

The prefrontal has two dorsal processes in *Typhlacontias* (Fig. 11A, B). The anterodorsal process is the shorter of the two, ends in a triangular point, and contacts the nasal medially. The posterodorsal process has a rounded end, which contacts the postfrontal and frontal. Both are shorter in *T. brevipes*. The anterolateral surface, which is wider in *T. gracilis*, is overlapped by the dorsal process of the maxilla. The maxilla extends further dorsally in *T. brevipes*. Ventrally, this region forms the dorsal margin of the lacrimal foramen and ends in a rounded process in *T. brevipes* and a triangular point in *T. gracilis*. The posteroventral process forms the medial margin of the lacrimal foramen. It is small in *T. gracilis*, only curving ventrally, whereas it curves back up in *T. brevipes*. The palpebral crest is nearly indistinct in *T. brevipes* whereas in *T. gracilis*, it is a notable feature on the lateral side. The prefrontal curves medially to form the orbitonasal flange, which contacts the frontal dorsally. In anterior view, the paired prefrontals are shaped like bat wings with the orbitonasal flanges sloping down to the center. They meet in *T. brevipes*, joined by irregular interlocking projections, but stop short in *T. gracilis*. The medial end in *T. gracilis* is simpler, only a single broad knob. The tube formed by the orbitonasal flanges is continuous with the cristae cranii of the frontals.

The prefrontal of *S. alberti* (Fig. 11C) is more similar to *T. gracilis* than to *T. brevipes*. The anterodorsal process is indistinct. The posterodorsal process is triangular and contacts the frontal but not the postfrontal. The irregular edges of the anterolateral surface are likely artifacts of digital segmentation. Ventrally, the prefrontal flares out and forms the dorsal and medial margins of the lacrimal foramen. The broad palpebral crest is located on the lateral side and is where the palpebral contacts the prefrontal. The orbitonasal flange is triangular and smaller than in *Typhlacontias*, with a large gap between them. It contacts the frontal along its entire medial edge.

The anterodorsal process is shorter and wider than the posterodorsal process in *A. occidentalis* (Fig. 11D). Its dorsal surface curves medially, more so than in the other species, and underlies the nasal and frontal. The posterodorsal process maintains a nearly constant width. It makes up more than half of the dorsal wall of the orbit and contacts the frontal medially. The entire anterolateral surface is overlapped by the maxilla. The prefrontal forms the dorsal, medial, and ventral margins of the lacrimal foramen. The ventral margin is closed by the posteroventral process, which is larger than in the other species. The palpebral crest is well-defined and forms a ridge parallel to the maxilla. The orbitonasal flange interlocks with the frontal; it is posterolateral to the frontal dorsally but folds and becomes anterior to the frontal ventrally. Compared to the others, *A. occidentalis* has the smallest orbitonasal flange and the largest gap between the two prefrontals. Its orbitonasal flange also terminates in a blunt end rather than tapering to a point.

##### Palpebral

**Fig. 12:**
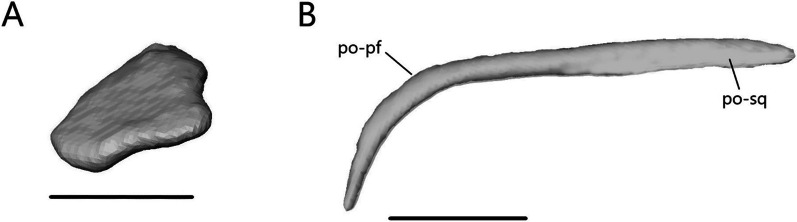
Isolated **A** left palpebral and **B** left postorbital of *S. alberti* in lateral view. po-pf, postfrontal facet of the postorbital; po-sq, squamosal facet of the postorbital. Scale bars = 0.5 mm

*Sepsina alberti* is the only one to have palpebrals (Fig. 12A). The palpebral is a paired bone that contacts the prefrontal anteromedially and lies in the anterior region of the orbit. It is flattened and roughly triangular with a slight medial concavity.

##### Postorbital

The postorbital is absent or fused to the postfrontal in *Typhlacontias* and *A. occidentalis*. Without developmental data, the distinction cannot be made. It is present as a separate element in *S. alberti* (Fig. 12B). The postorbital contacts the postfrontal anterodorsally along half its length and the squamosal posteriorly. It is a slender bone that curves anteroventrally, extending further ventrally than the postfrontal.

##### Postfrontal

**Fig. 13:**
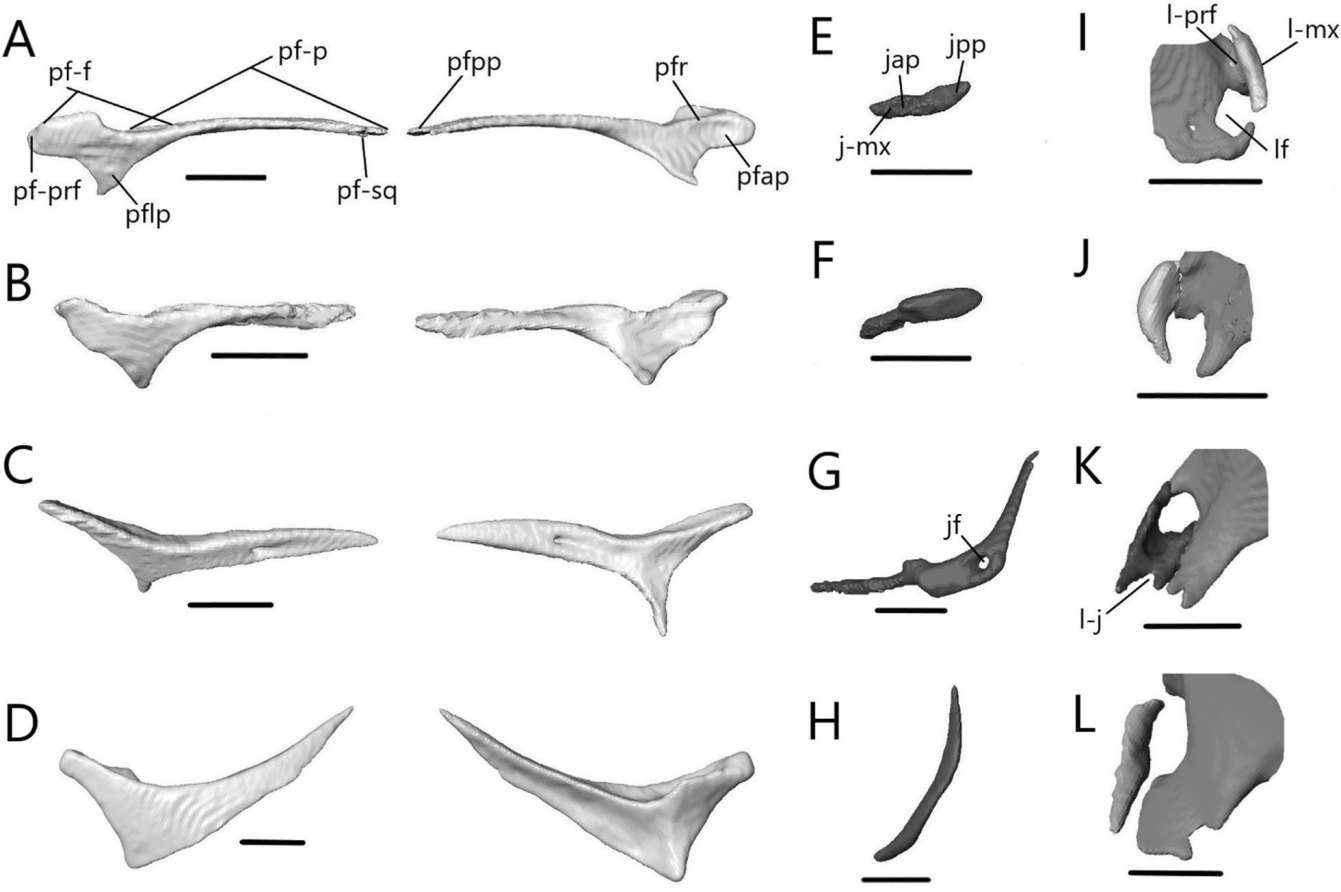
Isolated postfrontal, jugal, and lacrimal with prefrontal. Left postfrontal of **A** *T. brevipes*; **B** *T. gracilis*; **C** *S. alberti*; **D** *A. occidentalis*; in dorsal (left) and ventral (right) views Left jugal of **E** *T. brevipes*; **F** *T. gracilis*; **G** *S. alberti*; **H** *A. occidentalis*; in lateral view. **I** right lacrimal of *T. brevipes*; left lacrimal of **J** *T. gracilis*; **K** *S. alberti*; **L** *A. occidentalis*; in posterior view with prefrontal. j-mx, maxillary facet of the jugal; jap, anterior process of the jugal; jf, jugal foramen; jpp, posterior process of the jugal; l-j, jugal facet of the lacrimal; l-mx, maxillary facet of the lacrimal; l-prf, prefrontal facet of the lacrimal; lf, lacrimal foramen; pf-f, frontal facet of the postfrontal; pf-p, parietal facet of the postfrontal; pf-prf, prefrontal facet of the postfrontal; pf-sq, squamosal facet of the postfrontal; pfap, anterior process of the postfrontal; pflp, lateral process of the postfrontal; pfpp, posterior process of the postfrontal; pfr, ventral ridge of the postfrontal; Scale bars = 0.5 mm

The postfrontal (or possibly postorbitofrontal in *Typhlacontias* and *A. occidentalis*; Fig. 13A–D) is a triradiate paired bone that clasps the frontoparietal suture. It contacts the frontal anteromedially and the parietal posteromedially. It also contacts the prefrontal anteriorly in *Typhlacontias* and the postorbital ventrally in *S. alberti*.

The anterior process is short and broad, and contacts the prefrontal anteriorly in *Typhlacontias* (Fig. 13A, B). There is a ventral ridge that runs along the ventromedial side of the anterior process. It is more clearly defined in *T. brevipes*. The lateral process is shorter than the anterior process, curves ventrolaterally, and is triangular. The posterior process is longer and narrower than the other two processes and is pressed close to the parietal. It tapers to a point in *T. brevipes* and to a broad knob in *T. gracilis*. There is a triangular facet that articulates with the squamosal on its lateral side.

The anterior process is slender and long in *S. alberti* (Fig. 13C). A well-defined ventral ridge runs alongside the ventromedial side. The lateral process is the longest of the four species relative to skull size. It is triangular and narrows to a fine point. The posterior process tapers to a rounded end. A small foramen is present halfway on the posterior process.

The postfrontal of *A. occidentalis* is shaped like a checkmark and follows the curvature of the parietal to the point of constriction (Fig. 13D). The anterior process is shorter and broader than the posterior process, but narrower than in *Typhlacontias*. The ventral ridge runs along the entire anterior process and down the posterior process. The lateral process is broad and triangular. The posterior process is shorter than in the scincines and folds medially, ending in a sharp point.

##### Jugal

The jugal (Fig. 13E–H) is a paired, elongate, and curved bone. It does not contact any bone in *A. occidentalis*, but in the others, it contacts the maxilla ventrolaterally. It also contacts the ectopterygoid ventromedially in *T. gracilis* and the lacrimal anteriorly in *S. alberti*.

The jugal is highly reduced in *Typhlacontias*. The anterior process is about the same size as the posterior process in *T. brevipes* (Fig. 13E), and smaller in *T. gracilis* (Fig. 13F). The jugal has a slight bend where the two processes meet and some tapering near the anterior end in *T. brevipes*. In *T. gracilis*, the jugal is constricted where the two processes meet and the anterior process curves medially to follow the lateral border of the ectopterygoid. Both processes are pointed in *T. brevipes* and rounded in *T. gracilis*.

Of the species examined, *S. alberti* is the only one without a greatly reduced jugal (Fig. 13G). The anterior process is narrow and extends along the posterior process of the maxilla to the lacrimal. There is a small dorsal bump where the anterior process joins the rest of the jugal, after which the jugal widens. The posterior process curves dorsally, narrowing to a point. A jugal foramen is present where the jugal begins to curve dorsally.

The jugal is reduced in *A. occidentalis* (Fig. 13H), but rather than reducing the posterior process, *A. occidentalis* has reduced the anterior process. It is a small splint that borders the posterior side of the orbit. It is somewhat flattened and tapered at both ends.

##### Lacrimal

The lacrimal (Fig. 13I–L) is a small, paired bone that contacts the prefrontal medially and the maxilla laterally. It also contacts the maxilla ventrally and the jugal posteriorly in *S. alberti*. Within an individual, contralateral lacrimals often differ in size.

The lacrimal forms the lateral border of the lacrimal foramen in *Typhlacontias* (Fig. 13I, J). The size difference between the two sides is obvious in *T. brevipes* where the left lacrimal is smaller and closes less than half the lateral margin of the lacrimal foramen. The comma-shaped lacrimal is larger and the two sides equal in size in *T. gracilis*.

The lacrimal of *S. alberti* (Fig. 13K) is more complex than in the other species. Anteriorly, it forms the ventral and lateral margins of the lacrimal foramen. The bone forms an open U-shaped tube with the lateral side extending further dorsally than the medial side. It contacts the prefrontal on both sides and the maxilla on the lateral side. The lacrimal slopes posteroventrally, flattening, and then splits into two posterior processes. The lateral one is longer. Between these two processes, there is a V-shaped indent, into which the jugal fits.

The lacrimal rests along the medial wall of the maxilla for most of its length in *A. occidentalis* (Fig. 13L), a more reclined position compared to the more vertically oriented lacrimal of *Typhlacontias*. At its anterior end, it borders the lacrimal foramen ventromedially. Its posterior half runs parallel to the prefrontal without contacting it. The lacrimal tapers posteriorly.

##### Frontal

**Fig. 14:**
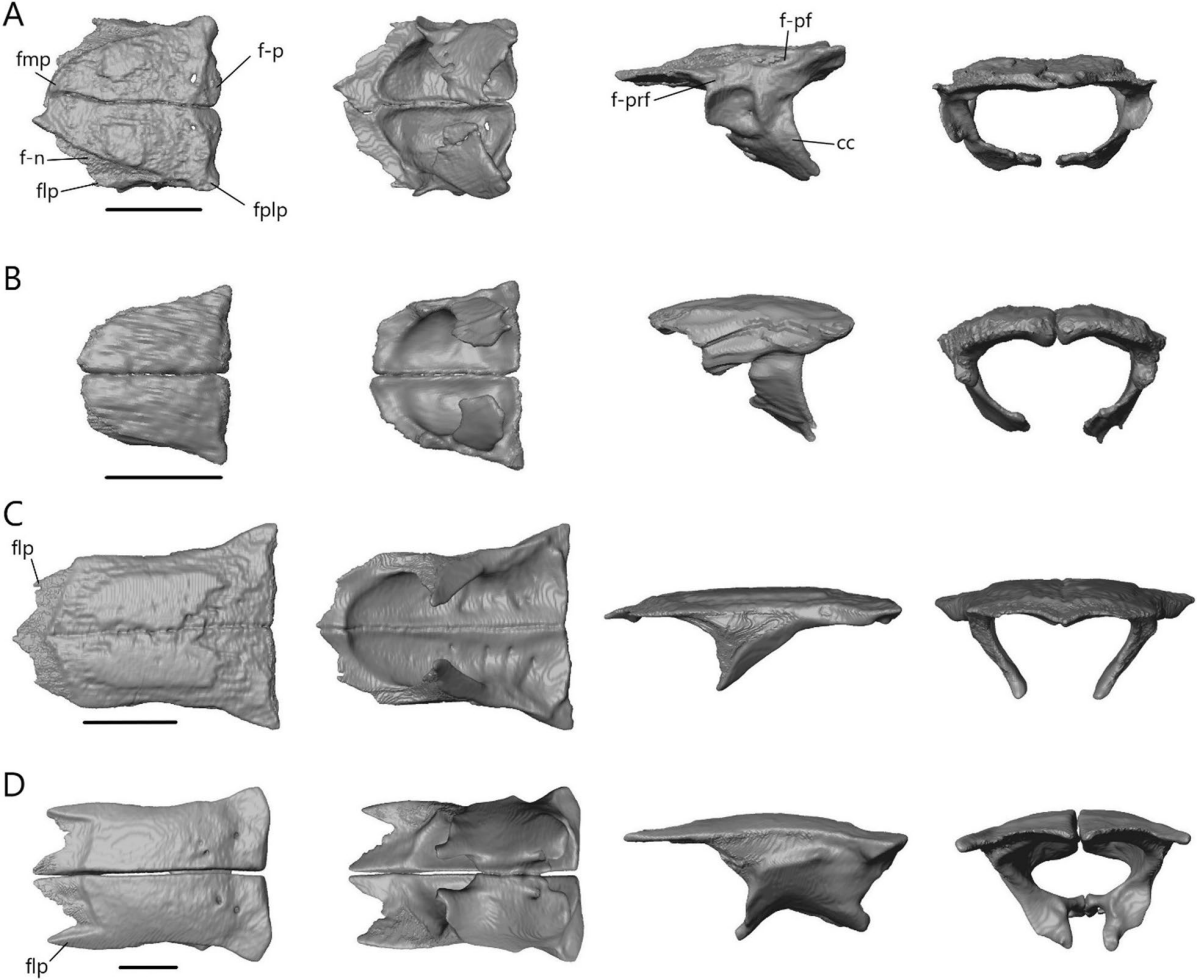
Isolated frontals of **A** *T. brevipes*; **B** *T. gracilis*; **C** *S. alberti*; **D** *A. occidentalis*; in dorsal, ventral, lateral, and anterior views from left to right. cc, crista cranii; f-n, nasal facet of the frontal; f-p, parietal facet of the frontal; f-pf, postfrontal facet of the frontal; f-prf, prefrontal facet of the frontal; flp, lateral process of the frontal; fmp, medial process of the frontal; fplp, posterolateral process of the frontal. Scale bars = 1 mm

The frontal (Fig. 14) is paired with medial contact with its mate. It contacts the nasals anteriorly, prefrontal laterally, postfrontal posterolaterally, and parietal posteriorly. There is additional contact with the prefrontal ventrally in *T. brevipes*, *S. alberti*, and *A. occidentalis*. It also contacts the maxilla anterolaterally in *A. occidentalis*. The frontal has a flat dorsal surface, which forms part of the skull roof, and a ventral descending flange called the crista cranii. The two cristae cranii form a canal for the olfactory tracts.

The frontal of *Typhlacontias* is trapezoidal with a broad posterior border that narrows anteriorly (Fig. 14A, B). The medial process is U-shaped in both species. The frontal of *T. brevipes* has a small, pointed lateral process whereas *T. gracilis* lacks this. Between the medial and lateral processes of *T. brevipes*, there is a depressed facet, which is overlapped by the nasal. The posterior border is slightly curved. The parietal overlaps the frontal at the posteromedial shelf, which is larger and more pronounced in *T. brevipes*. The posterolateral process is rounded and overlaps the parietal. The cristae cranii curve ventromedially but do not meet, although they come closer in *T. brevipes*. In anterior view, the tube formed by the cristae cranii is flatter in *T. brevipes* and more rounded in *T. gracilis*. The cristae cranii are more robust in *T. brevipes* and have large facets anteriorly. They are narrower and lack facets in *T. gracilis*.

The frontal of *S. alberti* is subrectangular, and only slightly narrowed anteriorly (Fig. 14C). The anterior side forms a large shelf, upon which the nasals sit. This gives the frontal an obtuse, triangular shape when viewed in the cranium (Fig. 6B). The medial process is triangular in shape. The lateral process is also triangular although it does not extend as far anteriorly. The parietal overlaps the frontal medially but there is no distinct posteromedial shelf, only a slight depression. The posterolateral process is triangular and overlaps the parietal as in *Typhlacontias*. The cristae cranii are triangular and oriented anteromedially. They do not show as much curvature as in the others and do not enter the medial region between the orbits. The anterior side of the crista cranii contacts the entire medial length of the prefrontal, closing the anterior orbit wall.

The frontal of *A. occidentalis* is rectangular (Fig. 14D). The medial processes form a triangle. The lateral process is large and triangular, extending about as far anteriorly as the medial process. Between them, there is a large depressed shelf upon which the nasal sits. The medial process is covered by the nasal and the lateral process is bordered by the nasals medially and the maxillae laterally. *Acontias occidentalis* lacks posteromedial shelves; the parietal and frontal meet at an angle along the posteromedial side with the parietal slightly overlapping the frontal. The posterolateral process overlaps the parietal. The cristae cranii are much larger in *A. occidentalis*. They meet at the midline, creating an enclosed tube for the olfactory tracts. Anteriorly, they contact the prefrontal, further reinforcing the skull. Medial processes from the cristae cranii curve ventrally, extending to an area between the vomer and palatine without contacting either. Two large foramina are present anterior to the posterior ends of the cristae cranii.

##### Parietal

**Fig. 15:**
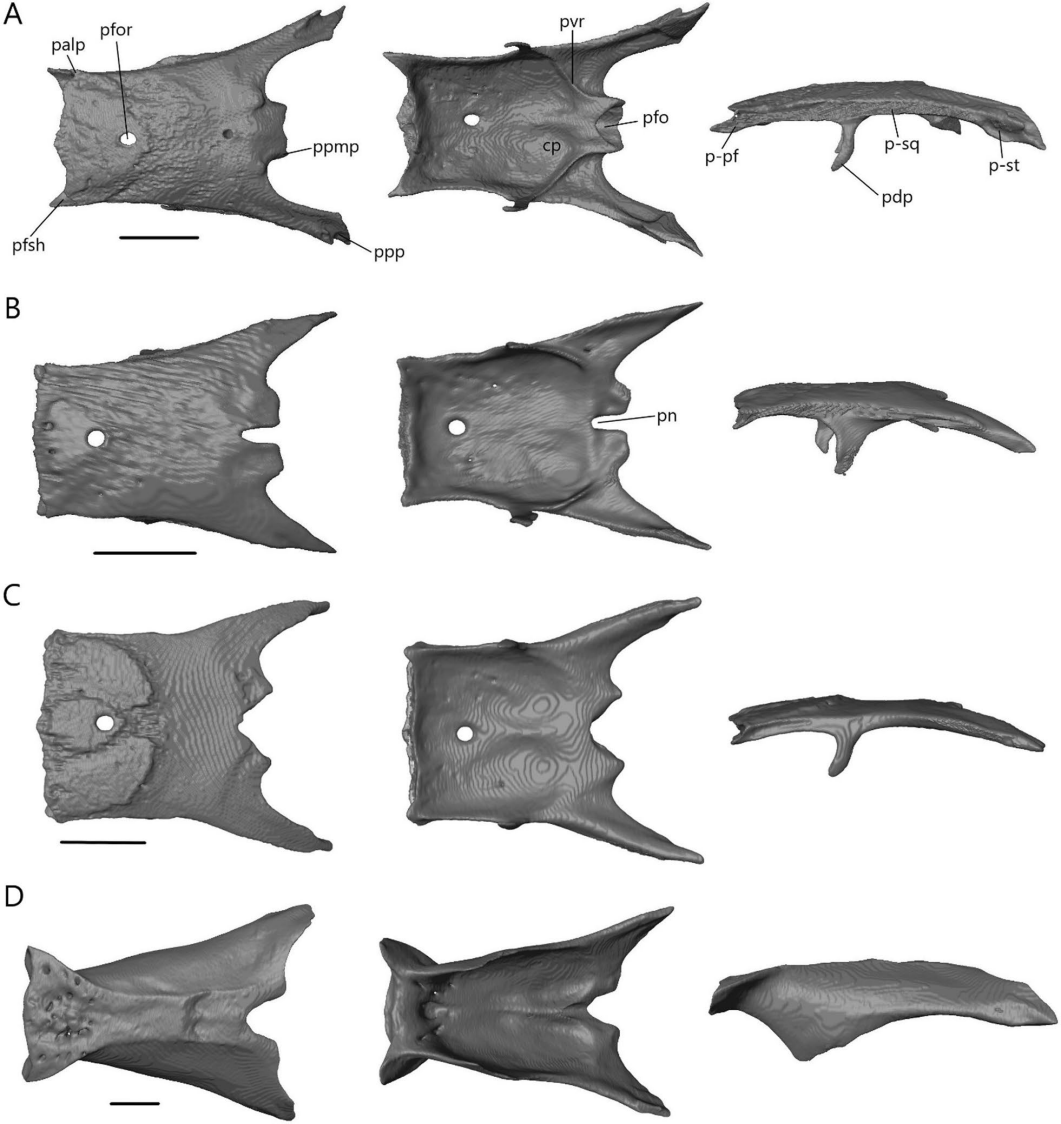
Isolated parietal of **A** *T. brevipes*; **B** *T. gracilis*; **C** *S. alberti*; **D** *A. occidentalis*; in dorsal, ventral, and lateral views from left to right. cp, concavity of the parietal; p-pf, postfrontal facet of the parietal; p-st, supratemporal facet of the parietal; p-sq, squamosal facet of the parietal; palp, anterolateral process of the parietal; pdp, descending process of the parietal; pfo, parietal fossa; pfor, parietal foramen; pfsh, parietal shelf for the frontal; pn, parietal notch; ppmp, posteromedial process of the parietal; ppp, posterior process of the parietal; pvr, ventral ridge of the parietal. Scale bars = 1 mm

The parietal (Fig. 15) is a large, fused bone that contacts the frontal anteriorly, postfrontal anterolaterally, and prootic posteroventrally. It also contacts the squamosal and supratemporal posterolaterally in the scincines. The parietal is composed of a large table, which forms the posterior portion of the skull roof, and two descending processes. On the ventral side, there is a concavity that accommodates the cerebrum, optic lobe, and cerebellum.

The parietal is a subrectangular bone in *Typhlacontias*. Its anterior border is slightly W-shaped in *T. brevipes* (Fig. 15A) and straight in *T. gracilis* (Fig. 15B). It has two small, triangular shelves at the sides, which are overlapped by the frontal. Small anterolateral processes form the postfrontal facet. A parietal foramen is present. The parietal has two elongate posterior processes and two squat posteromedial processes. The posteromedial processes of *T. brevipes* are almost entirely fused, with only a small divot between them, to form the dorsal wall of the parietal fossa. The parietal fossa is a large ovoid opening. It narrows anteriorly until it reaches a small foramen on the dorsal surface. The cartilaginous processus ascendens likely lies inside this tube as documented in *Ablepharus* [52]. *Typhlacontias gracilis* has a small parietal fossa visible posteriorly (Fig. 5E) but lacks the dorsal foramen. The rectangular posteromedial processes of *T. gracilis* are clearly separate with a deep parietal notch between them. The long posterior processes contact the squamosal and supratemporal laterally, and the prootic ventromedially. Contact with the squamosal extends anteriorly past the posterior process. The ends of the posterior processes in *T. brevipes* have a slight bifurcation and a concave surface whereas the ends in *T. gracilis* taper to single points and are flat. Despite these differences in shape, the relative length is roughly 36% of the entire parietal in both species. The descending process is widest at the base and narrows ventrally to its tip. It is wider in *T. gracilis* than *T. brevipes*. In *T. gracilis*, it extends past the head of the epipterygoid and contacts its medial surface (Fig. 5A). Due to the uneven size between the two epipterygoids in *T. brevipes*, only the right descending process extends past the epipterygoid although there is no contact. Ventral ridges run from the descending process to the parietal fossa in *T. brevipes*. They are present, but terminate more anteriorly in *T. gracilis*.

Although relatively wider, the parietal of *S. alberti* (Fig. 15C) is similar in shape to that of *Typhlacontias*, especially *T. gracilis*. The anterior edge is nearly straight except for a small medial curve which overlaps the frontals. The frontals overlap two small, triangular shelves located laterally. The postfrontal facet extends from the lateral ridge formed by these shelves to the descending process. The posteromedial processes are triangular and form a wide V-shaped parietal notch between them. As in *T. gracilis*, there is a small parietal fossa there (Fig. 6E). The long posterior processes contact the squamosal and supratemporal laterally, and the prootic ventromedially. Contact with the squamosal does not continue anteriorly and so the upper temporal fenestrae remain open. The posterior processes retain the same width for most of their length, sharply constricting near the end. They account for roughly 40% of the total parietal length. The descending process is very similar to that in *T. brevipes*, although not as long. It extends past the head of the epipterygoid but does not contact it.

The parietal of *A. occidentalis* (Fig. 15D) is constricted near its anterior margin, creating an overall hourglass shape quite different from the other species. The horizontal parietal table is smaller in *A. occidentalis* as it curves downward. The anterior margin is slightly curved with small lateral shelves which the frontal overlaps. The parietal foramen is not patent, but there is an indentation on the ventral surface marking its position. The posteromedial processes are small tabs on either side of the U-shaped parietal notch. Similar to *T. gracilis* and *S. alberti*, this notch has a small parietal fossa at its center and there is no dorsal foramen; however, unlike in those two species, ventral ridges run along both sides of the fossa. On the dorsal surface, there is a ridge and posterior concavity here. The posterior processes are large and triangular, tapering to a single point. Their vertical expansion contributes to the closure of the braincase. They are only slightly longer (39%) than in *Typhlacontias*. The descending process has been greatly expanded and extends from the anterior margin to the posterior process, effectively closing half the skull. It is triangular and extends past the tip of the epipterygoid without contacting it.

##### Interparietal

The interparietal (inp, Fig. 6B) is only present in *S. alberti*. It is a small, paired bone which contacts the supraoccipital posteriorly. The right interparietal, which is larger, also contacts the parietal anterolaterally. It is triangular and curved on its ventral side.

##### Squamosal

**Fig. 16:**
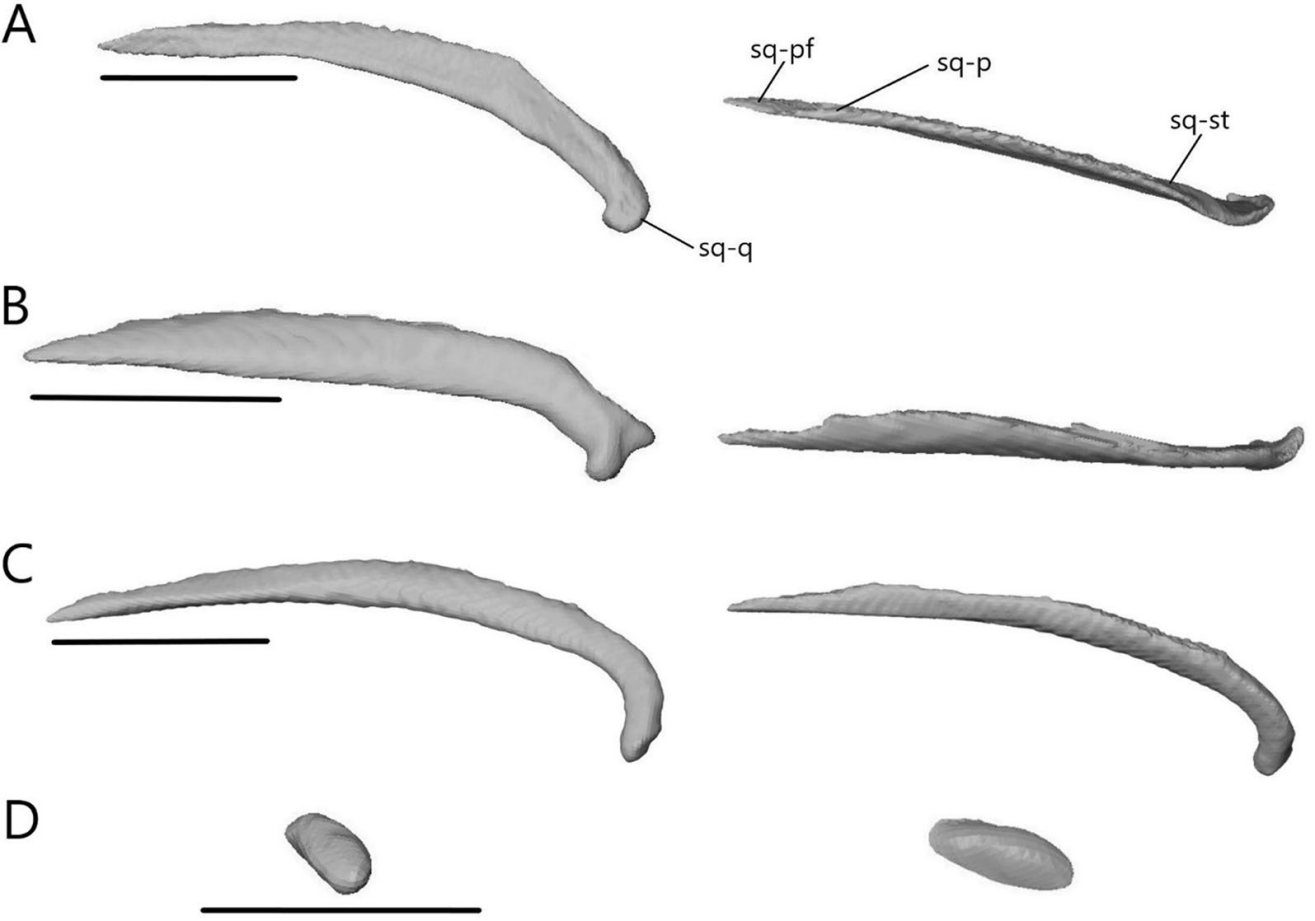
Isolated left squamosal of **A** *T. brevipes*; **B** *T. gracilis*; **C** *S. alberti*; **D** *A. occidentalis*; in lateral (left) and dorsal (right) views. sq-pf, postfrontal facet of the squamosal; sq-p, parietal facet of the squamosal; sq-q, quadrate facet of the squamosal; sq-st, supratemporal facet of the squamosal. Scale bars = 1 mm

Except in *A. occidentalis*, where it is highly reduced, the squamosal (Fig. 16) is an elongate, paired bone that curves posteriorly and tapers to a point anteriorly. It contacts the parietal medially, supratemporal posteromedially, and quadrate posteroventrally. It contacts the postfrontal anteromedially in *Typhlacontias* and the postorbital anteromedially in *S. alberti*.

The squamosal contacts the parietal for most of its length, closing the upper temporal fenestra in *Typhlacontias* (Fig. 5B). It is flattened except where it widens posteriorly near where it articulates with the quadrate. There is a single condyle in *T. brevipes* (Fig. 16A), but in *T. gracilis*, there is both a ventral condyle which fits into the squamosal notch and a posterior condyle in contact with the supratemporal (Fig. 16B). The squamosal of *T. gracilis* is relatively longer and wider and shows more curvature than in *T. brevipes*.

Overall, the squamosal of *S. alberti* (Fig. 16C) is more slender than in *Typhlacontias*. It contacts the parietal at its posterior region and forms the lateral border of the upper temporal fenestra. There is a single condyle at the posterior end, which articulates with the quadrate.

The squamosal of *A. occidentalis* (Fig. 16D) has been greatly reduced to an ovoid stub located near the supratemporal. It does not directly contact any bone.

##### Septomaxilla

**Fig. 17:**
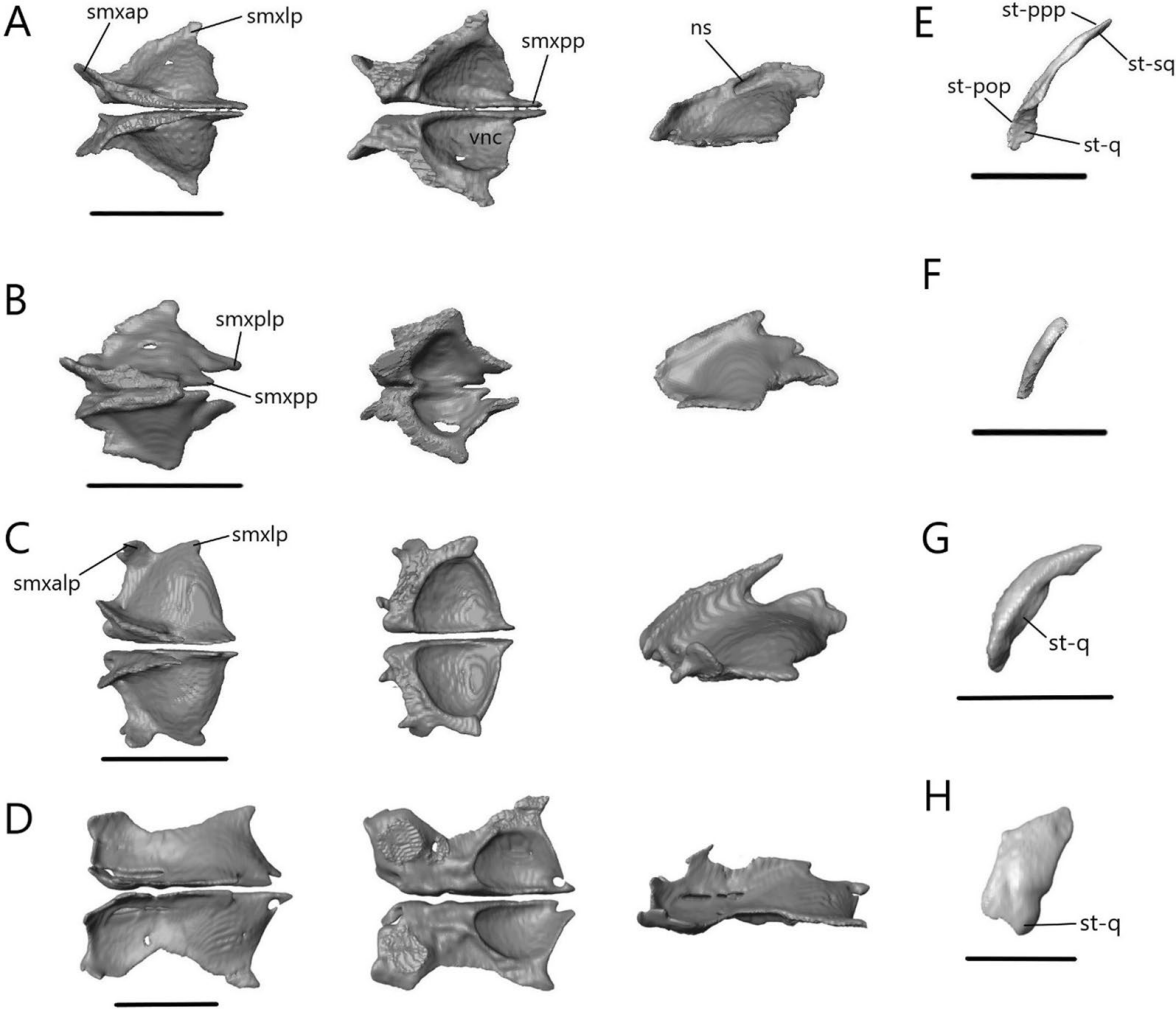
Isolated septomaxillae and supratemporal. Septomaxillae of **A** *T. brevipes*; **B** *T. gracilis*; **C** *S. alberti*; **D** *A. occidentalis*; in dorsal, ventral, and lateral views from left to right. Right supratemporal of **E** *T. brevipes*; **F** *T. gracilis*; **G** *S. alberti*; **H** *A. occidentalis*; in lateral view. ns, nasal septum; smxalp, anterolateral process of the septomaxilla; smxap, anterior process of the septomaxilla; smxlp, lateral process of the septomaxilla; smxplp, posterolateral process of the septomaxilla; smxpp, posterior process of the septomaxilla; st-pop, paroccipital process facet of the supratemporal; st-ppp, posterior process of the parietal facet of the supratemporal; st-q, quadrate facet of the supratemporal; st-sq, squamosal facet of the supratemporal; vnc, ventral concavity for vomeronasal apparatus. Scale bars = 1 mm

The septomaxilla (Fig. 17A–D) is a paired bone that contacts its counterpart medially, nasals dorsally and maxilla ventrally. It also contacts the premaxilla anteroventrally in *T. gracilis* and *A. occidentalis*, and vomer posteroventrally in *T. gracilis*. It is positioned above the vomer on the floor of the nasal cavity and covers the vomeronasal apparatus (Jacobson’s organ).

The ventral concavity occupied by the vomeronasal apparatus is relatively larger in *T. brevipes* (Fig. 17A) than in *T. gracilis* (Fig. 17B). The dorsal surface is domed and curves vertically to form the nasal septum. In *T. gracilis*, the two septomaxillae are joined here; they remain separate in *T. brevipes*. The anterior process projects anterolaterally from the body. The lateral process projects posterolaterally and forms the widest point. It is triangular in *T. brevipes* and rounded in *T. gracilis*. In *T. brevipes*, the posterior side narrows to the center-line where the posterior process projects from. The posterior side is curved in *T. gracilis* and first forms the posterolateral process, which contacts the vomer ventrally. Medial to it, there is a smaller posterior process.

The ventral concavity makes up most of the septomaxilla’s length in *S. alberti* (Fig. 17C). The dorsal surface is domed and forms a vertical nasal septum stretching from the anterior margin past the center of the bone. The nasal septum tapers to a fine point posteriorly. The anterior process projects anterolaterally, although not as much as in *T. brevipes*, and the small, triangular lateral process projects posterolaterally. Midway between these, the anterolateral process projects anterolaterally with a slight dorsal curve. It forms a large, rounded knob. From the lateral process, the septomaxilla turns medially. A small, triangular posterior process project out at the posterior margin.

Unlike in *Typhlacontias* and *Sepsina*, the ventral concavity only makes up about half the length of the septomaxilla in *A. occidentalis* (Fig. 17D). Anteriorly, the septomaxilla has a broad edge, which curls upwards laterally. The triangular lateral process projects posterolaterally. The septomaxilla then curves medially to form the triangular posterior process. There is a large foramen at the base of the posterior process. The dorsal surface curves to form a small nasal septum.

##### Supratemporal

The supratemporal (Fig. 17E–H) is a small, paired bone that contacts the parietal anteromedially, squamosal anterolaterally, paroccipital process posteromedially, and quadrate posterolaterally in the scincines. In *A. occidentalis*, it contacts the paroccipital process posteromedially and quadrate ventrally.

The contact between the supratemporal and squamosal is more extensive in *T. gracilis* (Fig. 17F), continuing for most of the supratemporal’s length, whereas the squamosal only contacts the anterior end of the supratemporal in *T. brevipes* (Fig. 17E). The supratemporal of *T. gracilis* is shorter and wider with only a slight flattening at the quadrate facet. In *T. brevipes*, the supratemporal is longer, with a flattened lateral quadrate facet. There is a small ridge anterior to the quadrate facet in *T. brevipes*.

The supratemporal of *S. alberti* (Fig. 17G) is more robust than in *Typhlacontias*. It is curved with a pointed anterior end and a rounded posterior end. It has a large indentation near the quadrate articulation. The contact with the squamosal is limited to the anterior half of the supratemporal. Medially, it is in continuous contact with the parietal and the paraoccipital.

The supratemporal of *A. occidentalis* (Fig. 17H) is much broader than in the other species. It is trapezoidal and flattened. The rounded ventral end articulates with the cephalic condyle of the quadrate. There is a slight depression anteriorly, similar to the squamosal facet seen in the others, though it does not contact the squamosal.

##### Palatine

**Fig. 18:**
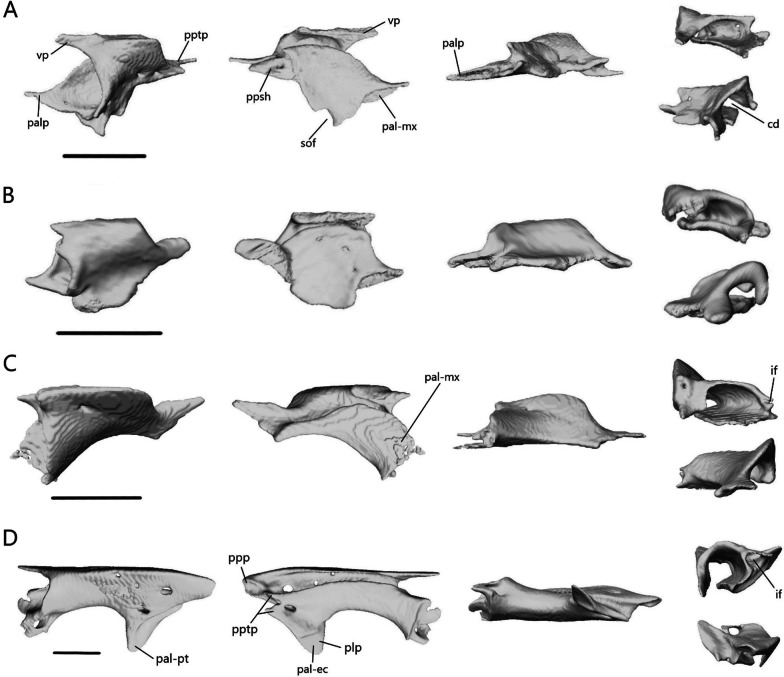
Isolated left palatine of **A** *T. brevipes*; **B** *T. gracilis*; **C** *S. alberti*; **D** *A. occidentalis*; in dorsal, ventral, lateral, anterior (top), and posterior (bottom) views from left to right. cd, choanal duct; if, infraorbital foramen; pal-ec, ectopterygoid facet of the palatine; pal-mx, maxillary facet of the palatine; pal-pt, pterygoid facet of the palatine; palp, anterolateral process of the palatine; plp, lateral process of the palatine; ppp, posterior process of the palatine; ppsh, pterygoid process shelf; pptp, pterygoid process of the palatine; sof, suborbital fenestra; vp, vomerine process. Scale bars = 1 mm

The palatine (Fig. 18) is a paired bone that contacts the vomer anteromedially, maxilla anterolaterally, and pterygoid posteriorly. It also contacts the ectopterygoid ventrolaterally in *A. occidentalis*. The palatines almost contact one another medially, but there remains a narrow gap in all four species. Each palatine consists of a flat ventral lamina and curved dorsal flange, which meet laterally. The choanal duct runs between them.

The ventral surface is flat and varies in shape between the two *Typhlacontias*. In *T. brevipes*, the venter is more gracile and subtriangular (Fig. 18A) whereas in *T. gracilis*, it is subrectangular (Fig. 18B). The medial margin curves anterolaterally to form the slender anterolateral process. Lateral to this process, there is a small shelf facet which articulates with the medial shelf of the maxilla. The anterolateral process of *T. brevipes* is longer than that of *T. gracilis*. The lateral margin forms the anteromedial border of the suborbital fenestra. They differ in shape, with *T. brevipes* having a semicircular notch and *T. gracilis* a straight edge. The pterygoid process extends posteriorly into the interpterygoid vacuity. It narrows into a slim bar in *T. brevipes* and remains broad in *T. gracilis*. The pterygoid process has a broad shelf which articulates with the palatine process of the pterygoid ventrally. From the lateral margin, a flange curves dorsomedially, coming near the center line of the skull and then folding ventrally to form the vomerine process. The vomerine process overlaps the vomer dorsally. It is longer in *T. brevipes* than in *T. gracilis*. The anterior opening of the choanal duct is elliptical and somewhat flattened. The posterior opening is more circular.

The ventral surface is narrower and there is a larger gap between the two palatines in *S. alberti* (Fig. 18C), leaving more of the palate open than in *Typhlacontias*. The venter forms a crescent shape with a broad anterior end that tapers to a pointed posterior end. The maxilla covers a semioval region of the anterior end. Laterally, the anterior margin curves dorsally to form a small infraorbital fenestra. There are no distinct processes at the anterior border of the venter. The pterygoid process, which extends from the dorsal flange of the palatine and connects with the venter, is broad and triangular. It overlaps the pterygoid. The palatine forms the medial border of the suborbital fenestra. The dorsal flange is relatively longer in *S. alberti* than in *Typhlacontias* and has a small foramen near the middle. Anteriorly, it forms the triangular vomerine process. The anterior opening of the choanal duct is larger than in *Typhlacontias* and oval in shape. The posterior opening, however, is much smaller and circular.

The ventral surface in *A. occidentalis* is narrower than in *Typhlacontias* and has an arched rectangular shape (Fig. 18D). The anterior margin is flat and bears a large, rounded shelf which contacts the maxilla. Directly lateral to the shelf, the palatine curls in on itself to form the large infraorbital foramen. The palatine forms the entire medial and posterior border of the suborbital fenestra. The posterolateral margin forms a half-elliptical, dorsally oriented lateral process, which articulates with the ectopterygoid ventrally and the pterygoid dorsally. This process is not seen in the scincines. The pterygoid process is forked, with each part narrowing into a triangular point. The pterygoid fits between them and overlaps the lateral pterygoid process. There is a foramen near the base of the pterygoid process. The anterior opening of the choanal duct is much larger and more circular than in the other species. However, the posterior opening is smaller and more compressed. The vomerine process is narrower and sharper. The posterior end forms a broad posterior process, the ventral side of which contacts the pterygoid. It extends further posteriorly than the pterygoid process. There are two foramina on the dorsal surface, one near the lateral process and one posteromedial.

##### Vomer

**Fig. 19:**
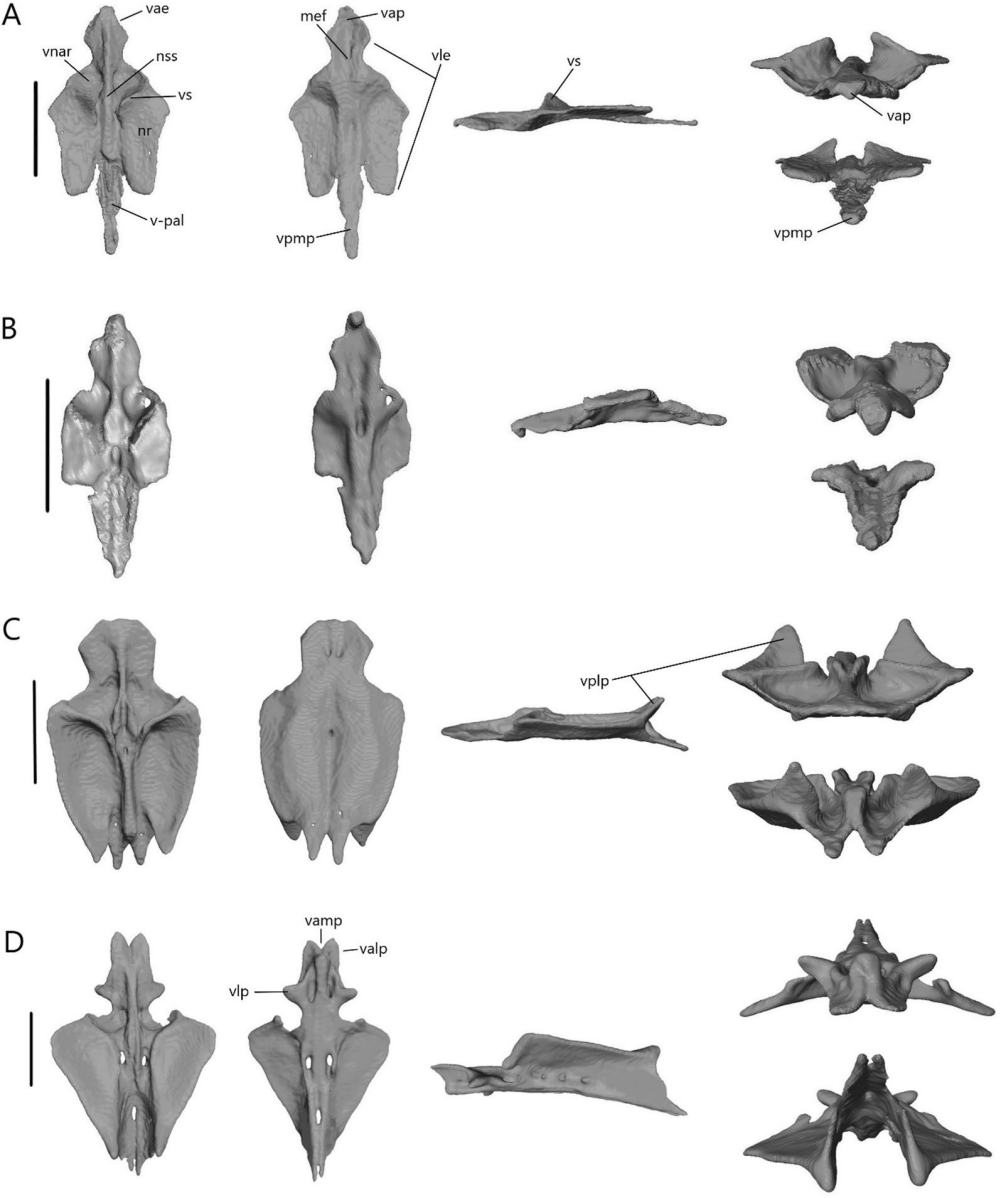
Isolated vomer of **A** *T. brevipes*; **B** *T. gracilis*; **C** *S. alberti*; **D** *A. occidentalis*; in dorsal, ventral, lateral, anterior (top), and posterior (bottom) views from left to right. mef, medial foramen of the vomer; nr, nasal region of the vomer; nss, space for the nasal septum; v-pal, palatine facet of the vomer; vap, anterior process of the vomer; vae, anterior edge of the vomer; valp, anterolateral process of the vomer; vamp, anteromedial process of the vomer; vle, lateral edge of the vomer; vlp, lateral process of the vomer; vnar, vomeronasal region of the vomer; vp, vomerine process; vplp, posterolateral process of the vomer; vpmp, posteromedial process of the vomer; vs, vomerine septum. Scale bars = 1 mm

The vomer (Fig. 19) is a fused bone that contacts the palatine posteriorly. It contacts the premaxilla anteriorly in *T. gracilis* and *A. occidentalis*, and the septomaxilla dorsally in *T. gracilis*. The lateral edge forms the medial border of the opening for the vomeronasal apparatus anteriorly and the medial border of the choana posteriorly.

The anterior process is rounded at the tip in *Typhlacontias*. In *T. gracilis*, it contacts the premaxilla anteriorly and has a ventral knob (Fig. 19B). A ventral groove runs from the anterior tip to a small medial foramen. The medial foramen is located further posteriorly in *T. gracilis* than in *T. brevipes* (Fig. 19A). The anterior margin runs parallel to the premaxilla. The lateral edge flares out more in *T. brevipes*, closing more of the palate, whereas in *T. gracilis*, the lateral edge is relatively straight. The dorsal surface is divided into the smaller vomeronasal region anteriorly and larger nasal region posteriorly by the vomerine septum, a ridge that runs from the edges laterally to the center and then curves posteriorly. The vomerine septum is larger and more pointed in *T. brevipes*, whereas in *T. gracilis*, the vomerine septum is rounded and protrudes less from the dorsal surface. The nasal region is larger in *T. brevipes*. Ventrally, this region is concave, and more pronounced in *T. gracilis*. The space for the nasal septum runs medially along the dorsal surface as a distinct ridge. It is interrupted in *T. gracilis* by the medial foramen. The posterior region differs between the two species. In *T. brevipes*, the vomer ends in broad lappets whereas in *T. gracilis*, the lateral edge folds straight into the center, completing its squarish appearance. The posteromedial process extends from the centerline to the posterior end of the palatines, further closing the palate. The palatines contact the vomer on the dorsal surface of this process. The posteromedial process is narrower in *T. brevipes*. It is broad and triangular in *T. gracilis*.

The vomer is wider throughout its entire length in *S. alberti* (Fig. 19C) than in *Typhlacontias* or *A. occidentalis*. The anterior process is broader. It is pentagonal with a wide anterior margin which does not contact the premaxilla. *Sepsina alberti* has two medial foramina on either side of the center line on the anterior process. The vomerine septum is more distinct in *S. alberti* than in *Typhlacontias*. The nasal region is concave, which gives the ventral surface a domed appearance. This is not seen in *Typhlacontias* or *A. occidentalis*, even though they also show concave nasal regions. The space for the nasal septum is distinct in *S. alberti* and has twin ridges anterior to the vomerine septum. There is a small foramen posterior to these ridges. The nasal region ends in dorsally slanted, triangular posterolateral processes. They overlap the palatines without contacting them. Two short and triangular posteromedial processes originate from either side of the center line and contact the vomerine process of the palatine dorsally.

The vomer of *A. occidentalis* is roughly diamond-shaped (Fig. 19D). Rather than a single anterior process, *A. occidentalis* has two rounded anterolateral processes, which contact the premaxilla ventrally, and a single anteromedial process, which protrudes ventrally. Two ventral grooves run along either side of this process, ending in two medial foramina. These foramina also have openings on the dorsal surface. The anterior margin borders but does not contact the premaxilla and maxilla. It terminates with the lateral processes, short but distinct knobs. The lateral edge flares out, extending past the maxilla at its widest point to close the entire roof of the mouth. The vomeronasal region has been reduced to a slim crescent and the nasal region is elevated. Three oval foramina are located along the space for the nasal septum, two anterior on either side and one posterior located centrally. The vomer tapers down to form the posteromedial processes. The posteromedial processes are much smaller than in *Typhlacontias* and only cover the most anterior region of the palatine.

##### Pterygoid

**Fig. 20:**
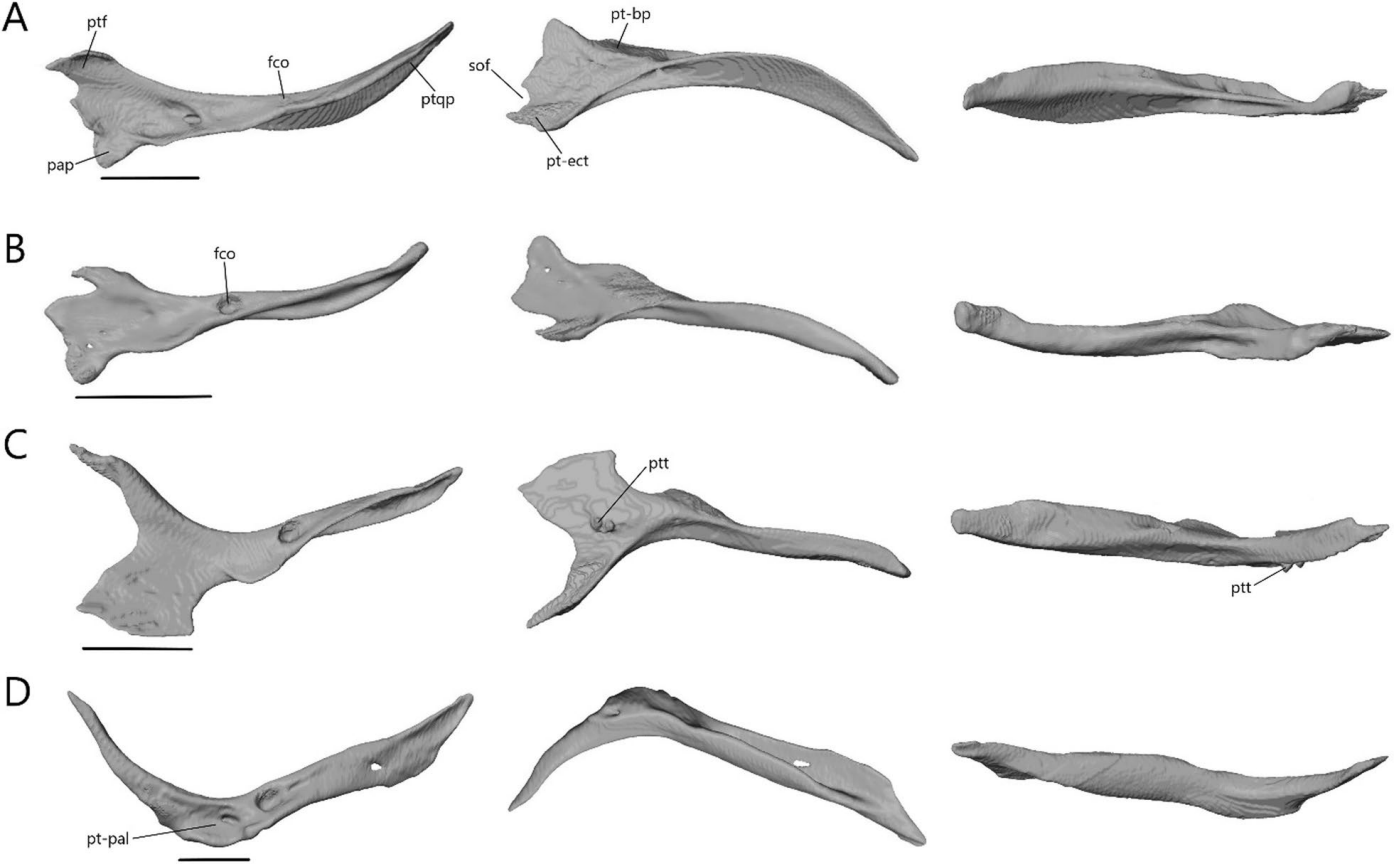
Isolated right pterygoid of **A** *T. brevipes*; **B** *T. gracilis*; **C** *S. alberti*; **D** *A. occidentalis*; in dorsal, ventral, and lateral views from left to right. fco, fossa columellae; pap, palatine process of the pterygoid; pt-bp, basipterygoid facet of the pterygoid; pt-ect, ectopterygoid facet of the pterygoid; pt-pal, palatine shelf of the pterygoid; ptf, pterygoid flange; ptqp, quadrate process of the pterygoid; ptt, pterygoid teeth; sof, suborbital fenestra. Scale bars = 1 mm

The pterygoid (Fig. 20) is a paired bone that contacts the palatine and ectopterygoid anteriorly, epipterygoid dorsally, sphenoid medially, and quadrate posteromedially. It does not contact the quadrate in *A. occidentalis*. The general shape is triradiate, though it varies substantially between genera.

The palatine process is broad and triangular in *Typhlacontias*, contacting the palatine on its dorsal side. The pterygoid flange curves anterolaterally to contact the ectopterygoid. It is broader and more robust with a dorsal curve in *T. brevipes* (Fig. 20A). The pterygoid flange and palatine process form part of the suborbital fenestra’s posterolateral border. The fossa columella, which receives the epipterygoid, is located 56% and 44% of the length from the anterior most point of the pterygoid in *T. brevipes* and *T. gracilis* (Fig. 20B) respectively. The quadrate process curves laterally in both species to contact the ventromedial side of the quadrate. It is broader in *T. brevipes* than *T. gracilis*. At the posterior end in *T. brevipes*, the quadrate process flares out vertically. The quadrate process of *T. gracilis* retains a similar height throughout and ends in a broad rounded tip.

As in *Typhlacontias*, the pterygoid of *S. alberti* has three distinct processes (Fig. 20C). However, it is much wider with a different shape. The palatine process is broad and rectangular, contacting the palatine dorsally. It has a medial slant, making the posterior ends closer together than the anterior ones. The pterygoid flange is considerably longer than in *Typhlacontias*. It is covered by the ectopterygoid for most of its ventral surface and part of its medial surface. Its anterior end narrows to a point, which fits into a divot in the ectopterygoid. The pterygoid flange and the palatine process form the posterior margin of the suborbital fenestra. The fossa columella is located roughly 64% of the length of the pterygoid, which is further posterior than the other species. The quadrate process contacts the ventromedial side of the quadrate. It is broader than in *T. gracilis*, but does not flare out as much as in *T. brevipes*. *Sepsina alberti* is the only examined species with pterygoid teeth. It has two small, triangular pterygoid teeth located near the junction of the palatine process and pterygoid flange.

The pterygoid of *A. occidentalis* is quite different from the scincines as it only has two distinct processes, giving it a crescent shape (Fig. 20D). Rather than a palatine process, *A. occidentalis* has a palatine shelf, an elliptical, depressed region upon which the palatine sits. The pterygoid flange is longer and curves anterolaterally, contacting the ectopterygoid medially and palatine ventrally. The fossa columella is located about 43% of the length of the pterygoid. The quadrate process curves posterolaterally. It has a broad triangular tip, and a flat dorsal surface which overhangs the curved ventral side.

##### Ectopterygoid

**Fig. 21:**
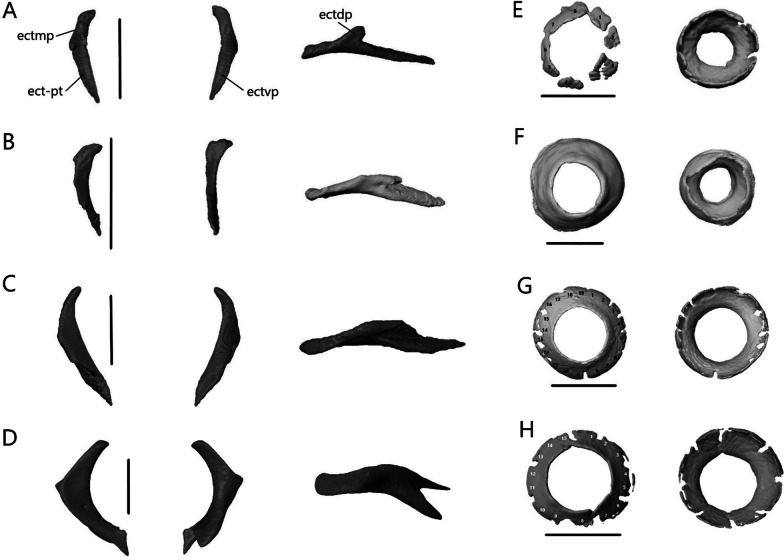
Isolated ectopterygoid and scleral ring. Left ectopterygoid of **A** *T. brevipes*; **B** *T. gracilis*; **C** *S. alberti*; **D** *A. occidentalis*; in dorsal, ventral, and lateral views from left to right. Left scleral ring of **E** *T. brevipes*; **F** *T. gracilis*; **G** *S. alberti*; **H** *A. occidentalis*; in lateral (left) and medial (right) views. ect-pt, pterygoid facet of the ectopterygoid; ectdp, dorsal process of the ectopterygoid; ectmp, maxillary process of the ectopterygoid; ectvp, ventral process of the ectopterygoid; Scale bars = 1 mm

The ectopterygoid (Fig. 21A–D) is a paired, crescent-shaped bone that contacts the maxilla anteriorly and pterygoid posteriorly. It contacts the jugal anterolaterally in *T. gracilis* and palatine posteriorly in *A. occidentalis*. It forms the lateral border of the suborbital fenestra.

The maxillary process curves medially and ends near the maxilla-palatine suture. In *T. gracilis*, the maxillary process expands anteriorly (Fig. 21B). The posterior region has a triangular concavity that clasps the pterygoid flange dorsally. It is more pronounced in *T. brevipes* (Fig. 21A). The posterior region splits into the dorsal and ventral processes, which border the pterygoid flange. The dorsal process is flattened and appears fused to the ventral process in *T. gracilis*. The ventral process has a slight medial curve to it.

The curvature of the ectopterygoid in *S. alberti* (Fig. 21C) is more pronounced than in *Typhlacontias*. The maxillary process curves medially, ending in a rounded point where the posterior process of the maxilla splits off. The lateral margin of the maxillary process is somewhat flattened. The dorsal process is fused to the ventral process, similar to the condition in *T. gracilis*. The difference is that the concavity in which the pterygoid flange fits is expanded. The ventral process curves medially and is broader than in *Typhlacontias*.

The maxillary process is relatively broader and shows greater curvature in *A. occidentalis* than in the others (Fig. 21D). Its lateral side is flattened and slightly concave, creating a facet for the maxilla. The posterior region forks into two distinct processes: the longer dorsal process, which contacts the pterygoid, and the shorter ventral process, which contacts the palatine. Both processes have been dorsoventrally compressed, particularly the dorsal process. The ectopterygoid is more curved with a larger dorsal process in *A. occidentalis*.

##### Scleral ring

The scleral ossicles (Fig. 21E–H) are small bones that form a ring within the eye. Segmentation of individual ossicles was challenging, as they were rarely distinct. This may be an artifact of the scan or may indicate a high level of fusion, as seen in other burrowers [53].

*Typhlacontias brevipes* has at least 8 ossicles, although some are likely the result of fusion (Fig. 21E). They are roughly the same size and shape. Individual ossicles in *T. gracilis* could not be distinguished (Fig. 21F). The aperture diameter is 64% of the external ring diameter in *T. brevipes* and 54% in *T. gracilis*.

*Sepsina alberti* has 19 ossicles (Fig. 21G). There seems to be a lot of fusion, particularly along the interior margin. The ossicles are narrow and rectangular. The aperture diameter is roughly 60% of the external ring diameter.

*Acontias occidentalis* has at least 15 ossicles (Fig. 21H), although there may be more that could not be distinguished. Many are fused together. The aperture diameter is roughly 59% of the external ring diameter.

#### Description of isolated splanchonocranial bones

##### Quadrate

**Fig. 22:**
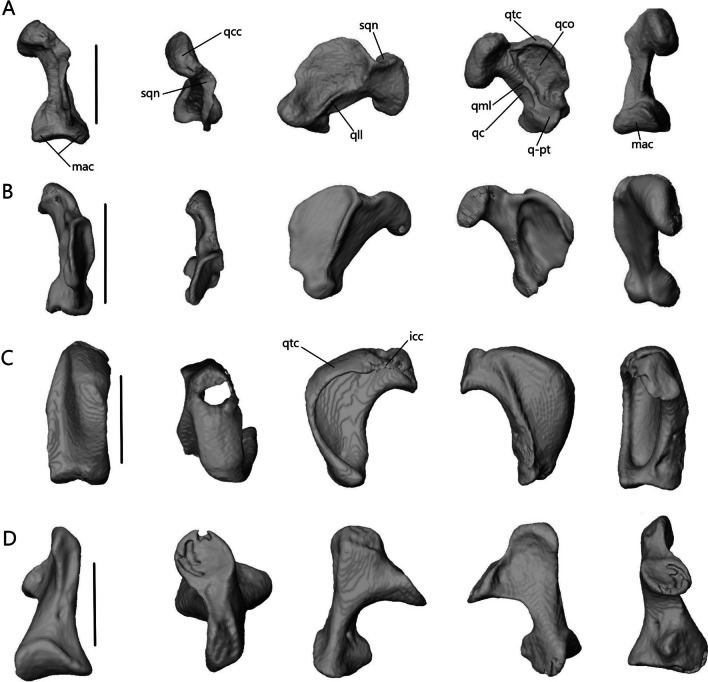
Isolated left quadrate of **A** *T. brevipes*; **B** *T. gracilis*; **C** *S. alberti*; **D** *A. occidentalis*; in anterior, dorsal, lateral, medial, and posteroventral views from left to right. icc, intercalary cartilage; mac, mandibular condyle of the quadrate; q-pt, pterygoid facet of the quadrate; qc, quadrate medial column; qcc, cephalic condyle of the quadrate; qco, quadrate conch; qll, lateral lamina of the quadrate; qml, medial lamina of the quadrate; qtc, tympanic crest; sqn, squamosal notch. Scale bars = 1 mm

The quadrate (Fig. 22) plays an important role in supporting the peripheral auditory system, in cranial kinesis, and in jaw biomechanics, both as a muscle attachment site and as the sole articulation between the lower jaw and cranium [13, 54]. It is a paired bone that contacts the squamosal and supratemporal dorsally, the otoccipital dorsomedially, the pterygoid medially, and the articular ventrally. It does not contact the squamosal or pterygoid in *A. occidentalis*.

In lateral view, the quadrate is D-shaped in *Typhlacontias* (Fig. 22A, B). The conch is flat, with only a slight medial curvature to it. The cephalic condyle is positioned posteromedially and contacts the paroccipital process of the otoccipital and the supratemporal. It is large and bulbous, especially in *T. brevipes* where it forms a ventral knob. The squamosal notch is anterior to the cephalic condyle and serves as the articulation point for the squamosal. The mandibular condyle comprises distinct medial and lateral condyles, which articulate with the lower jaw. A medial column extends between the cephalic condyle and mandibular condyle. The medial lamina runs along this column. It is more distinct in *T. brevipes*. The lateral lamina runs along most of the quadrate in *T. gracilis*, but is only clearly visible on the ventral half in *T. brevipes*. A pterygoid facet is present slightly dorsal of the medial mandibular condyle. The reduced tympanic crest projects medially along the conch. The quadrate is more recumbent in *T. brevipes* than in *T. gracilis*. The height (measured from the cephalic condyle to the mandibular condyle) is roughly two times the width (measured at the widest point of the conch) in both species.

The quadrate of *S. alberti* varies from the quadrate of *Typhlacontias* in many ways, although it has a similar D-shaped silhouette (Fig. 22C). The conch is more curved, with the tympanic crest curving laterally instead of medially. The cephalic condyle is oriented medially and contacts the supratemporal and paroccipital process of the otoccipital. Its medial side is flattened. Directly anterior, the squamosal notch is a distinct foramen. The squamosal articulates with its posterior margin. The lateral side of the squamosal notch is closed by the intercalary cartilage. The medial and lateral mandibular condyles are narrower than those in *Typhlacontias* and *A. occidentalis*. The medial column is more curved in *S. alberti* than *Typhlacontias*. The lateral lamina is not present, but the medial lamina is very distinct. The pterygoid facet is present posterior to the medial lamina and slightly dorsal of the mandibular condyle. The quadrate is less recumbent than in *Typhlacontias*. The height is roughly twice the width.

The quadrate of *A. occidentalis* has a very different shape than that of the scincines (Fig. 22D). The reduced conch is roughly triangular. Its dorsal edge forms the highly reduced and medially curving tympanic crest, similar to *Typhlacontias* but smaller. The cephalic condyle is oriented posterodorsally and cups the paroccipital process and supratemporal. It is slightly concave and circular. The squamosal notch is present as an indentation between the cephalic condyle and tympanic crest, but it does not contact the squamosal. The mandibular condyle, made up of medial and lateral condyles, forms a broad base, larger than in the other skinks. The medial column runs along the posterior side and is more curved in the scincines. The quadrate is less recumbent than in *Typhlacontias*, but more than in *S. alberti*. The height is roughly one and a half times the width.

##### Epipterygoid

**Fig. 23:**
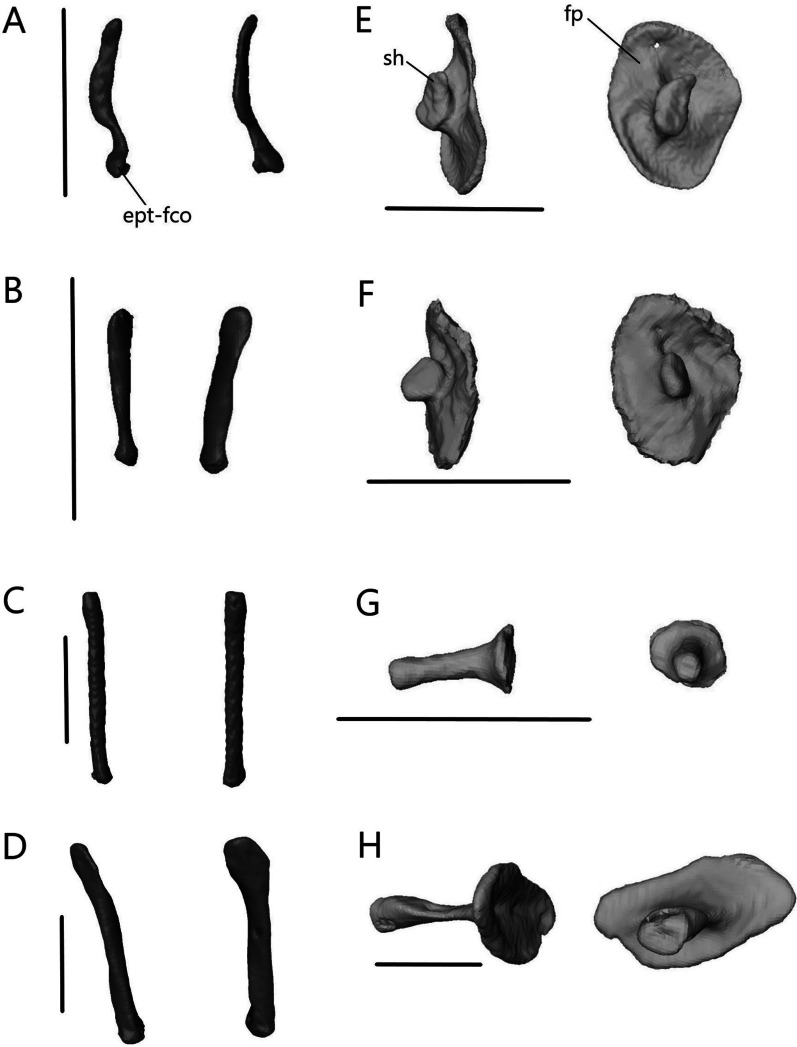
Isolated epipterygoid and stapes. Right epipterygoid of **A** *T. brevipes*; **B** *T. gracilis*; **C** *S. alberti*; **D** *A. occidentalis*; in anterior (left) and lateral (right) views. Left stapes of **E** *T. brevipes*; **F** *T. gracilis*; **G** *S. alberti*; **H** *A. occidentalis*; in posterior (left) and lateral (right) views. ept-fco, pterygoid facet of the epipterygoid; fp, oval footplate of the stapes; sh, shaft of the stapes. Scale bars = 1 mm

The epipterygoid (Fig. 23A–D) is a paired, rod-like bone that extends from the fossa columella of the pterygoid to a region near the descending process of the parietal and the crista alaris of the prootic. In *T. gracilis*, it contacts the parietal medially.

The epipterygoid has a ventral expansion where it contacts the pterygoid. In *T. gracilis*, the epipterygoid is thick and columnar with a slight constriction near the center and a rounded top (Fig. 23B). The epipterygoid of *T. brevipes* is smaller, with an irregular, curved shape (Fig. 23A). As this is an endochondral element, it is possible that the shape is an artifact due to the inability of the scan to capture cartilaginous portions of the epipterygoid.

The epipterygoid of *S. alberti* is narrow and columnar, nearly the same width throughout (Fig. 23C). The base is rounded and the top is flattened.

The epipterygoid of *A. occidentalis* has a slight lateral tilt, most visible in anterior view (Fig. 23D). It is thick and columnar, with a large paddle-like expansion at the dorsal end. The base is rounded as in the other species.

##### Stapes

The stapes (or columella; Fig. 23E–H) forms part of the middle ear and is a paired bone. It consists of a footplate, which sits in the fenestra ovalis, and a shaft which extends perpendicularly from the footplate.

The footplate is large and roughly circular with a concave surface in *Typhlacontias*. The short and broad shaft ends with a flat top that nearly contacts the quadrate in *T. brevipes* (Fig. 23E). In *T. gracilis*, the shaft has a more rounded top (Fig. 23F).

Of the examined species, *S. alberti* has the smallest stapes (Fig. 23G). The footplate is circular and does not fill the entire fenestra ovalis, possibly because part of it is cartilaginous. The shaft is columnar with a flat end.

The stapes of *A. occidentalis* has an elliptical footplate (Fig. 23H). Although large, it is still smaller than in *Typhlacontias*. The shaft is longer and thinner than in the other species. It is dorsoventrally compressed but broadens out at the flattened end.

##### Hyoid apparatus

**Fig. 24:**
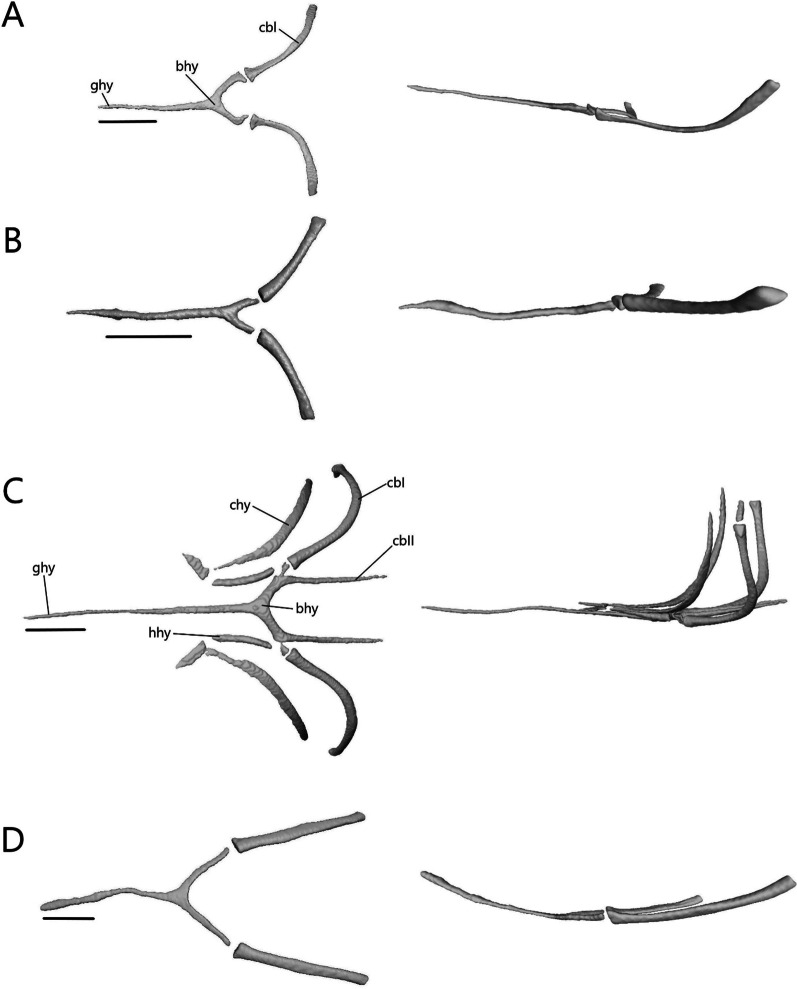
Isolated hyoid apparatus of **A** *T. brevipes*; **B** *T. gracilis*; **C** *S. alberti*; **D** *A. occidentalis*; in ventral and lateral view. bhy, basihyal; cbI, first ceratobranchial; cbII, second ceratobranchial; chy, ceratohyal; ghy, glossohyal; hhy, hypohyal. Scale bars = 1 mm

The hyoid apparatus (Fig. 24) consists of multiple elongate ossified and cartilaginous elements located in the posteroventral region of the head and extending into the neck. They do not contact any bones. Only *S. alberti* shows the full complement of elements, although in the other species, the missing elements may be cartilaginous and not visible in the CT scans.

Both *Typhlacontias* show a single glossohyal and basihyal with one pair of ceratobranchials, forming a Y-shaped hyoid apparatus. The glossohyal and basihyal are stick-like elements that together extend roughly to the level of the anterior margin of the sphenoid. In *T. brevipes*, the glossohyal and basihyal look nearly continuous with only a slight constriction where they meet (Fig. 24A). In *T. gracilis*, the basihyal is thicker and there is a bulge where the glossohyal begins (Fig. 24B). The glossohyal narrows to a point and the basihyal has a posterior bifurcation. The tips of the bifurcation arch inwards in *T. brevipes*. The ceratobranchials originate here and arch laterally. Near their center, the ceratobranchials also curve dorsally although this is less extreme in *T. gracilis*. The ceratobranchials of *T. gracilis* are more robust than in *T. brevipes*.

Of the examined skinks, *S. alberti* is the only one to have all the elements of the hyoid apparatus, including a single basihyal and glossohyal, and paired first ceratobranchials, second ceratobranchials, ceratohyals, and hypohyals (Fig. 24C). The narrow glossohyal stems from the basihyal and extends to the posterior margin of the palatine. The basihyal is Y-shaped, with a posterior bifurcation. Three elements originate from each tip: the hypohyal anteriorly, the first ceratobranchial posterolaterally, and the second ceratobranchial posteromedially. The hypohyal has rounded ends and a slight arch. The posterior half of the first ceratobranchial curves dorsolaterally, turning vertical and reaching roughly the same level as the lower margin of the orbit. The ends are broad and flat. The second ceratobranchial is elongate and extends posteriorly with a slight dorsal tilt. It narrows posteriorly. The ceratohyal is anterior to the first ceratobranchial and lateral to the hypohyal. It has a narrow crescent shape and runs parallel to the first ceratobranchial. A flattened, trapezoidal chip of bone is present near the anterior end of the ceratohyal. In addition to being more complex than in the other species, the hyoid apparatus of *S. alberti* has more elongate and gracile elements.

The hyoid apparatus of *A. occidentalis* (Fig. 24D) is very similar to that of *Typhlacontias*. The glossohyal extends to the level of the posterior region of the palatine. The basihyal’s bifurcating ends are larger and narrower than in the other species. The first ceratobranchial is the most robust of the elements and retains a similar thickness throughout. It has a slight dorsal curve, but does not curve as much as in the other species.

#### Neurocranium

**Fig. 25:**
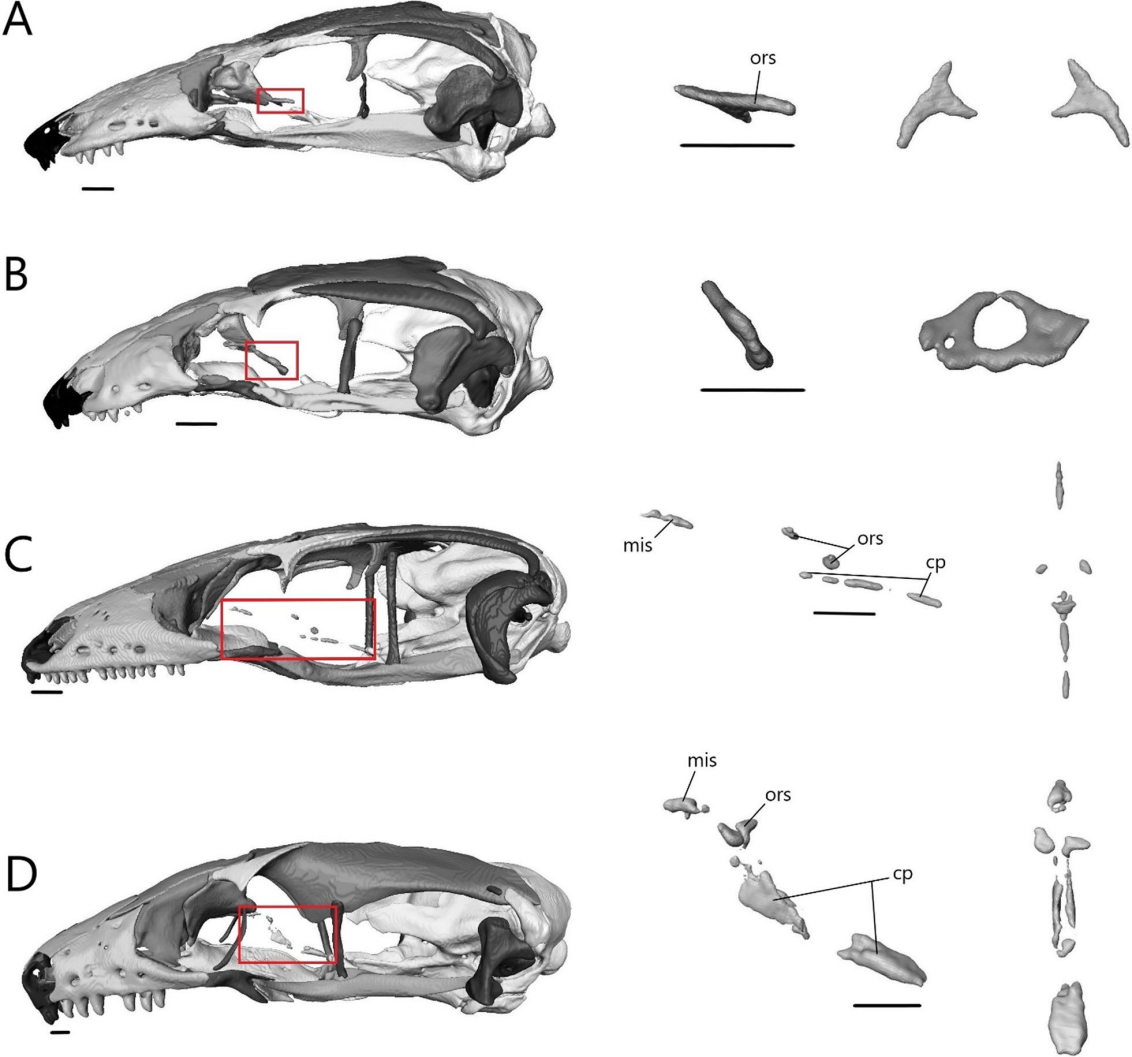
To the left, the orbitotemporal region of the neurocranium within the dermatocranium of *T. brevipes*, *T. gracilis*, *S. alberti*, and *A. occidentalis* from top to bottom in lateral view. The region is boxed in red. To the right, isolated orbitotemporal elements in lateral (left) and posterodorsal (right) views. **A** *T. brevipes*; **B** *T. gracilis*; **C** *S. alberti*; **D** *A. occidentalis*. cp, cultriform process; mis, medial interorbital septum; ors, orbitosphenoids. Scale bars = 0.5 mm

**Fig. 26:**
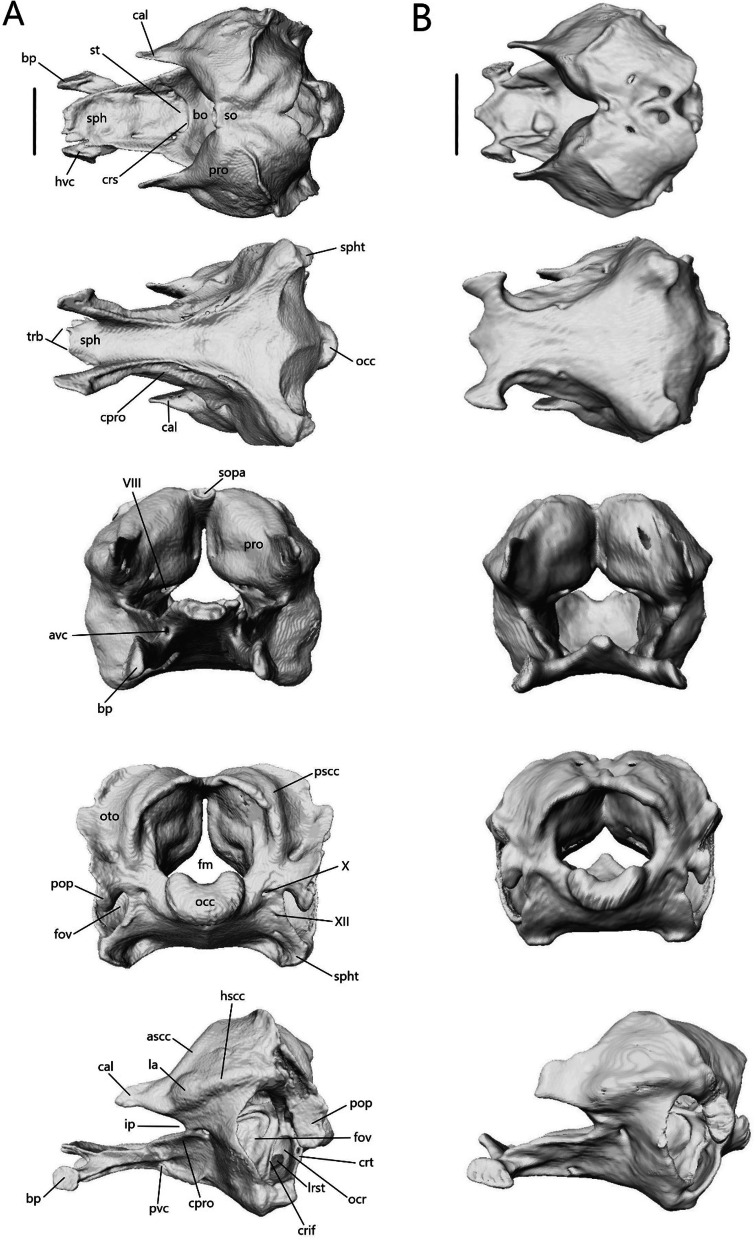
Isolated otoccipital neurocranium of **A** *T. brevipes* and **B** *T. gracilis*; in dorsal, ventral, anterior, posterior, and lateral views from top to bottom. ascc, anterior semicircular canal; avc, anterior opening of the vidian canal; bo, basioccipital; bp, basipterygoid process; cal, crista alaris; cpro, crista prootica; crif, crista interfenestralis; crs, crista sellae; crt, crista tuberalis; fm, foramen magnum; fov, fenestra ovalis; hscc, horizontal semicircular canal; hvc, lateral head vein; ip, incisura prootica; la, lateral ampulla; lrst, lateral opening of the recessus scalae tympani; occ, occipital condyle; ocr, occipital recess; oto, otooccipital; pop, paroccipital process; pro, prootic; pscc, posterior semicircular canal; pvc, posterior opening of the vidian canal; so, supraoccipital; sopa, facet for the processus ascendens; sph, sphenoid; spht, sphenooccipital tubercle; st, sella turcica; trb, trabecula; VII, foramen of CN VII; X, foramen of CN X; XII, foramen of CN XII. Scale bars = 1 mm

**Fig. 27:**
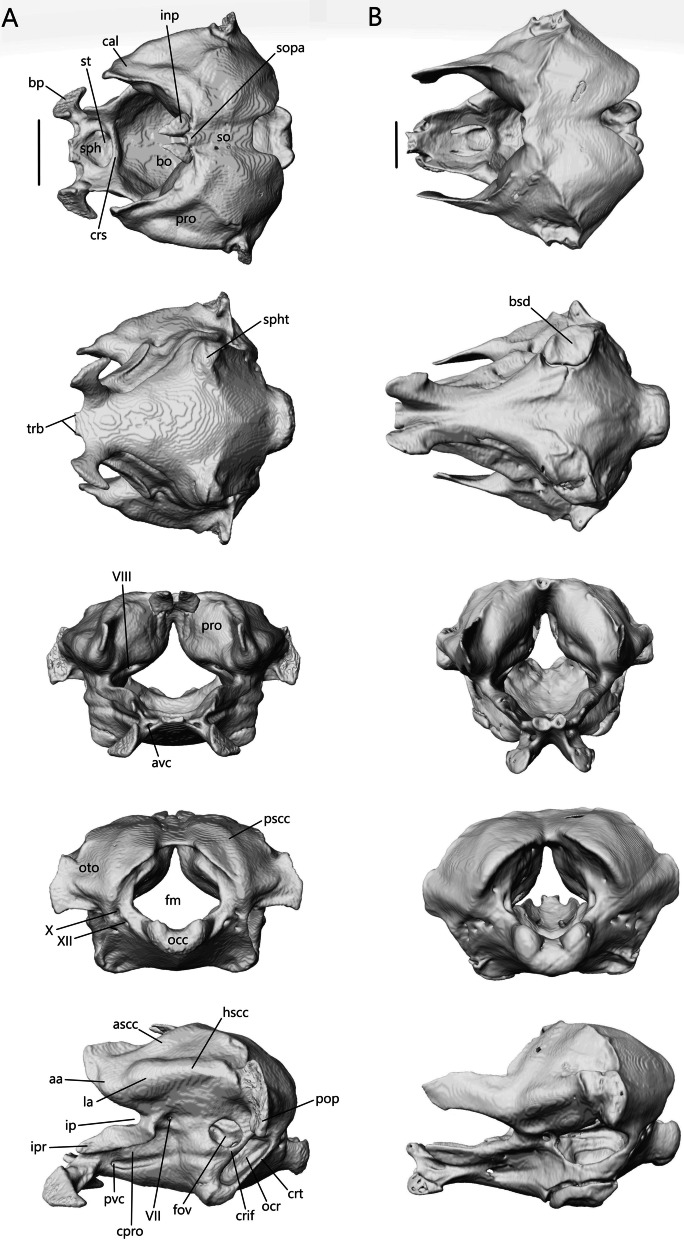
Isolated otoccipital neurocranium of **A** *S. alberti*; **B** *A. occidentalis*; in dorsal, ventral, anterior, posterior, and lateral views from top to bottom. aa, anterior ampulla; ascc, anterior semicircular canal; avc, anterior opening of the vidian canal; bo, basioccipital; bp, basipterygoid process; bsd, basicranial sesamoid; cal, crista alaris; cpro, crista prootica; crif, crista interfenestralis; crs, crista sellae; crt, crista tuberalis; fm, foramen magnum; fov, fenestra ovalis; hscc, horizontal semicircular canal; hvc, lateral head vein; inp, interparietal; ip, incisura prootica; ipr, inferior process of the prootic; la, lateral ampulla; lrst, lateral opening of the recessus scalae tympani; occ, occipital condyle; ocr, occipital recess; oto, otooccipital; pop, paroccipital process; pro, prootic; pscc, posterior semicircular canal; pvc, posterior opening of the vidian canal; so, supraoccipital; sopa, facet for the processus ascendens; sph, sphenoid; spht, sphenooccipital tubercle; st, sella turcica; trb, trabecula; VII, foramen of CN VII; X, foramen of CN X; XII, foramen of CN XII. Scale bars = 1 mm

The neurocranium is divided into the orbitotemporal region (Fig. 25), represented by ossified and cartilaginous elements located centrally in the skull, and the otoccipital region, represented by a single fused structure called the braincase (Figs. 26, 27). The orbitotemporal region consists of the medial interorbital septum, planum supraseptale, fused or paired orbitosphenoids, and cultriform process. Many elements are partly or entirely cartilaginous in the examined species, with the planum supraseptale entirely cartilaginous in all four.

The paired orbitosphenoids of *Typhlacontias* lie directly posterior to the cristae cranii of the frontal. They are well-ossified. The orbitosphenoids of *T. brevipes* are unfused (Fig. 25A). They are y-shaped with the longest process extending to the sphenoid. They lie in the horizontal plane. The orbitosphenoids of *T. gracilis* have fused into a ring structure with two lateral wings (Fig. 25B). They lie at a roughly 45° angle from the horizontal.

The elements of the orbitotemporal region are only partially ossified in *S. alberti* (Fig. 25C). The medial interorbital septum is a small, rod-like bone located between the orbits. The orbitosphenoids show little ossification with two small, lateral pieces located anterior to a larger medial piece. They are oriented at an angle. The cultriform process extends from the sphenoid to a region ventral to the orbitosphenoids. It is long and narrow, with four ossified pieces.

The elements of the orbitotemporal region show partial ossification in *A. occidentalis* (Fig. 25D), although they are more ossified than in *S. alberti*. The medial interorbital septum is located directly posterior of the cristae cranii. The orbitosphenoids comprise two ossified pieces set at an angle. The cultriform process extends from the sphenoid to the orbitosphenoids in three main pieces. Anteriorly, there are two elongate, flattened pieces which are lateral to one another. Posterior to these pieces, there is a single, broader piece.

The braincase is formed by the fusion of three medial unpaired elements (sphenoid, basioccipital, and supraoccipital) and two lateral paired elements (prootic and otooccipital). It is covered dorsally by the parietal and contacts the quadrate, pterygoid, supratemporal, stapes, atlas, and axis (except in *A. occidentalis*). The sphenoid and basioccipital form a concave ventral floor. The prootics, otooccipitals, and supraoccipital form the roof and walls of the brain cavity. The foramen magnum is a large, circular opening in the posterior side. Its boundaries are delimited by the supraoccipital dorsally, otooccipitals laterally, and basioccipital ventromedially. The occipital condyle, located ventral to the foramen magnum, is formed by the otooccipitals laterally and basioccipital medially, and articulates with the atlas and (in the scincines) the odontoid process of the axis. In *A. occidentalis*, the odontoid process has been greatly reduced, so this contact is lost. The inner ear cavities are formed by the prootics, supraoccipital, and otooccipitals. The external opening to the inner ear is the fenestra ovalis, which is located between the prootic and otooccipital.

The width of the braincase is 63% and 66% of its length in *T. brevipes* and *T. gracilis*, respectively, making their braincases relatively longer than in the other species. In *T. brevipes*, the walls of the braincase are separated by a narrow medial gap that continues to the roof of the supraoccipital (Fig. 26A). In *T. gracilis*, they meet and fuse further down (Fig. 26B). The foramen magnum is larger and circular in *T. brevipes*. In *T. gracilis*, it is somewhat flattened dorsally. The occipital condyle is larger in *T. brevipes*, but has a smaller anterior notch.

The braincase of *S. alberti* is wider than it is long, with the width 112% of the length (Fig. 27A). The brain cavity has a medial gap similar to the one in *T. brevipes*, although wider and not as long. The foramen magnum is more ovoid than in *Typhlacontias*. The occipital condyle is the smallest of the examined species, but similar in shape to *T. gracilis*.

The width of the braincase is 85% of the length in *A. occidentalis* (Fig. 27B). It is compressed. In anterior view, the brain cavity has a V-shaped medial gap and appears narrower than in the other species. The foramen magnum is ovoid with a greater height than width. The occipital condyle is very large and sutures can be seen on its dorsal surface.

##### Sphenoid

The sphenoid (Figs. 26, 27) forms the anterior region of the braincase. It is formed by the fusion of the basisphenoid and parasphenoid, and contacts the basioccipital posteriorly, prootic dorsally, and pterygoid anterolaterally. The sphenoid has a wide and deep depression called the sella turcica, which houses the pituitary gland.

There is complete fusion between the basioccipital and sphenoid in *Typhlacontias*. Anteromedially, the sphenoid is broad with small paired trabeculae in *T. brevipes* (Fig. 26A). In *T. gracilis*, the sphenoid narrows to a flattened tip and there are no trabeculae (Fig. 26B). The anterior openings for the vidian canal are located ventral to the trabeculae. They are not visible in *T. gracilis*, likely due to their small size. The basipterygoid processes point anterolaterally in both species, but extend further anteriorly in *T. brevipes*. The distal ends are expanded and contact the pterygoid. They are circular in *T. brevipes* and more elliptical in *T. gracilis*. The basipterygoid processes are squat, making up 13% and 18% of the total braincase length in *T. brevipes* and *T. gracilis* respectively. The sella turcica is located at the posterior region. Its posterior border, the crista sellae, forms a slight ridge. Anterior to this ridge, the sphenoid of *T. brevipes* has four lateral foramina and *T. gracilis* two foramina. The posterior set of foramina are the posterior openings of the vidian canal whereas the anterior ones in *T. brevipes* are the internal continuation of the anterior openings of the vidian canal. The posterior openings open on the ventrolateral wall of the sphenoid. Overall, the sphenoid of *T. brevipes* is relatively longer than that of *T. gracilis*.

The sphenoid of *S. alberti* (Fig. 27A) is relatively shorter than in *Typhlacontias*. It is completely fused with the basioccipital. The small anterior edge has paired, triangular trabeculae. The anterior openings for the vidian canal are located lateral to the trabeculae. The basipterygoid processes point anterolaterally. They are similar in shape to those of *T. gracilis*, but larger, with expanded posterior sides. They contact the basipterygoid facets of the pterygoid along their flattened medial sides. The basipterygoid processes make up 23% of the total braincase length. The sella turcica is very prominent. It is located directly posterior to the anterior margin. The crista sellae is more distinct than in *Typhlacontias* and has a slight concave curvature. On the lateral sides of the sella turcica, there are partially open canals, which run between the anterior and posterior openings of the vidian canal.

The sphenoid of *A. occidentalis* (Fig. 27B) is relatively longer than in *T. gracilis* or *S. alberti*, but not as long as *T. brevipes*. A suture is evident between the sphenoid and basioccipital on the ventral side. The sphenoid narrows anteromedially and forms two triangular trabeculae. The anteromedial sides are slightly concave and circular, articulating with the cultriform process. The anterior openings for the vidian canal are located posteroventral to the trabeculae. The short, broad basipterygoid processes point anterolaterally, although they barely extend further than the trabeculae anteriorly. The distal ends are broad and rounded. They are a similar width for their entire length. They make up about 19% of the total braincase length. The sella turcica is prominent, as in *S. alberti*, and is located on the posterior half of the sphenoid. The depression is subrectangular and bordered laterally by distinct ridges. The crista sellae forms a concave wall. Lateral to the sella turcica, there are half-open canals connecting the openings of the vidian canal.

##### Basioccipital

The basioccipital (Figs. 26, 27) forms the posterior portion of the brain cavity’s floor and contacts the sphenoid anteriorly, otooccipital laterally, and atlas and axis posteriorly. Its dorsal surface is concave and ventrolaterally it curves out to form the sphenooccipital tubercles. The otooccipitals also participate in forming these tubercles.

The basioccipital is wider than it is long in both *Typhlacontias*. Ridges form a rounded W-shape on the ventral side in *T. brevipes* (Fig. 26A). These are not seen in *T. gracilis* (Fig. 26B). The sphenooccipital tubercles protrude out more in *T. brevipes*. In *T. gracilis*, they are smaller and have flatter ventral surfaces.

The general shape of the basioccipital of *S. alberti* (Fig. 27A) is more similar to the basioccipital of *T. gracilis* than of *T. brevipes*. The occipital condyle and sphenooccipital tubercles are much smaller than in *Typhlacontias*.

The basioccipital of *A. occidentalis* (Fig. 27B) is quite similar to that of the examined scincines, especially *T. brevipes*. The occipital condyle is large and protrudes more than in the other species. The sphenooccipital tubercles are also larger. They are capped by basicranial sesamoids (or element X). These sesamoids are elliptical in shape.

##### Supraoccipital

The supraoccipital (Figs. 26, 27) forms the arched roof of the braincase and the dorsal border of the foramen magnum. It contacts the prootics anteriorly and otooccipitals ventrolaterally. It houses part of the anterior and posterior semicircular canals as well as the common crus. Impressions of these can be seen on the dorsal surface in *Typhlacontias* and *Sepsina*. The dorsal surface is concave and the overall shape is roughly trapezoidal to hexagonal.

The supraoccipital has a medial notch in its posterior margin. This notch is smaller and V-shaped in *T. brevipes* (Fig. 26A) and U-shaped in *T. gracilis* (Fig. 26B). *Typhlacontias gracilis* also has two large foramina, one on either side of the notch. Anteriorly, there is an ovoid medial facet where the cartilaginous processus ascendens contacts the supraoccipital. Additionally, there are distinct ridges running laterally on the dorsal surface.

The posterior margin has a rectangular medial notch in *S. alberti* (Fig. 27A), larger than in the other species. There are three small foramina located on the dorsal surface. The facet for the processus ascendens is located anteriorly and flanked by the interparietals.

The dorsal surface is not as concave as in the other skinks, creating a flatter roof in *A. occidentalis* (Fig. 27B). The concavity becomes more pronounced at the posterior end. The medial notch is deep and U-shaped. The facet for the processus ascendens is a circular medial depression on the anterior side.

##### Prootic

The paired prootic (Figs. 26, 27) forms the anterodorsal region of the braincase. It contacts the sphenoid anteroventrally, supraoccipital posterodorsally, and otooccipital posteroventrally. The prootic houses the anterior parts of the inner ear including the anterior semicircular canal, horizontal semicircular canal, anterior ampulla, lateral ampulla, and the anterior region of the vestibule. It also forms the anterior margin of the fenestra ovalis.

**Fig. 28:**
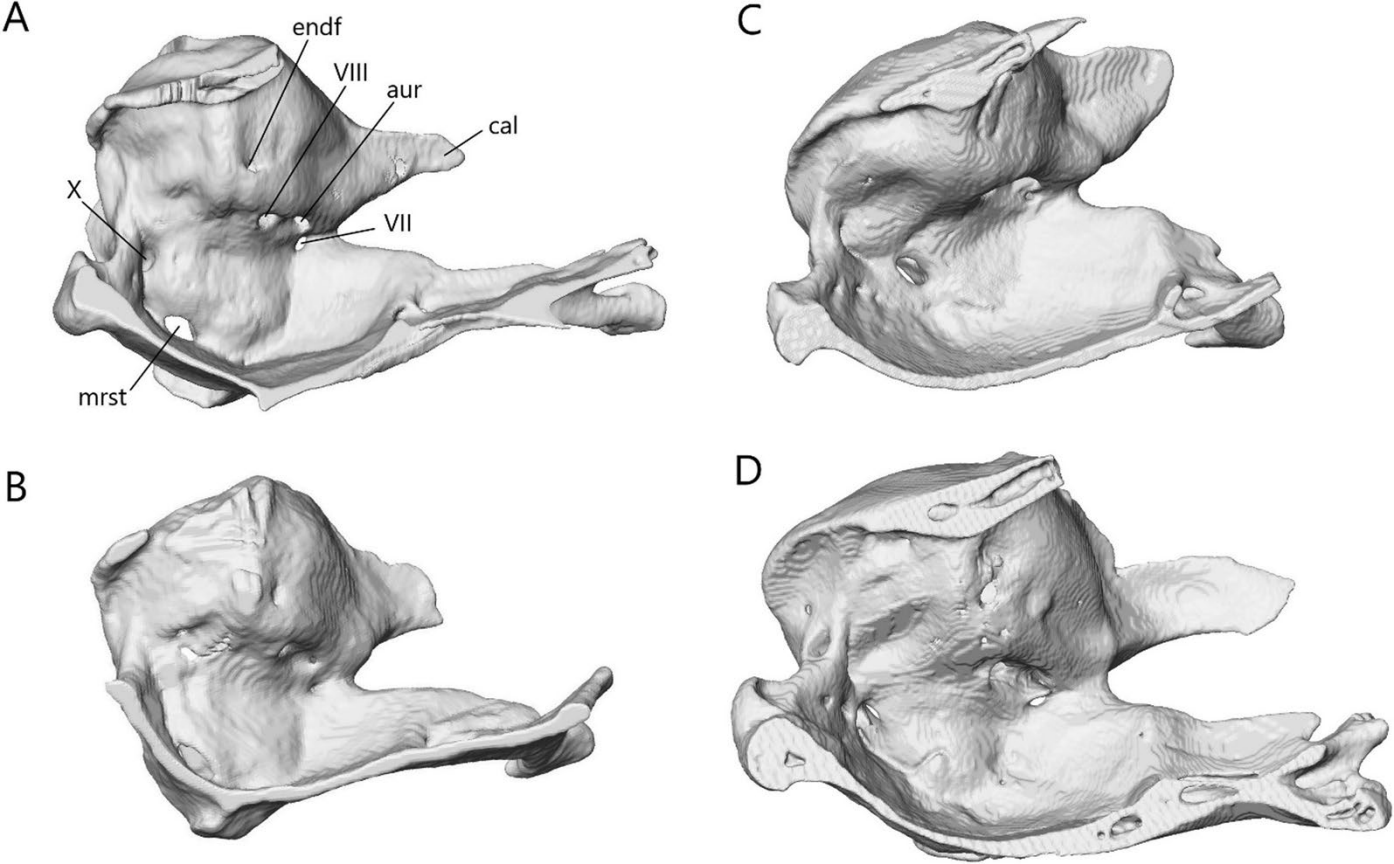
Isolated otooccipital region of the neurocranium **A** *T. brevipes*; **B** *T. gracilis*; **C** *S. alberti*; **D** *A. occidentalis*; in cut-away medial view. aur, auditory recess of the prootica; cal, crista alaris; endf, endolymphatic foramen; mrst, medial opening of recessus scalae tympani; VII, foramen of CN VII; VIII, foramen of CN VIII; X, foramen of CN X

The crista alaris (or alar process) extends anteriorly towards the epipterygoid, but does not contact either it or the descending process of the parietal (Fig. 5A). The crista alaris is broader vertically in *T. gracilis* (Fig. 26B), closing more of the lateral wall of the braincase, than in *T. brevipes* (Fig. 26A). Ventral to this, there is a deep, V-shaped notch, the incisura prootica. The crista prootica extends anteriorly to contact the sphenoid and posteriorly to the otooccipital. On its medial side (Fig. 28A, B), the prootic has openings for the facial (CN VII) and auditory (CN VIII) nerves within the auditory recess. The endolymphatic foramen is dorsal to the foramen for the auditory nerve. The anterior semicircular canal, horizontal semicircular canal, and lateral ampulla are visible along the prootic’s surface.

The prootic of *S. alberti* (Fig. 27A) is similar in overall shape to that of *Typhlacontias*. The crista alaris extends anteriorly. It curves dorsally, giving it an upturned appearance. The crista alaris curves posteriorly to form the dorsal margin of the incisura prootica, a deep, U-shaped notch. Its ventral margin is formed by the inferior process of the prootic. This process is either absent or greatly reduced in *Typhlacontias*, but it is large and extends to the base of the basipterygoid processes in *S. alberti*. The inferior process is uniform in width with a slight bulge near its center. Ventral to this, the crista prootica contacts the sphenoid anteriorly and the otooccipital posteriorly. It also forms the anterior margin of the fenestra ovalis and contains the small lateral foramen for the facial nerve. Medially, the prootic has openings for the facial and auditory nerves in a similar arrangement to *Typhlacontias* (Fig. 28C). The endolymphatic foramen is either not present or not visible in the scan. The same elements of the inner ear are visible on the prootic’s surface as in *Typhlacontias*, with the addition of the anterior ampulla.

The crista alaris of *A. occidentalis* is narrower than in *T. gracilis* and *S. alberti*, but more elongate with a blunt end (Fig. 27B). The crista alaris forms the dorsal margin of the incisura prootica, which is V-shaped and relatively larger than in the other species. The inferior process forms the ventral margin of the incisura prootica. Its ventral margin is curved, creating a slight uplift at the tip. The crista prootica forms the anterior margin of the large fenestra ovalis. Only a narrow piece of bone separates the facial foramen and fenestra ovalis. The facial foramen is also larger. Medially, the prootic of *A. occidentalis* has the same foramina as seen in *Typhlacontias* (Fig. 28D). However, the foramen for the facial nerve is located further ventrally from the auditory nerve and the endolymphatic foramen is larger. Of the inner ear elements, the lateral ampulla and anterior semicircular canal are the most easily visible on the surface. The bulge of the lateral ampulla is directly posterior to the crista alaris and the anterior semicircular canal is visible on its ventral curve. They are less distinct than in the other species.

##### Otooccipital

The otooccipital (Figs. 26, 27) is a paired bone that is the result of fusion between the exoccipital and opisthotic. It forms the lateral border of the foramen magnum, the posterior wall of the otic capsule, the lateral portion of the occipital condyle, and the posterior border of the fenestra ovalis. It contacts the prootic anteriorly, basioccipital ventrally, supraoccipital dorsally, and quadrate and supratemporal laterally. The otooccipital houses the horizontal semicircular canal, the posterior semicircular canal, the posterior ampulla, and the posterior region of the vestibule.

The occipital recess (or recessus scalae tympani) is posterior to the fenestra ovalis, which is about twice the height of the occipital recess in *Typhlacontias* (Fig. 26). It is elliptical in shape. The large lateral opening of the recessus scalae tympani (or foramen rotundum) is located within the occipital recess. The medial opening of the recessus scalae tympani (or perilymphatic foramen) is located near the floor of the braincase (Fig. 28A, B). The divide between the fenestra ovalis and occipital recess is formed by a crest called the crista interfenestralis (Fig. 26). On the other side of the occipital recess, there is another crest, the crista tuberalis, which runs ventrally to the sphenooccipital tubercle. The paroccipital process is posterior to the fenestra ovalis and hosts a broad, elliptical facet which contacts the quadrate. In posterior view, there are two foramina ventrolateral to the foramen magnum (only one easily visible in *T. gracilis*). The larger dorsal foramen is the opening for the vagus nerve (CN X) and the ventral foramen is the opening for the hypoglossal nerve (CN XII). On the right side of the posterior view of *T. brevipes*, there is a third small foramen which may be a second opening for the hypoglossal nerve. The internal opening of the vagus nerve is on the medial wall of the otooccipital, just anterior to the foramen magnum (Fig. 28A). The horizontal semicircular canal is slightly visible, running laterally from the prootic. The posterior semicircular canal is more distinct as it runs along the dorsal surface of the braincase and then curves ventrally along its posterior side.

The otoccipital is very similar between *S. alberti* and *Typhlacontias* although the back curves out more in *S. alberti* (Fig. 27A). The occipital recess is posteroventral to the fenestra ovalis. Unlike in *Typhlacontias*, the occipital recess is larger than the fenestra ovalis in *S. alberti*. The occipital recess is elliptical in shape. The lateral opening of the recessus scalae tympani is located in the anterior region of the occipital recess. The medial opening is located more dorsally than in *Typhlacontias* (Fig. 28C). The crista interfenestralis and crista tuberalis run more diagonally in *S. alberti* than in *Typhlacontias* (Fig. 27A). The paroccipital process is relatively longer and contacts both the quadrate and supratemporal. There are three small foramina arranged in a triangle ventrolateral to the foramen magnum. The dorsal foramen is the opening for the vagus nerve and the two ventral foramina are openings for the hypoglossal nerve. The internal opening for the vagus nerve is located on the posteromedial wall, lateral to the foramen magnum (Fig. 28C). The horizontal semicircular canal is visible along the posterolateral wall posterior to the paroccipital process and the posterior semicircular canal is visible along the dorsal and posterior surface.

The otoccipital of *A. occidentalis* is larger with a posterior wall that curves out more than in the other species (Fig. 27B). The occipital recess is absent or fused with the fenestra ovalis. The large fenestra ovalis takes up much of the ventral region of the lateral wall, is oriented horizontally, and is ovoid in shape. It contains the lateral opening of the recessus scalae tympani on its posteromedial wall. The medial opening is set at the junction of the posterior and medial walls near the floor of the braincase (Fig. 28D). There is no crista interfenestralis, but the crista tuberalis runs along the posterior margin of the fenestra ovalis to the sphenoocipital tubercle (Fig. 27B). The crista tuberalis is broader than in the other species. The paroccipital process is oblong and relatively shorter than in *S. alberti* or *T. brevipes*. It is overlapped entirely by the supratemporal and only contacts the quadrate ventrally. *Acontias occidentalis* has five small foramina ventrolateral to the foramen magnum. The medial three are slightly larger. The dorsal one is the opening for the vagus nerve and the two ventral openings are for the hypoglossal nerve. The internal opening for the vagus nerve is posterodorsal to the medial opening of the recessus scalae tympani (Fig. 28D). Unlike in the other species, the horizontal semicircular canal is more distinct and the posterior semicircular canal is barely visible.

##### Osseous labyrinth

**Fig. 29:**
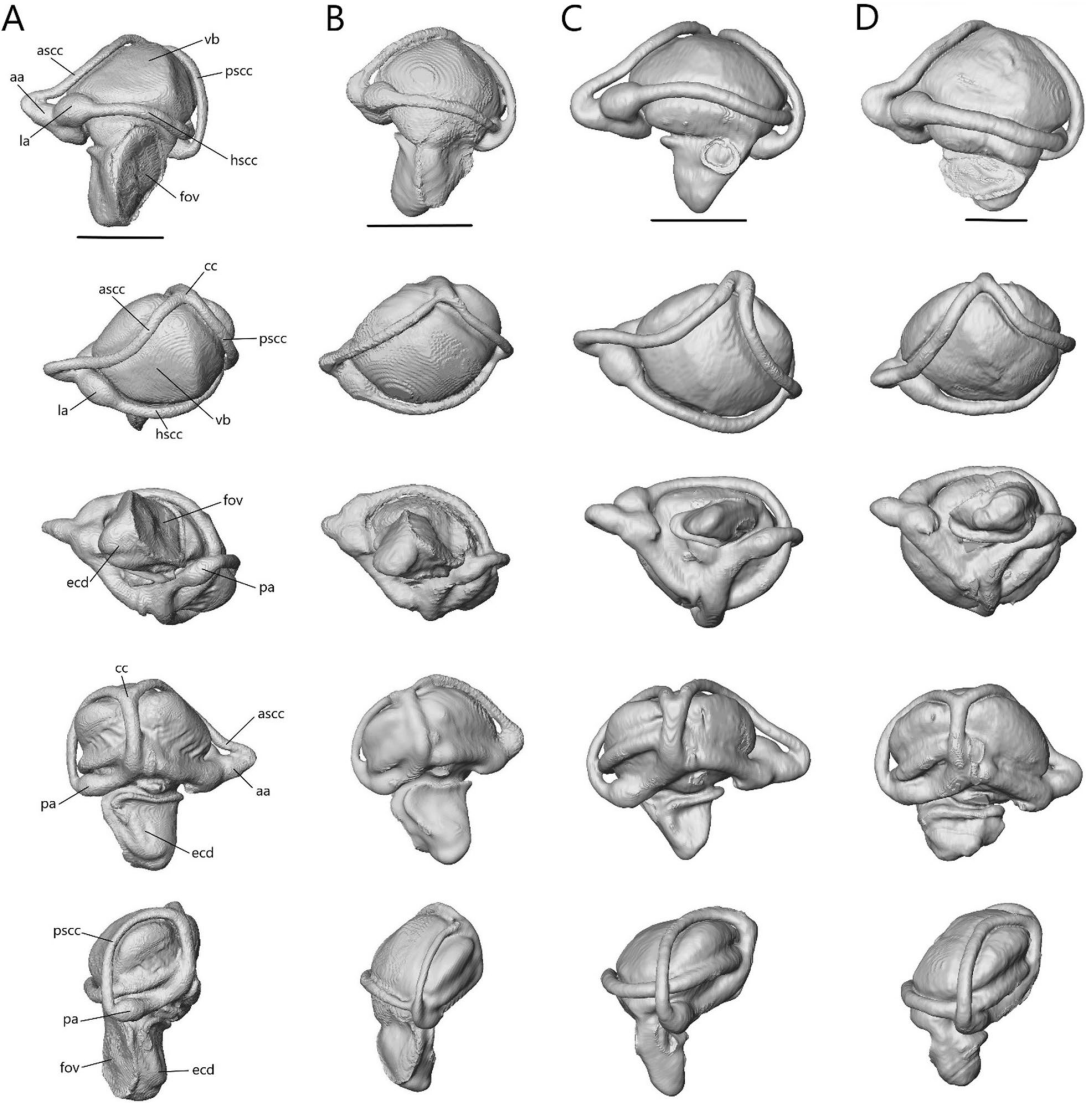
Endocast of inner ear of **A** *T. brevipes*; **B** *T. gracilis*; **C** *S. alberti*; **D** *A. occidentalis*; in lateral, dorsal, ventral, medial, and posterior views from top to bottom. aa, anterior ampulla; ascc, anterior semicircular canal; cc, common crus; ecd, endosseous cochlear duct; fov, fenestra ovalis; hscc, horizontal semicircular canal; la, lateral ampulla; pa, posterior ampulla; pscc, posterior semicircular canal; vb, vestibule. Scale bars = 1 mm

The osseous labyrinth (Fig. 29, or inner ear endocast), which corresponds to the inner ear anatomy, is enclosed within the prootics and otooccipitals. The body of the inner ear is divided into the vestibule, which is the dorsal bulbous space where the three semicircular canals converge, and the endosseous cochlear duct, which is ventral to the vestibule. The anterior semicircular canal and posterior semicircular canal meet dorsomedially to form the common crus. Each semicircular canal expands into a sac called an ampulla. The fenestra ovalis is the opening in which the stapes sits. It opens on the lateral side of the endosseous cochlear duct.

The vestibule is rounded in lateral view and only a little longer than tall in *Typhlacontias* (Fig. 29A, B). The fenestra ovalis is very large and opens on to the ventral region of the vestibule. The horizontal semicircular canal wraps around the lateral side of the vestibule, with nearly no space between them. The anterior semicircular canal follows the curvature of the vestibule for most of its length until it curves out before it joins the anterior ampulla. This curve is more pronounced in *T. brevipes* than in *T. gracilis*. The posterior semicircular canal is similar, following the vestibule closely for most of its length up to the posterior ampulla, where it curves out. There is a larger gap between the posterior semicircular canal and the vestibule in *T. brevipes*. Each of the semicircular canals is tubular with a constant diameter. The anterior ampulla and lateral ampulla are in contact with each other at the anterior side of the vestibule. The posterior ampulla is located on the posterior side.

The vestibule of *S. alberti* (Fig. 29C) is more oblong than in *Typhlacontias*. It is not as enlarged and does not contact the semicircular canals as much as in the more fossorial skinks. The fenestra ovalis is a small, circular opening. It opens to the endosseous cochlear duct, which is similar in shape across the scincines. The arrangement of the semicircular canals is similar, but they are larger. As in *T. brevipes*, the anterior semicircular canal curves and flattens out near the anterior ampulla. The ampullae are also larger, with the lateral ampulla the largest of the three.

The osseous labyrinth of *A. occidentalis* (Fig. 29D) is relatively broader than in the other species. The vestibule is larger and nearly circular whereas the endosseous cochlear duct is shorter and stumpy in comparison. The fenestra ovalis is large as in *Typhlacontias* but oriented laterally rather than posterolaterally. The semicircular canals are thick and most similar to *S. alberti* in appearance. One difference is that the anterior semicircular canal remains in contact with the vestibule for its entire length. Additionally, the anterior and posterior ampullae are smaller than in *S. alberti*, with the posterior ampulla hard to distinguish. The lateral ampulla is of a similar size.

#### Mandibular bones

##### Dentary

**Fig. 30:**
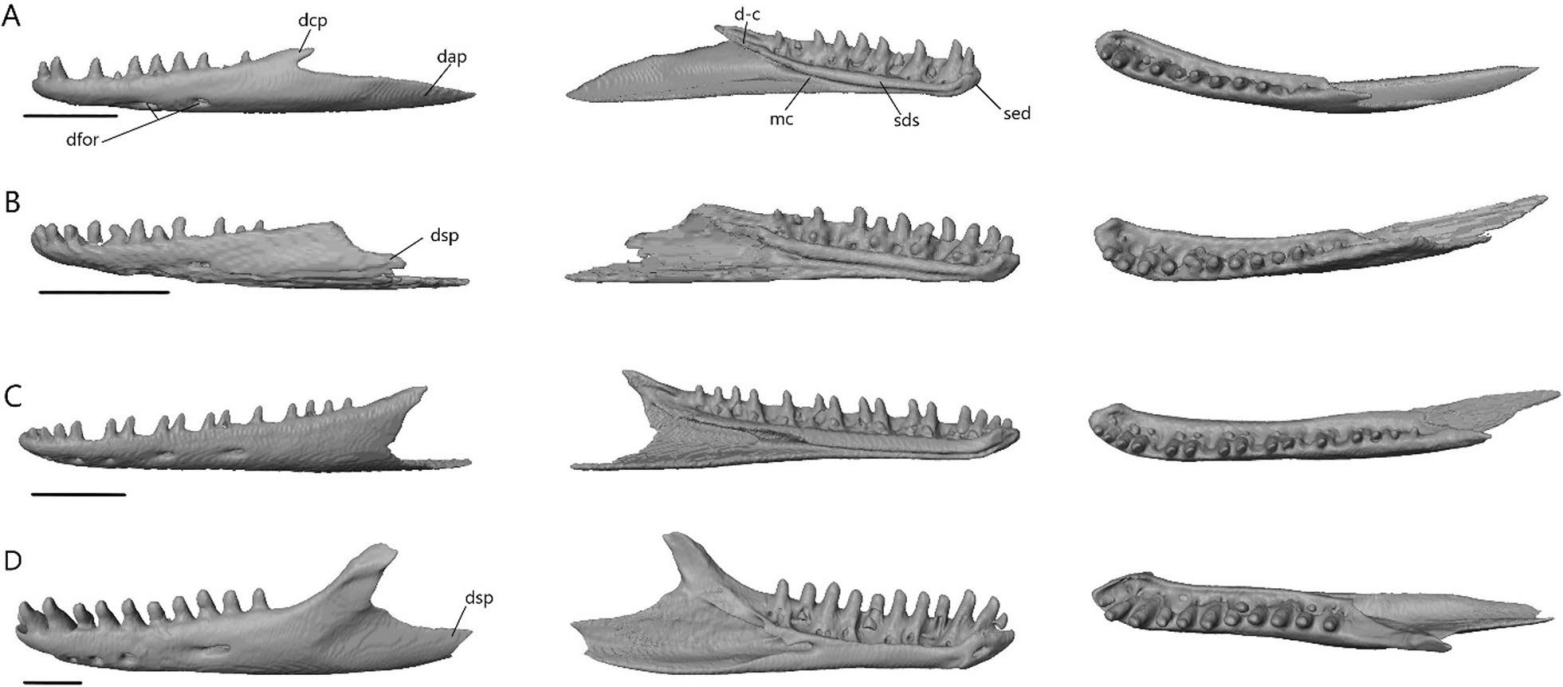
Isolated left dentary of **A** *T. brevipes*; **B** *T. gracilis*; **C** *S. alberti*; **D** *A. occidentalis* in lateral, medial, and dorsal views from left to right. d-c, coronoid facet of the dentary; dap, angular process of the dentary; dcp, coronoid process of the dentary; dfor, mental foramina; dsp, surangular process of the dentary; mc, Meckelian canal; sds, subdental shelf; sed, symphyseal edge. Scale bars = 1 mm

The dentary (Fig. 30) is a long, paired bone that is the most anterior bone of the lower jaw. It contacts the other dentary anteriorly, coronoid and surangular posteriorly, and angular and splenial medially. It houses the Meckelian cartilage in the Meckelian canal and is the tooth-bearing element of the lower jaw.

The dentary has three mental foramina in *Typhlacontias*, two of which are seen laterally and one which is located anteriorly on the ventral side (Fig. 30A, B). The symphyseal edge slants inward and is the contact site for the other dentary. The Meckelian canal runs open along the lingual side. It is shadowed by the subdental shelf, a well-defined ridge that supports the tooth loci. Where the subdental shelf ends, there is a coronoid facet where the coronoid connects. Posteriorly, the dentary of *T. brevipes* bifurcates into the dorsal coronoid process and ventral angular process. The dentary of *T. gracilis* also has these processes, but there is a third process between them, the surangular process. The coronoid process is the shortest one, and contacts the coronoid. It is highly reduced in *T. gracilis* and appears like a blunt corner. In *T. brevipes*, the coronoid process is narrow and ends in a rounded point. The surangular process in *T. gracilis* is rounded and ends at the anterior foramen of the surangular. The angular process is the longest and stretches along the ventral side of the surangular. It also contacts the splenial and angular. The dentaries have 11 and 11 tooth loci (18 teeth present) in *T. brevipes* and 12 and 12 tooth loci (16 teeth present) in *T. gracilis*. The crowns of *T. brevipes* are slightly more pointed compared to *T. gracilis*.

The dentary of *S. alberti* has six mental foramina along its lateral side, four smaller ones evenly spaced along the anterior quarter of the bone and two larger ones posterior (Fig. 30C). The symphyseal edge is roughly the same size as in *Typhlacontias*. The Meckelian canal runs open along the lingual side. The coronoid process is triangular and relatively larger than in *Typhlacontias*. The angular process is triangular and elongate. It covers a portion of the angular, and contacts the surangular and splenial. The dentaries have 20 and 22 tooth loci (30 teeth present). The crowns are rounded.

Overall, the dentary of *A. occidentalis* is more robust than that of the other skinks (Fig. 30D). The dentary has five mental foramina on the lateral side, four evenly-sized ones on the first quarter and one larger, oval-shaped one posterior to them. The symphyseal edge is relatively larger and has a foramen. The Meckelian canal is enclosed. It has an oval opening through which the anterior process of the splenial runs. The anterior process of the coronoid fits into the space created by the ventrally curved subdental shelf. Compared to the other species, the coronoid process is much larger. It is triangular. *Acontias occidentalis* differs from the others in that the most posteriorly-extended point of the dentary is on the lateral side of the jaw. The surangular process extends horizontally to this point and then curves ventrally to form a second smaller point. It contacts the splenial, angular, and surangular. The dentaries have 11 and 12 tooth loci (23 teeth present). These teeth are similar in shape to *Typhlacontias*, but larger with more rounded crowns. Additionally, the second and third teeth are much larger than the other teeth and more pointed.

##### Compound bone (articular and surangular)

**Fig. 31:**
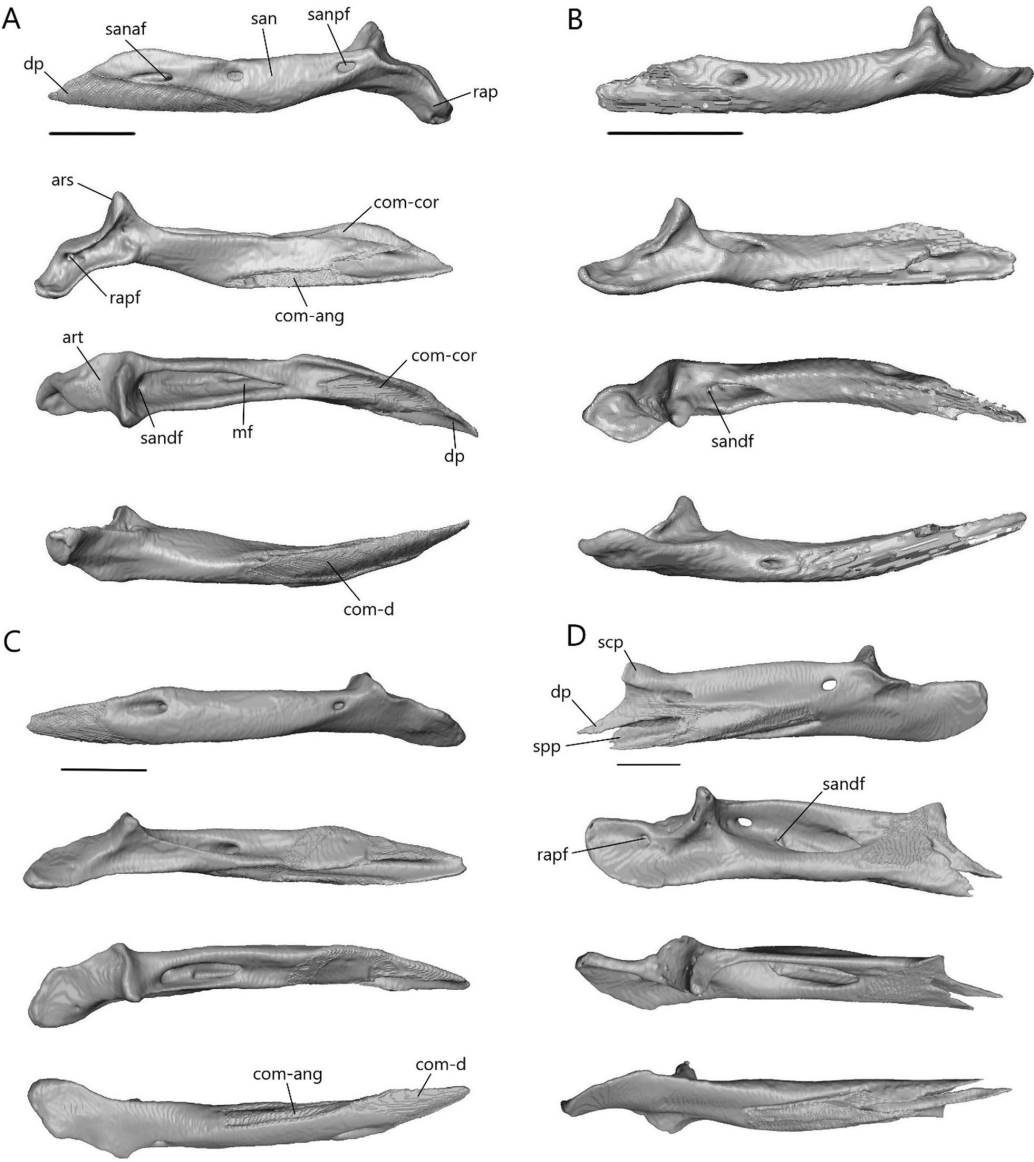
Isolated left compound bone (articular and surangular) of **A** *T. brevipes*; **B** *T. gracilis*; **C** *S. alberti*; **D** *A. occidentalis*; in lateral, medial, dorsal, and ventral views from top to bottom. ars, articular surface; art, articular; com-ang, angular facet of the compound bone; com-cor, coronoid facet of the compound bone; com-d, dentary facet of the compound bone; dp, dentary process of the compound bone; mf, mandibular fossa; rap, retroarticular process; rapf, retroarticular process foramen; san, surangular; sanaf, anterior foramen of the surangular; sandf, dorsal foramen of the surangular; sanpf, posterior foramen of the surangular; scp, coronoid process of the surangular; spp, splenial process of the surangular. Scale bars = 1 mm

The articular and surangular have fused to form a single compound bone (Fig. 31). The surangular is the larger of the two and houses the posterior portion of the Meckelian cartilage. It contacts the dentary anterolaterally, coronoid medially, and splenial and angular ventromedially. It also contacts the angular laterally in *A. occidentalis*. The articular contacts the surangular anteroventrally and the quadrate dorsally. It is the only splanchonocranial element in the mandible.

On its lateral side, the surangular has an anterior foramen and a posterior foramen in *Typhlacontias* (Fig. 31A, B). There is a third foramen located between them in *T. brevipes*. Dorsally, the surangular has the teardrop-shaped mandibular fossa. The mandibular fossa is larger in *T. brevipes*, stretching nearly to where the coronoid sits on the surangular. There is a small dorsal foramen at the posterior side of the mandibular fossa. Anteriorly, the surangular forms a broad dentary process, which contacts the dentary laterally. The tip is pointed in *T. brevipes* and rounded in *T. gracilis*. The coronoid facet is located dorsally on the medial side. Ventral to this, there is an angular facet. The articular surface curves posteroventrally to articulate with the quadrate. It is broader in *T. brevipes*, accommodating the larger quadrate. The retroarticular process extends past the articular surface and flattens into a concave bowl. The retroarticular process of *T. gracilis* is larger and flattens out immediately whereas the retroarticular process of *T. brevipes* slants downward. There is a small foramen beneath the articular surface in *T. brevipes*.

The surangular of *S. alberti* has a larger anterior foramen and a smaller posterior foramen on its lateral side (Fig. 31C). On its dorsomedial side, there is an ovoid mandibular fossa. It is relatively smaller than in *T. brevipes*, but larger than in *T. gracilis*. The posterior foramen opens into the mandibular fossa, as in *Typhlacontias*, but *S. alberti* lacks a dorsal foramen. Anteriorly, the surangular has a broad triangular dentary process, which contacts the dentary laterally and splenial medially. The coronoid facet is located dorsally and is U-shaped. The angular facet begins on the medial side and continues along the ventral side, ending past the middle of the compound bone. The articular surface is not as prominent as in *Typhlacontias*. The retroarticular process flattens out as in *Typhlacontias*, but does not form a concave surface. It is broad and oriented more horizontally than in the other species.

The compound bone of *A. occidentalis* is squatter and has a different anterior shape compared to the scincines (Fig. 31D). There are two foramina on its lateral side, with the anterior considerably smaller than the posterior. Dorsomedially, the surangular has a large, ovoid mandibular fossa. It extends nearly to the coronoid facet. The mandibular fossa is most visible medially in *A. occidentalis*, whereas in *Typhlacontias*, it is closed along its medial side, especially in *T. brevipes*. There is a small dorsal foramen in the mandibular fossa. Anteriorly, the surangular splits into two processes: the dorsolateral dentary process and the ventral splenial process. The dentary process is narrow and triangular. The splenial process is broader. The Meckelian canal runs between them to the mandibular fossa. The surangular has a distinct coronoid process extending from the dorsal side. It is broad and subrectangular, and contacts the coronoid medially and nearly contacts the dentary anteriorly. The coronoid facet is located on the medial side and covers a broad area from the coronoid process to the mandibular fossa. The angular facet is located almost entirely on the ventral side. The articular is not entirely fused to the surangular with a suture visible on the medial side. The articular surface is smaller than the articular surface in the other species. The retroarticular process is broad and flat with a slightly concave medial surface. It is oriented vertically. A small foramen is present directly posterior to the articular surface.

##### Coronoid

**Fig. 32:**
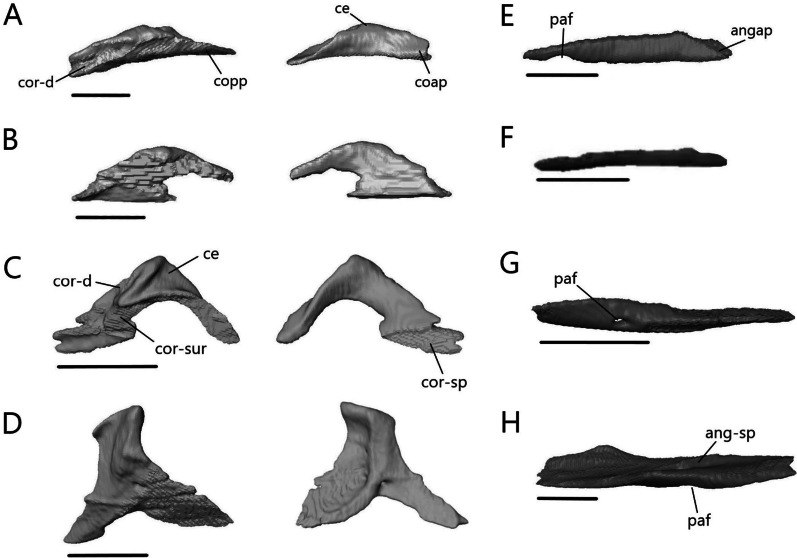
Isolated coronoid and angular. Left coronoid of **A** *T. brevipes*; **B** *T. gracilis*; **C** *S. alberti*; **D** *A. occidentalis*; in lateral (left) and medial (right) views. Left angular of **E** *T. brevipes*; **F** *T. gracilis*; **G** *S. alberti*; **H** *A. occidentalis*; in medial view. ang-sp, splenial facet of the angular; angap, anterior process of the angular; ce, coronoid eminence; coap, anterior process of the coronoid; copp, posterior process of the coronoid; cor-d, dentary facet of the coronoid; cor-sp, splenial facet of the coronoid; cor-sur, surangular facet of the coronoid; paf, posterior alveolar foramen. Scale bars = 0.5 mm

The coronoid (Fig. 32A–D) is a paired bone set near the midpoint of the lower jaw at the boundary between the dentary and surangular. It contacts the dentary anterolaterally, surangular posteroventrally, and splenial anteroventrally.

The coronoid eminence is reduced in *Typhlacontias*, forming a small, gently sloping peak (Fig. 32A, B). The anterior process extends along the lingual side of the jaw and terminates where the subdental shelf begins. On its lateral side, it has a facet which articulates with the coronoid process of the dentary. The posterior process runs along the dorsal side of the surangular. It is flattened, particularly in *T. brevipes*, and ends in a rounded point. The coronoid of *T. brevipes* is flat with only mild curvature along its ventral side whereas the coronoid of *T. gracilis* is arced and shaped more like a sickle.

The coronoid of *S. alberti* is far more pronounced than in *Typhlacontias*. It is a V-shaped bone with a ridge along its lateral side (Fig. 32C). The coronoid eminence forms a triangular peak. The anterior process expands anteroventrally to the subdental shelf. Its lateral side has two facets: the anterodorsal dentary facet and the ventral surangular facet. Its medial side has a large facet for the splenial. The posterior process is as long as the anterior, but narrower.

The coronoid of *A. occidentalis* is the largest of the four species and has a distinct triradiate shape (Fig. 32D). The coronoid eminence is large and trapezoidal with a posterior curvature. Most of its lateral surface is overlapped by the dentary, leaving only a small fraction visible laterally in the lower jaw (Fig. 7D). The anterior process is the smallest of the three processes and extends to the subdental shelf. The posterior process is longer and broader than the anterior process, extending nearly to the mandibular fossa.

##### Angular

The angular (Fig. 32E–H) is a blade-like paired bone mainly located on the medial side of the lower jaw, although it extends on to the ventral and lateral side in some species. It contacts the surangular dorsally and ventrally, dentary ventrally, and splenial anteriorly. In *A. occidentalis*, the contact with the splenial is dorsal.

In ventral view of the mandible (Fig. 7A, B), a small portion of the angular is visible overlapping the surangular in *Typhlacontias*. The posterior alveolar foramen is incomplete in *T. brevipes* (Fig. 32E), with only the dorsal margin present to form a half-circle, and absent in *T. gracilis* (Fig. 32F). Although the angular is elongate and narrow in both species, the anterior process shows more expansion in *T. brevipes* than in *T. gracilis*. This process is slightly overlapped by the splenial in *T. brevipes*.

The angular of *S. alberti* (Fig. 32G) is larger and more robust than in *Typhlacontias*. It extends along the ventral side of the mandible (Fig. 7C), overlapping the surangular. The posterior alveolar foramen is present at roughly two-thirds of the length of the bone. The anterior process is elongate and narrows to a point. It is overlapped slightly by the splenial.

The angular of *A. occidentalis* (Fig. 32H) is the largest and most complex of the examined species. It wraps around the ventral side of the mandible and extends on to the lateral side. Its end is broad and rounded. The posterior alveolar foramen is located on the ventral side. It is overlapped by the dentary. The anterior process is large and rectangular with only a slight narrowing towards its end. There is a distinct splenial facet on the dorsal side of the anterior process.

##### Splenial

**Fig. 33:**
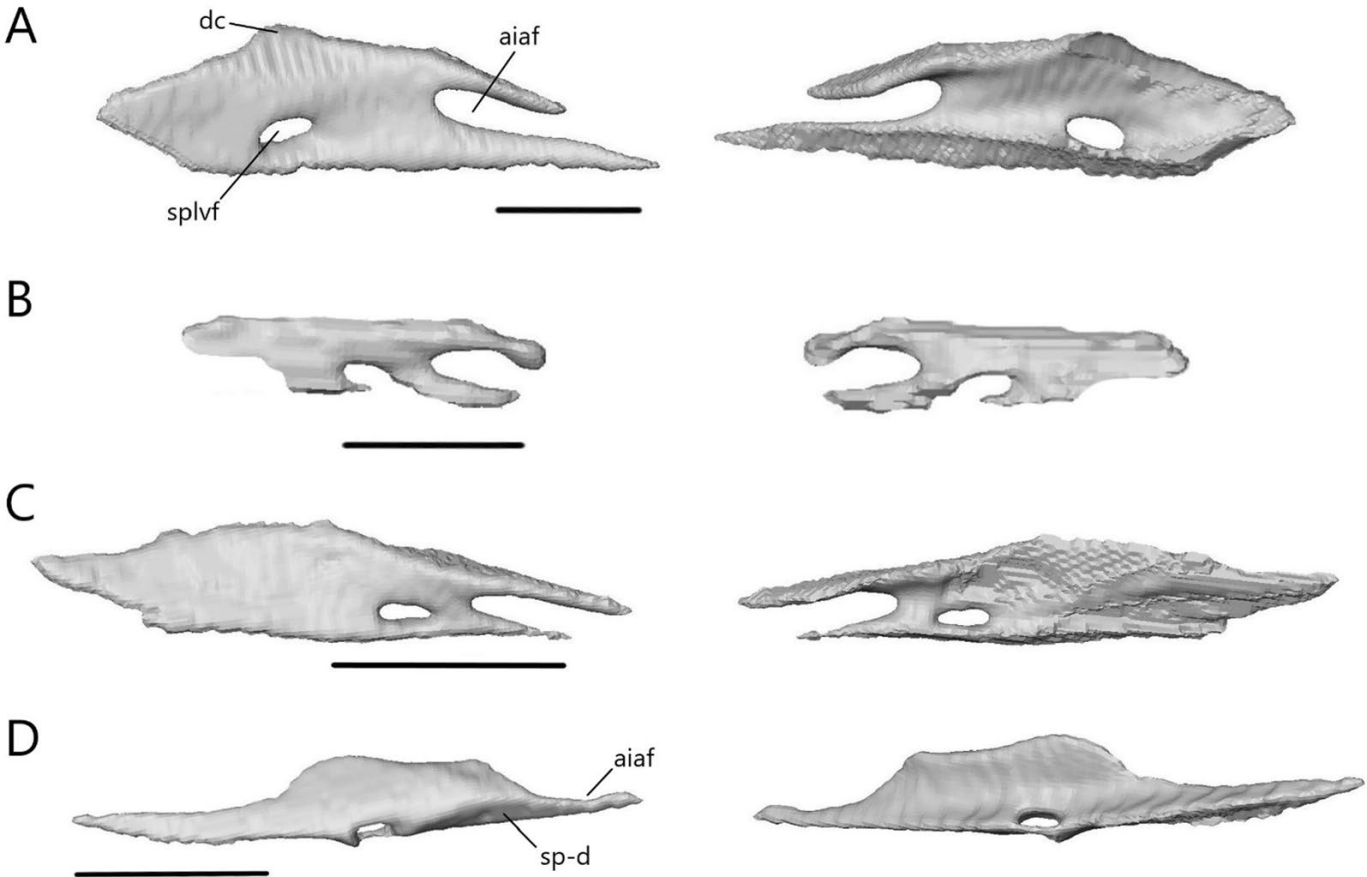
Isolated left splenial of **A** *T. brevipes*; **B** *T. gracilis*; **C** *S. alberti*; **D** *A. occidentalis*; in medial (left) and lateral (right) views. aiaf, anterior inferior alveolar foramen; dc, dorsal crest; sp-d, dentary facet of the splenial; splvf, ventral foramen of the splenial. Scale bars = 0.5 mm

The splenial (Fig. 33) is a flattened, paired bone located on the medial side of the lower jaw. It contacts the dentary anteriorly, coronoid dorsally (except in *A. occidentalis*), angular posteriorly and ventrally, and surangular posteromedially. It encloses the Meckelian canal.

Anteriorly, there are two elongate processes which partially enclose the anterior inferior alveolar foramen in *Typhlacontias*. In *T. brevipes*, the dorsal process is shorter than the ventral one (Fig. 33A) whereas the two processes are nearly of equal length in *T. gracilis* (Fig. 33B). Posterior to this, there is a smaller foramen for the anterior mylohyoid nerve. This foramen is closed in *T. brevipes* and open ventrally in *T. gracilis*. There is a slight dorsal crest in *T. brevipes*. The splenial is broader and more robust in *T. brevipes* than in *T. gracilis*.

The splenial of *S. alberti* is relatively larger than in *Typhlacontias*, particularly its expanded posterior end (Fig. 33C). The anterior side bifurcates into two processes, of which the dorsal process is the larger. They partially enclose the finger-shaped anterior inferior alveolar foramen. Posteriorly, the ventral foramen for the anterior mylohyoid nerve is completely enclosed. It is flattened and more ovoid compared to *Typhlacontias*. It is also very close to the anterior inferior alveolar foramen, similar to *T. gracilis*. The posterior end is much larger and narrows to a triangular point. The dorsal edge is relatively straight.

The splenial of *A. occidentalis* (Fig. 33D) is quite different in shape. It narrows to a slim process that extends between the lateral and medial sides of the dentary (Fig. 7D). The ventromedial side of this process is flattened where it contacts the dentary. Posteriorly, the ventral foramen for the anterior mylohyoid nerve is more ventrally oriented than in the other species. It is entirely enclosed and similar in shape, though smaller, to *S. alberti*. The posterior end has a long, triangular process. The dorsal edge forms a small, rounded crest.

## Discussion

We used geometric morphometrics and comparative anatomy to examine skull evolution in African burrowing skinks. Overall, we found a strong effect of phylogenetic history and a smaller effect of size on skull shape. Degree of limb reduction was also correlated with shape, but this disappeared when phylogeny was accounted for. Substrate played a smaller role, although sand burrowers consistently stood out as different from other burrowers. We also created a detailed anatomical atlas for the skulls of four skink species, none of which have been examined in detail and three of which come from genera that have never been the subject of dedicated osteological study. Every element showed some degree of variation in shape and relative scaling of features, even within the same genus. Some of the most variable bones included the skull roofing bones, septomaxilla, vomer, and palatine. The epipterygoid and bones of the braincase showed relatively less variation. We begin by discussing the results from our morphometric analyses with a focus on the main variables, and then examine major anatomical differences and similarities between four species in light of phylogenetic relationships and burrowing specialization. We also show how broad-scale similarities in traits can be accomplished through different anatomical changes.

### Allometry

Differences in allometric scaling between subfamilies and genera likely contributed to the variation in shape. For instance, whereas larger acontines have an hourglass-shaped parietal, larger scincines tend to have an almost square parietal. Furthermore, *Acontias* has the largest variation in skull size (6.98 mm in *A. gariepensis* to 31.00 mm in *A. plumbeus*) and showed the greatest spread in morphospace. This distribution roughly aligned with size, underlining how variation in size can correspond to variation in shape.

Allometric scaling often has important functional implications, including changes to retain equivalent functionality [55, 56]. The relative increase in the size of the braincase in smaller species observed here may be related to an increase in the relative size of the semicircular canals. The inner ear must stay above a certain size to remain functional and a corresponding change to the relative size of the braincase is common in miniaturized species [57]. This change in proportion can also explain the shift in the quadrate’s relative position; as the braincase expands in relative size, it closes the distance between itself and the quadrate.

Whereas some of the allometric shifts in smaller species are likely related to maintaining sensory function, allometric shifts in larger species are likely related to other functions. Fossorial lizards often show a trade-off between burrowing speed and bite force, as both are related to relative head width [15]. However, in some groups, no difference in bite force is found despite the predicted effects of head shape, implying compensatory changes to preserve similar performance [16, 58, 59]. In larger species of *Amphisbaenia* and *Leposternon*, the skull is hourglass-shaped, which may allow an increase in adductor muscle mass [31, 60]. The hourglass-shaped parietal in large *Acontias* may similarly provide greater space for the adductors and increase bite force while keeping the head narrow for efficient burrowing. In contrast, *Feylinia* have a square parietal. *Feylinia* inhabit leaf litter [61], a looser substrate where head width may not limit burrowing as much or at all compared to the denser substrates *Acontias* is found in. Soft tissue studies and performance tests are required to test these hypotheses.

### Phylogenetic history

Phylogenetic history has a strong relationship with cranial and mandibular shape in African burrowing skinks with genus being the variable that most explains variation in shape. The pattern is not surprising as phylogenetic history is important in shaping skull morphology in many clades including amphisbaenians [18], geckos [62], gymnophthalmids [23], varanids [20], and across lizard families generally [17]. Despite the clear differentiation between most genera in morphospace, phylogenetic signal was lower than expected. This may be the result of the overlap between *Scelotes* and *Sepsina*, *Feylinia* and *Typhlacontias* having a specialized morphology closer to acontines, and shifts in morphology within genera due to other factors.

*Scelotes* and *Sepsina* show the most overlap in morphospace. This is seen even though *Sepsina* is more closely related to *Typhlacontias* and *Feylinia* than to *Scelotes*. The likely explanation is that *Scelotes* and *Sepsina* share an ancestral and conserved scincine morphology. Although *Sepsina*’s proximity to *Scelotes* impacts the low phylogenetic signal, the effect is likely diluted as the current phylogeny only includes one species of *Sepsina* and is missing four *Scelotes*. Inclusion of these species would likely contribute to the low phylogenetic signal.

Degree of specialization for burrowing also seems to play a role in explaining skull morphology, with more specialized burrowers having depressed skulls, relatively long and pointed snouts, shorter frontals, and fewer teeth. Other qualitative traits not captured by the morphometrics are described later. Many of these traits have previously been reported in other burrowing squamates [24, 27]. Acontines show the most morphological specialization and are considered the most fossorial. *Typhlacontias* and *Feylinia*, both mostly limbless and highly fossorial, show convergence with these acontine morphologies and are found in-between them and the less specialized scincines in morphospace.

Finally, morphological shifts within genera that do not reflect phylogenetic history and are the result of changes in size or substrate may also play a role in explaining why phylogenetic signal is low. For example, large body size and its associated traits (like the hourglass-shaped parietal) evolved multiple times in *Acontias*, leading to convergence between non-sister species.

### Limb reduction

We found a strong relationship between the number of limbs and digits with skull morphology, but the relationship disappeared when phylogeny was taken into account. Most likely, it was driven by the large number of genera that only exhibit one limb state, especially the acontines, which are all limbless. However, limbless skinks are considered the most specialized for burrowing [51], so it follows that they would have different skull morphologies compared to two and four-limbed skinks. This trend can be observed by examining the anatomy more closely.

When looking at qualitative traits, differences are seen between *Scelotes* possessing different numbers of limbs. Some of the changes observed in some of the two-limbed and all limbless *Scelotes* compared to the four-limbed species include an increase in the size of the cristae cranii, reduction of the squamosal, increase in the fenestra ovalis, and reduction of the tympanic crest. *Scelotes arenicola*, a limbless skink found in sandy soil, has some of the most specialized traits among *Scelotes* including tube-like cristae cranii, orbitonasal flanges that extend to the midline, larger stapes, thin squamosal, elongation of the snout, and general flattening of the skull. These traits are similar to those observed in *Typhlacontias* and *Acontias*, whose anatomy we described in greater detail, and point to a potential progression in skull specialization that accompanies limb reduction.

### Substrate and burrowing specialization

Compared to other studies on other burrowing lizards [23, 31], we found a less clear relationship between skull shape and substrate. Our results do concord with a study on amphisbaenians [18], which suggested that skull morphology is largely shaped by phylogenetic history, not soil type, due to strong stabilizing selection for burrowing. Although phylogenetic history is key in explaining skull variation in African burrowing skinks, there is notable convergence between sand-burrowing species of different genera.

Sand burrowers, including species in the genera *Acontias*, *Typhlosaurus*, and *Typhlacontias*, have a unique morphology characterized by long and narrow skulls, wedge-shaped crania with flattened skull roofs, long snouts, relatively long braincases, small suborbital fenestrae, and longer dentaries with short tooth rows. Sand is a complex substrate that possesses both solid and liquid properties, is composed of smaller grain sizes, and is generally unstable [63, 64]. Given its unique physical properties, it follows that lizards would require unique adaptations to burrow through it. Some of these adaptations, including a wedge-shaped head and long snout, are also seen in limbed lizards that dive into sand to escape predators [16, 64, 65]. A wedge shape may help to better part the sand. Many sand burrowers also specialize on termites [66, 67], which may have influenced the unique mandibular shape, as seen in non-fossorial myrmecophagous lizards [68]. Such a relationship could not be directly tested here because of the paucity of dietary information in burrowing skinks (although, some are considered termite specialists; [67]). The variation seen between sand burrowing species is primarily the result of phylogenetic history and secondarily size, but within genera, there may also be variation caused by fine-scale differences in sandy habitats. For instance, within *Typhlacontias*, species may inhabit dunes, vegetated sandy areas, and areas with higher rainfall [69]. Each of these microhabitats is likely to present different physical properties and challenges, which may require different adaptations.

One caveat to this study is that the full dataset could not be analyzed using phylogenetic comparative methods. The reduced dataset showed some variation from the full dataset in the results, even before phylogenetic correction, so the reduction clearly had an impact. Only scincines were removed and there was a disproportionate effect on substrate representation with the following left: leaf litter (4/8), sand (10/12), sandy soil (10/13), and soil (4/5). The sharp reduction of the leaf litter group may explain the loss of some significant differences in the phylogenetic dataset. A fully resolved phylogeny is needed to analyze the full dataset. That said, the difference observed between sand burrowers and other burrowers is likely to remain significant.

Another limitation, shared by many studies looking at the relationship between substrate and morphology [3, 18, 23, 31], is in substrate characterization. The most accurate way would be to measure mechanical properties of the substrate as specimens are collected. This is logistically challenging as morphological studies tend to use museum specimens, which lack environmental data. Further complicating matters is that the degree of substrate compaction likely varies across ranges and temporally based on soil moisture, vegetation, and other variables. We characterized substrate using anecdotal field observations, condensing them into one of four broad categories. These categories captured large variation between substrates (e.g., leaf litter is loose and relatively easy to move through, sand is composed of smaller particles, etc.), but likely missed out on fine-scale physical differences. Other studies use location and associated data (bioclimatic factors, soil type) to study the relationship between substrate and morphology [18, 31]. This works well when species are all burrowing in soil as with amphisbaenians. However, this method does not work for our system where many species occupy leaf litter rather than the soil itself. In the future, studies may seek to measure substrate properties and test burrowing performance in the field to more closely test the relationship between substrate, performance, and morphology.

### Anatomical comparisons across phylogenetic levels

No complete anatomical descriptions exist for *Typhlacontias* to compare with the work in this study, although most diagnostic features [70] are reported here. Previously, *Typhlacontias* have been regarded as lacking jugals [70]. We found reduced jugals in both species, highlighting the value of CT scans in recognizing small structures that may be lost in a skeletal preparation.

The skulls of *T. brevipes* and *T. gracilis*, although more similar to one another than to the other species, show variation both in general skull shape and in each individual bone. Some of these differences are related to fossorial adaptations, with *T. brevipes* showing larger orbitonasal flanges, more robust cristae cranii, smaller suborbital fenestrae, and a more closed palate compared to *T. gracilis*. These traits seem to indicate a higher degree of specialization in *T. brevipes*. However, *T. gracilis* has a larger crista alaris and smaller posttemporal fenestrae. Furthermore, *T. brevipes* retains rudimentary hindlimbs. These complex combinations of traits indicate that even within a single genus, the evolution of burrowing adaptations does not follow a single linear path. Some of these differences may also be related to differences in substrate or environmental conditions. Both species are described as sand-swimmers, but *T. brevipes* occupies more vegetated sandy environments whereas *T. gracilis* is found in Kalahari sand in areas with greater annual rainfall [69], which may impact the physical properties of the sand.

Another source of variation is the development of the epipterygoid. *Typhlacontias gracilis* retains a prominent epipterygoid whereas *T. brevipes* has a small epipterygoid. It is unclear if this is because it is partly cartilaginous, or if it has been greatly reduced. Furthermore, a bony epipterygoid is absent in *T. punctatissimus*. This may indicate a reduction of the epipterygoid in *Typhlacontias*. Reduction and loss of the epipterygoid would be novel in skinks, but has been seen in other lizards [14]. Clearing-and-staining to visualize cartilage (if present) and larger number of specimens to look at intraspecific variation is required before definite conclusions can be drawn.

There are no previous detailed osteological studies of *Sepsina* to compare our study with. Greer [70] noted the following diagnostic skull features: wide separation of palatines, medially expanded palatine process of the pterygoid, pterygoid teeth, postorbital and upper temporal arch, and 12–18 teeth per maxilla. All these are observed here in *S. alberti*.

Many of the bones are similar in shape between *S. alberti* and *Typhlacontias* and in some cases, more similar between *S. alberti* and one of the *Typhlacontias* than between the two *Typhlacontias*. For example, the parietal of *T. gracilis* is more similar to *S. alberti* due to the development of a complex parietal fossa and bifurcated posterior processes in *T. brevipes*. Such similarities can likely be attributed to conserved anatomy. *Sepsina alberti* also shows many differences to *Typhlacontias*. Of the examined skinks, *S. alberti* is noteworthy in having postorbitals, interparietals, palpebrals, pterygoid teeth, and a highly ossified hyoid apparatus. Other differences include teeth extending up to the posterior process in the maxilla (also seen in *A. occidentalis*), shorter orbitonasal flanges, relatively longer frontal, more open palate, wider palatine process of the pterygoid, taller coronoid, larger angular, and the quadrate, which resembles the more typical conch shape and retains a prominent tympanic crest.

The skull anatomy of *A. occidentalis* varies the most from the other species despite some convergence with *Typhlacontias*. Some key differences include: the closed Meckelian canal, large anteromedial premaxillary process of the nasal, enlarged anterolateral process of the frontal, greater development of the cristae cranii, hourglass shape of the parietal with expanded descending processes, reduced squamosal, decreased size of vomeronasal cavity relative to septomaxilla, flattened supratemporal, presence of lateral process of the palatine, absence of occipital recess and crista interfenestralis, large coronoid process of the dentary, and bifurcated anterior side of the surangular. Some of these traits are likely related to increased burrowing specialization or other functional aspects (such as the supratemporal, which supports the quadrate) in *A. occidentalis* whereas others to phylogenetic history.

*Acontias occidentalis* shares many of the typical acontine features including a scroll-like air passage formed by the palatines, lateral process of the palatine, robust cristae cranii, broad supratemporal, large crista alaris, overlap of the maxilla by the vomer, and exclusion of the frontal from the dorsal orbital margin by the prefrontal and postfrontal [10, 43–45]. The frontals also have anterolateral processes, which are seen in most acontines [10]. These processes vary in size between species and *A. occidentalis* has relatively large anterolateral processes. Like many *Acontias*, *A. occidentalis* has lost the anterior process of the jugal and the upper temporal arch through the reduction of the squamosal. The jugal is not as reduced as in some species where it becomes a stub or absent altogether [10]. The squamosal is very reduced. The lateral wall of the skull is not as closed in *A. occidentalis* as in some species due to a relatively narrow crista alaris and smaller parietal downgrowth. More unique features include the triradiate checkmark shape of the postfrontal (shared with *Acontias meleagris* and *Acontias namaquensis*), hourglass shape of the parietal (shared with *Acontias gracilicauda*, *A. namaquensis*, and *A. plumbeus*), roughly triangular quadrate, and elliptical stapes with narrow, constricted shaft (most closely resembles *A. gracilicauda*). Many of these features appear multiple times across the acontine phylogeny.

### Fossorial adaptations in the skull

All four skinks show skull modifications seen in other fossorial squamates, although the degree to which these are expressed varies, especially between *S. alberti* and the more specialized burrowers. These modifications include a skull that is twice as long as it is wide, parietal downgrowths, closure of the lateral wall of the cranium, reduction of the posttemporal fenestrae, and interdigitated frontoparietal suture. More adaptations are seen just in *Typhlacontias* and *A. occidentalis* including loss of the upper temporal fenestrae, closure of the medial wall of the orbit, development of the cristae cranii, closure of the palate, larger occipital condyle and footplate of the stapes, reduction of the jugal and tympanic crest, and enlarged vestibular structures in the inner ear [11]. *Acontias occidentalis* also has large basicranial sesamoids. Basicranial sesamoids are found in many squamate families, including Scincidae, although previous examination of an acontine did not find any [6, 71]. They are typically small in non-fossorial squamates, but can be large in burrowers to provide a larger attachment site for the longus colli muscle [53, 71]. A larger longus colli muscle, which pulls the head down, may allow for the generation of greater forces for burrowing. *Typhlacontias* lack basicranial sesamoids, but the enlarged sphenooccipital tubercles may offer a similar advantage.

Although many of these traits are broadly convergent, they are often formed through different changes to the anatomy, reflecting phylogenetic history. One such trait is the loss of the upper temporal fenestrae. Like many burrowing squamates [24, 72], *A. occidentalis* loses the upper temporal fenestrae through the reduction of the squamosal. *Typhlacontias* retains a prominent squamosal, but loses the upper temporal fenestra through the close apposition of the squamosal and parietal. Another trait is the closure of the lateral wall of the skull, which helps to strengthen it [24]. In *Typhlacontias*, this is accomplished through the expansion of the crista alaris. In *A. occidentalis*, the crista alaris plays a role in closing the lateral wall, but the expansion of the parietal is more important. Similarly, the closure of the anteromedial region, which strengthens the skull and provides protection for the olfactory tracts, is accomplished in different ways. In *Typhlacontias*, the orbitonasal flanges of the prefrontal are expanded, closing much of the anterior wall. The cristae cranii are large in *T. brevipes* and contribute to the closure of the region, but in *T. gracilis*, they do not play a large role. In *A. occidentalis*, the prefrontal is smaller and the crista cranii takes the prime responsibility in closing the region. The reduction of the jugal is also different. In *Typhlacontias*, the posterior process is reduced and the anterior process remains. In *A. occidentalis*, the anterior process is reduced, resulting in a free-floating, splint-like jugal.

Although skinks have generally been thought to lack cranial kinesis [73], anatomical studies have pointed to the potential for it in some species, including several acontines [10, 44], and experimental work has demonstrated it in a small species [52]. Our work provides further evidence for the potential of mesokinesis in some skinks (closure of upper temporal fenestrae, reduction of jugal and postorbital, gap between cristae cranii and parietal downgrowths permitting movement). While reduction or loss of cranial kinesis may occur when reinforcing the skull, burrowers may also benefit from a mesokinetic skull that can flatten more (suggested as an adaptation in rupiculous lizards; [74]), further streamlining it. Experimental work is needed to confirm whether cranial kinesis is present and if so, whether it differs across substrate types.

## Conclusions

Our work sought to understand the evolution of skull morphology in African burrowing skinks and more broadly, how morphology changes for a highly specialized and demanding lifestyle like burrowing. Phylogenetic history is the greatest factor shaping skull morphology in African burrowing skinks, with size and substrate taking on secondary roles. Although there is broad convergence in both shape and other traits related to burrowing, our anatomical comparisons reveal these convergences are achieved through different anatomical modifications. Our work shows that morphological specialization and convergence can occur through different pathways, even within a single family, adding to the literature on imperfect convergence and lineage-specific constraints [4, 5]. The atlased species also contribute to a growing body of detailed skull morphology literature made possible by CT scanning [11, 39, 53, 75] that will facilitate the characterization of other lizard skulls for phylogenetic and functional studies. By using both qualitative and quantitative methods, we gained a richer understanding of how phylogenetic history, ecology, and functional pressures influence morphology and showed that there are many ways to be a burrower.

Further work may involve sampling burrowing skinks from elsewhere to test whether these patterns hold up across the family. Additionally, functional analyses are necessary to understand the implications of morphological variation and how burrowing differs between species from different genera, of different size, and between different substrate users. Finite element analysis, performance tests, and measurements of substrate hardness in the field can all aid in unraveling if and how different skull morphologies yield different interactions with the environment and may assist in better understanding lineage-specific constraints.

## Methods

### Scanning and segmentation

One adult individual (identified as such by body size) from 39 species was CT scanned at the University of Florida Nanoscale Research Facility in Gainesville, FL. All specimens were vouchered museum specimens, with 30 specimens from the California Academy of Sciences (CAS) in San Francisco, CA, and 9 specimens from the Museum of Comparative Zoology (MCZ) in Cambridge, MA. Most specimens were scanned using a GE V|tome|xm240 CT Scanner with the X-ray source set to 80 kV, 170 µa, and 13.6 W. Four specimens (all *Sepsina*) were scanned earlier using a Phoenix VTome|x M scanner with an X-ray source set to 80 kV, 180 µa, and 14.4 W. Two scans were done per specimen: one full body scan and one region-of-interest scan of the skull to capture finer detail. Scans were reconstructed on GE’s datosjx software. Additional scanning information is available in Additional file 1: Table S17 and all scans are available for download on Morphosource (https://www.morphosource.org/projects/00000C482/).

We digitally segmented each cranium and right mandible from the region-of-interest skull scans in Avizo v.9.4.0 (Visualization Sciences Group, Burlington, Massachusetts, USA). The cranium of *Scelotes caffer* and the mandible of *Scelotes arenicola* were broken and excluded from the cranial and mandibular morphometric analyses respectively. For the comparative anatomy, we digitally segmented each bone as a separate element for four species: *T. brevipes* (CAS 224004), *T. gracilis* (MCZ R-18023), *S. alberti* (CAS 263923), and *A. occidentalis* (CAS 196430). We also segmented the empty space of the osseous labyrinth to form an endocast of the inner ear. Most images were taken in perspective mode with only Figs. 5 and 6 taken in orthographic mode. Lengths and widths of structures were measured using the measure tool in Avizo. When necessary to verify the presence or absence of sutures that may have been smoothed over in the 3D visualization (e.g. suture between two premaxillae), we examined the 2-dimensional tomogram slices. Identification of anatomical structures were based on previous studies of skink osteology [11, 39, 75].

### Classifiers

Data on microhabitat and number of limbs and digits were compiled from the primary literature and field guides [61, 66, 69, 76–80]. We classified species into four broad substrate categories: leaf (for species in leaf litter), sand (for sand swimmers and species living in Kalahari, Namib, coastal, or dune sands), sandy soil (for microhabitats referred to as sandy soil), and soil (for burrowing in compact soil, humus soil, and alluvial soil). *Feylinia elegans* lacked species-specific microhabitat information, but as the other species in the genus are in leaf litter, it was assumed *F. elegans* was too. Limb and digit numbers were confirmed by examinations of the full body CT scans for each specimen. All classifier information is available in Additional file 2.

### Geometric morphometrics

We used 3D geometric morphometrics to characterize variation in the overall shape of the cranium and lower jaw. Fixed landmarks were placed at homologous, repeatable points using the package *geomorph* version 3.0.7 [81] within R version 3.5.1 [82]. Landmark datasets were created and analyzed separately for the cranium and mandible to avoid differences in orientation and position due to specimen preparation (such as if the jaw was open or closed). We used 21 landmarks on the cranium and 13 on the mandible (Fig. 2). Written descriptions of the landmark positions are available for the cranium and mandible (Additional file 1: Tables S18, S19).

### Statistics

Cranial and mandibular landmarks were analyzed separately using *geomorph*. Analyses were run using three different datasets for each set of landmarks. The first dataset contained 38 species and was analyzed using only non-phylogenetic methods. The second contained all species except *Mochlus sundevallii*, which was removed to allow for pairwise comparisons to be run between genera and subfamilies. The third dataset included only the species present on the phylogenetic tree, resulting in a reduced dataset of 28 species. This dataset was analyzed using both phylogenetic comparative methods and non-phylogenetic analyses.

We first performed a Generalized Procrustes analysis (GPA) on the landmarks to align, rotate, and scale them to a common coordinate system and unit-centroid size to remove variation in position, orientation, and size [81, 83]. To visualize shape variation, we performed a principal component analysis (PCA) using the Procrustes coordinates and graphed specimens in two dimensions of tangent space (PC1 and PC2) using the R packages *ggplot2* version 3.1.0 [84] and *ggthemes* version 4.0.1 [85].

For use in the phylogenetic comparative analyses, we pruned a multi-locus molecular squamate phylogeny [41] to include only the taxa in our dataset using the R package *phytools* version 0.6.6 [86] and *geiger* version 2.0.6.1 [87]. We calculated the *K*-statistic’s generalization for multivariate data [88] to determine the phylogenetic signal in our shape data (Procrustes coordinates) using the physignal function in *geomorph*.

To test for a relationship between shape and size, we ran multivariate and phylogenetic regressions between shape (as characterized by the Procrustes coordinates) and centroid size. A homogeneity of slopes (HOS) test and post-hoc pairwise slope comparisons were performed on the dataset excluding *Mochlus* to determine if the genera and subfamilies had diverged in allometric relationships. We ran multiple analysis of covariance (ANCOVA; [89]) and phylogenetic generalized least squares (PGLS; [90]) to test for a relationship between shape and one of the variables (genus, subfamily, number of limbs, number of digits, and substrate) using the Procrustes coordinates, with log-transformed centroid size as the covariate. Post-hoc pairwise comparisons were run to determine which limb states and substrates diverged from one another for all datasets. Post-hoc pairwise comparisons were also run to determine which genera and subfamilies diverged from one another using the dataset excluding *Mochlus*.

## Supplementary Information

**Additional file 1.** Contains 19 additional tables containing results from statistics, scanning information, and written descriptions of landmarks.

**Additional file 2.** Table of classifiers used in data analysis.

## Data Availability

The CT data generated and analyzed are available at Morphosource (https://www.morphosource.org/projects/00000C482).

## Abbreviations

BM: Brownian motion
CT: Computed tomography
SVL: Snout–vent length
