# Supplementary material for: Phylogenetic history influences convergence for a specialized ecology: comparative skull morphology of African burrowing skinks (Squamata; Scincidae)
Source: BMC Ecol Evol. 2021 May 16;21:86. doi: 10.1186/s12862-021-01821-w (PMC8127277; doi:10.1186/s12862-021-01821-w)
Supplement: Supplementary file 1 — Additional file 1. Contains 19 additional tables containing results from statistics, scanning information, and written descriptions of landmarks. [file 12862_2021_1821_MOESM1_ESM.docx]

**Table S1.** Principal component scores with their standard deviations and proportion of variance explained from the PCA performed on the full dataset looking at cranial variation.

| PC score | Standard deviation | Proportion of variance | Total variance explained |
| --- | --- | --- | --- |
| PC1 | 0.07314 | 0.42188 | 0.42188 |
| PC2 | 0.04002 | 0.12629 | 0.54818 |
| PC3 | 0.03669 | 0.10616 | 0.65434 |
| PC4 | 0.03354 | 0.08873 | 0.74307 |
| PC5 | 0.02457 | 0.04762 | 0.79069 |
| PC6 | 0.0229 | 0.04135 | 0.83204 |
| PC7 | 0.01946 | 0.02988 | 0.86192 |
| PC8 | 0.01666 | 0.0219 | 0.88381 |
| PC9 | 0.01484 | 0.01738 | 0.90119 |
| PC10 | 0.01261 | 0.01254 | 0.91373 |
| PC11 | 0.01209 | 0.01152 | 0.92525 |
| PC12 | 0.01162 | 0.01065 | 0.9359 |
| PC13 | 0.01102 | 0.00958 | 0.94547 |
| PC14 | 0.0093 | 0.00682 | 0.95229 |
| PC15 | 0.00896 | 0.00633 | 0.95863 |
| PC16 | 0.00841 | 0.00557 | 0.9642 |
| PC17 | 0.00804 | 0.0051 | 0.9693 |
| PC18 | 0.00768 | 0.00465 | 0.97395 |
| PC19 | 0.00706 | 0.00393 | 0.97788 |
| PC20 | 0.00647 | 0.0033 | 0.98118 |
| PC21 | 0.00615 | 0.00298 | 0.98416 |
| PC22 | 0.0054 | 0.0023 | 0.98646 |
| PC23 | 0.00494 | 0.00192 | 0.98839 |
| PC24 | 0.00459 | 0.00166 | 0.99005 |
| PC25 | 0.00453 | 0.00162 | 0.99166 |
| PC26 | 0.00436 | 0.0015 | 0.99316 |
| PC27 | 0.00403 | 0.00128 | 0.99444 |
| PC28 | 0.0039 | 0.0012 | 0.99564 |
| PC29 | 0.00333 | 0.00087 | 0.99651 |
| PC30 | 0.0032 | 0.0008 | 0.99732 |
| PC31 | 0.00292 | 0.00067 | 0.99799 |
| PC32 | 0.0028 | 0.00062 | 0.99861 |
| PC33 | 0.00239 | 0.00045 | 0.99906 |
| PC34 | 0.00196 | 0.0003 | 0.99936 |
| PC35 | 0.00184 | 0.00027 | 0.99963 |
| PC36 | 0.00155 | 0.00019 | 0.99982 |
| PC37 | 0.00153 | 0.00018 | 1 |

**Table S2**. Pairwise slope comparisons from homogeneity of slopes tests comparing cranial allometry across genera, excluding *Mochlus*. The first set of contrasts and p-values is for differences in slope vector length (magnitude) and the second set is for differences in slope vector orientation. Significant differences are bolded.

|  | *Acontias* | *Feylinia* | *Scelotes* | *Sepsina* | *Typhlacontias* | *Typhlosaurus* |
| --- | --- | --- | --- | --- | --- | --- |
| Contrasts in slope vector length |  |  |  |  |  |  |
| *Acontias* | 0 | * | * | * | * | * |
| *Feylinia* | 0.0182 | 0 | * | * | * | * |
| *Scelotes* | 0.0139 | 0.0042 | 0 | * | * | * |
| *Sepsina* | 0.0123 | 0.0005 | 0.0016 | 0 | * | * |
| *Typhlacontias* | 0.0254 | 0.0072 | 0.0114 | 0.0130 | 0 | * |
| *Typhlosaurus* | 0.0356 | 0.0173 | 0.0216 | 0.0231 | 0.0101 | 0 |
| p-values |  |  |  |  |  |  |
| *Acontias* | 1 | * | * | * | * | * |
| *Feylinia* | 0.6976 | 1 | * | * | * | * |
| *Scelotes* | 0.0908 | 0.8782 | 1 | * | * | * |
| *Sepsina* | 0.9304 | 0.4984 | 0.9390 | 1 | * | * |
| *Typhlacontias* | **0.0055** | 0.5800 | **0.0576** | 0.1931 | 1 | * |
| *Typhlosaurus* | **0.0004** | 0.0739 | **0.0112** | **0.0068** | 0.1820 | 1 |
| Angles between slope vectors |  |  |  |  |  |  |
| *Acontias* | 0 | * | * | * | * | * |
| *Feylinia* | 84.697 | 0 | * | * | * | * |
| *Scelotes* | 72.298 | 87.47 | 0 | * | * | * |
| *Sepsina* | 70.776 | 86.533 | 82.931 | 0 | * | * |
| *Typhlacontias* | 91.021 | 78.412 | 66.595 | 106.53 | 0 | * |
| *Typhlosaurus* | 89.879 | 100.11 | 64.928 | 95.639 | 77.663 | 0 |
| p-values |  |  |  |  |  |  |
| *Acontias* | 1 | * | * | * | * | * |
| *Feylinia* | 0.3774 | 1 | * | * | * | * |
| *Scelotes* | 0.5368 | 0.4910 | 1 | * | * | * |
| *Sepsina* | 0.7790 | 0.5513 | 0.6090 | 1 | * | * |
| *Typhlacontias* | 0.1139 | 0.7249 | 0.9133 | 0.0863 | 1 | * |
| *Typhlosaurus* | 0.1858 | 0.1929 | 0.9390 | 0.2939 | 0.7432 | 1 |

**Table S3**. Results from ANCOVA tests run with the full dataset comparing trends in cranial shape variation using genus, subfamily, number of limbs, number of digits, and substrate as the main effects and centroid size as the covariate. Interactions between each main effect and size are also reported. Significant results (p-value < 0.05) are bolded.

| Model | d.f. | SS | MS | *R*^2^ | *F* | *Z* | *P* value |
| --- | --- | --- | --- | --- | --- | --- | --- |
| Multivariate regression  **log(size)** | **1** | **0.04706** | **0.047061** | **0.10032** | **4.0141** | **2.7387** | **0.0034** |
| Residuals | 36 | 0.42206 | 0.011724 | 0.89968 |  |  |  |
| Total | 37 | 0.46912 |  |  |  |  |  |
| ANCOVA (genus)  **log(size)** | **1** | **0.04706** | **0.047061** | **0.10032** | **12.8607** | **4.7723** | **<0.001** |
| **genus** | **6** | **0.29862** | **0.049771** | **0.63657** | **13.6013** | **9.6297** | **<0.001** |
| **log(size):genus** | **5** | **0.03195** | **0.006390** | **0.06811** | **1.7462** | **8.8365** | **<0.001** |
| Residuals | 25 | 0.09148 | 0.003659 | 0.19501 |  |  |  |
| Total | 37 | 0.46912 |  |  |  |  |  |
| ANCOVA (subfamily)  **log(size)** | **1** | **0.04706** | **0.047061** | **0.10032** | **7.2218** | **3.7778** | **<0.001** |
| **subfamily** | **2** | **0.17458** | **0.087291** | **0.37215** | **13.3953** | **6.4546** | **<0.001** |
| **log(size):subfamily** | **1** | **0.03243** | **0.032430** | **0.06913** | **4.9765** | **4.8350** | **<0.001** |
| Residuals | 33 | 0.21504 | 0.006517 | 0.45840 |  |  |  |
| Total | 37 | 0.46912 |  |  |  |  |  |
| ANCOVA (limbs)  **log(size)** | **1** | **0.04706** | **0.047061** | **0.10032** | **5.4291** | **3.2689** | **<0.001** |
| **limbs** | **2** | **0.12981** | **0.064905** | **0.27671** | **7.4877** | **4.9775** | **<0.001** |
| log(size):limbs | 2 | 0.01486 | 0.007431 | 0.03168 | 0.8572 | 0.8800 | 0.2128 |
| Residuals | 32 | 0.27738 | 0.008668 | 0.59129 |  |  |  |
| Total | 37 | 0.46912 |  |  |  |  |  |
| ANCOVA (digits)  **log(size)** | **1** | **0.04706** | **0.047061** | **0.10032** | **5.0878** | **3.1574** | **<0.001** |
| **digits** | **1** | **0.09893** | **0.098926** | **0.21088** | **10.6950** | **4.5371** | **<0.001** |
| log(size):digits | 1 | 0.00864 | 0.008637 | 0.01841 | 0.9338 | 0.7814 | 0.2462 |
| Residuals | 34 | 0.31449 | 0.009250 | 0.67039 |  |  |  |
| Total | 37 | 0.46912 |  |  |  |  |  |
| ANCOVA (substrate)  **log(size)** | **1** | **0.04706** | **0.047061** | **0.10032** | **5.4692** | **3.2514** | **<0.001** |
| **substrate** | **3** | **0.10559** | **0.035195** | **0.22507** | **4.0903** | **4.1322** | **<0.001** |
| **log(size):substrate** | **3** | **0.05833** | **0.019443** | **0.12434** | **2.2596** | **3.6161** | **<0.001** |
| Residuals | 30 | 0.25814 | 0.008605 | 0.55027 |  |  |  |
| Total | 37 | 0.46912 |  |  |  |  |  |

**Table S4.** Results from ANCOVA and PGLS tests run on the reduced dataset comparing trends in cranial shape variation using genus, subfamily, number of limbs, number of digits, and substrate as the main effects, and centroid size as the covariate. Interactions between each main effect and size are also reported. Significant results are bolded.

| Model | d.f. | SS | MS | *R*^2^ | *F* | *Z* | *P* value |
| --- | --- | --- | --- | --- | --- | --- | --- |
| Multivariate regression  **log(size)** | **1** | **0.04586** | **0.45861** | **0.13606** | **4.0946** | **2.8327** | **0.0032** |
| Residuals | 26 | 0.29121 | 0.011200 | 0.86394 |  |  |  |
| Total | 27 | 0.33707 |  |  |  |  |  |
| Phylogenetic regression  **log(size)** | **1** | **0.47007** | **0.47007** | **0.18283** | **5.8172** | **3.9512** | **<0.001** |
| Residuals | 26 | 2.10098 | 0.08081 | 0.81717 |  |  |  |
| Total | 27 | 2.57105 |  |  |  |  |  |
| ANCOVA (genus)  **log(size)** | **1** | **0.04586** | **0.045861** | **0.13606** | **13.7495** | **4.9666** | **<0.001** |
| **genus** | **6** | **0.21353** | **0.035589** | **0.63350** | **10.6699** | **8.6596** | **<0.001** |
| **log(size):genus** | **3** | **0.02098** | **0.003992** | **0.06223** | **2.0962** | **7.0605** | **<0.001** |
| Residuals | 17 | 0.05670 | 0.003335 | 0.16822 |  |  |  |
| Total | 27 | 0.33707 |  |  |  |  |  |
| PGLS (genus)  **log(size)** | **1** | **0.47007** | **0.47007** | **0.18283** | **6.4775** | **4.1343** | **<0.001** |
| genus | 6 | 0.38213 | 0.06354 | 0.14828 | 0.8755 | 0.3403 | 0.3590 |
| **log(size):genus** | **3** | **0.48607** | **0.16202** | **0.18925** | **2.2327** | **3.0603** | **<0.001** |
| Residuals | 17 | 1.23368 | 0.07257 | 0.47984 |  |  |  |
| Total | 27 | 2.57105 |  |  |  |  |  |
| ANCOVA (subfamily)  **log(size)** | **1** | **0.04586** | **0.045861** | **0.13606** | **7.2164** | **3.8519** | **<0.001** |
| **subfamily** | **2** | **0.12172** | **0.060858** | **0.36110** | **9.5763** | **5.3526** | **<0.001** |
| **log(size):subfamily** | **1** | **0.02333** | **0.023328** | **0.06921** | **3.6708** | **3.6788** | **<0.001** |
| Residuals | 23 | 0.14617 | 0.006355 | 0.43364 |  |  |  |
| Total | 27 | 0.33707 |  |  |  |  |  |
| PGLS (subfamily)  **log(size)** | **1** | **0.47007** | **0.47007** | **0.18283** | **5.7289** | **3.9072** | **<0.001** |
| subfamily | 2 | 0.09195 | 0.04597 | 0.03576 | 0.5603 | -0.7293 | 0.7627 |
| log(size):subfamily | 1 | 0.12183 | 0.12183 | 0.04739 | 1.4848 | 1.5359 | 0.0675 |
| Residuals | 23 | 1.88720 | 0.08205 | 0.73402 |  |  |  |
| Total | 27 | 2.57105 |  |  |  |  |  |
| ANCOVA (limbs)  **log(size)** | **1** | **0.04586** | **0.045861** | **0.13606** | **5.8193** | **3.4540** | **<0.001** |
| **limbs** | **2** | **0.10524** | **0.052620** | **0.31222** | **6.6770** | **4.9047** | **<0.001** |
| log(size):limbs | 2 | 0.01259 | 0.006296 | 0.03736 | 0.7989 | 0.9808 | 0.1803 |
| Residuals | 22 | 0.01259 | 0.007881 | 0.51437 |  |  |  |
| Total | 27 | 0.337707 |  |  |  |  |  |
| PGLS (limbs)  **log(size)** | **1** | **0.47007** | **0.47007** | **0.18283** | **5.4413** | **3.8073** | **<0.001** |
| limbs | 2 | 0.13572 | 0.06786 | 0.05279 | 0.7855 | 0.0906 | 0.4435 |
| log(size):limbs | 2 | 0.06469 | 0.03234 | 0.02516 | 0.3744 | -1.4381 | 0.9364 |
| Residuals | 22 | 1.90057 | 0.08639 | 0.73922 |  |  |  |
| Total | 27 | 2.57105 |  |  |  |  |  |
| ANCOVA (digits)  **log(size)** | **1** | **0.04586** | **0.045861** | **0.13606** | **5.3607** | **3.3129** | **<0.001** |
| **digits** | **1** | **0.06622** | **0.066220** | **0.19646** | **7.7405** | **3.8096** | **<0.001** |
| **log(size):digits** | **1** | **0.01967** | **0.019669** | **0.05835** | **2.2992** | **2.4202** | **0.002** |
| Residuals | 24 | 0.20532 | 0.008555 | 0.60913 |  |  |  |
| Total | 27 | 0.33707 |  |  |  |  |  |
| PGLS (digits)  **log(size)** | **1** | **0.47007** | **0.47007** | **0.18283** | **5.6771** | **3.8963** | **<0.001** |
| digits | 1 | 0.08863 | 0.08863 | 0.03447 | 1.0705 | 0.8309 | 0.1987 |
| log(size):digits | 1 | 0.02512 | 0.02512 | 0.00977 | 0.3034 | -1.5254 | 0.9423 |
| Residuals | 24 | 1.98722 | 0.08280 | 0.77292 |  |  |  |
| Total | 27 | 2.57105 |  |  |  |  |  |
| ANCOVA (substrate)  **log(size)** | **1** | **0.04586** | **0.045861** | **0.13606** | **5.0927** | **3.1854** | **<0.001** |
| **substrate** | **3** | **0.07304** | **0.024347** | **0.21669** | **2.7036** | **3.2895** | **0.0010** |
| **log(size):substrate** | **3** | **0.03806** | **0.012688** | **0.11293** | **1.4090** | **2.3143** | **0.0091** |
| Residuals | 20 | 0.18011 | 0.009005 | 0.53432 |  |  |  |
| Total | 27 | 0.33707 |  |  |  |  |  |
| PGLS (substrate)  **log(size)** | **1** | **0.47007** | **0.47007** | **0.18283** | **7.1591** | **4.3315** | **<0.001** |
| **substrate** | **3** | **0.42515** | **0.14172** | **0.16536** | **2.1583** | **3.7249** | **<0.001** |
| **log(size):substrate** | **3** | **0.36261** | **0.12087** | **0.14104** | **1.8408** | **3.8650** | **<0.001** |
| Residuals | 20 | 1.31321 | 0.06566 | 0.51077 |  |  |  |
| Total | 27 | 2.57105 |  |  |  |  |  |

**Table S5**. P-values for pairwise comparisons of group means associated with ANCOVA results examining cranial shape differences across genera, excluding *Mochlus*. The first set of p-values used data prior size correction and the second set used size-corrected data. Significant differences are bolded.

|  | *Acontias* | *Feylinia* | *Scelotes* | *Sepsina* | *Typhlacontias* | *Typhlosaurus* |
| --- | --- | --- | --- | --- | --- | --- |
| *Acontias* | 1 | * | * | * | * | * |
| *Feylinia* | **0.0020** | 1 | * | * | * | * |
| *Scelotes* | **0.0002** | **0.0006** | 1 | * | * | * |
| *Sepsina* | **0.0005** | **0.0104** | 0.9343 | 1 | * | * |
| *Typhlacontias* | **0.0002** | **0.0069** | **0.0015** | **0.0089** | 1 | * |
| *Typhlosaurus* | **0.0078** | **0.0004** | **0.0001** | **0.0001** | **0.0001** | 1 |
| *Acontias* | 1 | * | * | * | * | * |
| *Feylinia* | **0.0023** | 1 | * | * | * | * |
| *Scelotes* | **0.0001** | **0.0015** | 1 | * | * | * |
| *Sepsina* | **0.0008** | **0.0122** | 0.9301 | 1 | * | * |
| *Typhlacontias* | **0.0013** | **0.0181** | **0.0026** | **0.0130** | 1 | * |
| *Typhlosaurus* | 0.1458 | **0.0156** | **0.0001** | **0.0001** | **0.0001** | 1 |

**Table S6**. P-values for pairwise comparisons of group means associated with ANCOVA results examining cranial shape differences across substrates in the full dataset. The first set of p-values used data prior size correction and the second set used size-corrected data. Significant differences are bolded.

|  | Leaf | Sand | Sandy soil | Soil |
| --- | --- | --- | --- | --- |
| Leaf | 1 | * | * | * |
| Sand | **0.0001** | 1 | * | * |
| Sandy soil | 0.0836 | **0.0082** | 1 | * |
| Soil | **0.0342** | **0.0186** | 0.4087 | 1 |
| Leaf | 1 | * | * | * |
| Sand | **0.0001** | 1 | * | * |
| Sandy soil | 0.0758 | **0.0076** | 1 | * |
| Soil | 0.1692 | 0.0833 | 0.9296 | 1 |

**Table S7**. P-values for pairwise comparisons of group means associated with ANCOVA results examining cranial shape differences across substrates in the reduced dataset. The first set of p-values used data prior size correction and the second set used size-corrected data. Significant differences are bolded.

|  | Leaf | Sand | Sandy soil | Soil |
| --- | --- | --- | --- | --- |
| Leaf | 1 | * | * | * |
| Sand | **0.0008** | 1 | * | * |
| Sandy soil | 0.1233 | **0.0175** | 1 | * |
| Soil | **0.0236** | **0.0198** | 0.2839 | 1 |
| Leaf | 1 | * | * | * |
| Sand | **0.0009** | 1 | * | * |
| Sandy soil | 0.1248 | **0.0382** | 1 | * |
| Soil | 0.0687 | 0.7460 | 0.7781 | 1 |

**Table S8**. P-values for pairwise comparisons of group means associated with PGLS results examining cranial shape differences across substrates in the reduced dataset. The first set of p-values used data prior size correction and the second set used size-corrected data. Significant differences are bolded.

|  | Leaf | Sand | Sandy soil | Soil |
| --- | --- | --- | --- | --- |
| Leaf | 1 | * | * | * |
| Sand | 0.3355 | 1 | * | * |
| Sandy soil | 0.6994 | **<0.001** | 1 | * |
| Soil | 0.8482 | **0.0438** | 0.2506 | 1 |
| Leaf | 1 | * | * | * |
| Sand | 0.4550 | 1 | * | * |
| Sandy soil | 0.7558 | **<0.001** | 1 | * |
| Soil | 0.9157 | 0.6543 | 0.9565 | 1 |

**Table S9.** Principal component scores with their standard deviations and proportion of variance explained from the PCA performed on the full dataset looking at mandibular variation.

| PC score | Standard deviation | Proportion of variance | Total variance explained |
| --- | --- | --- | --- |
| PC1 | 0.09025 | 0.57267 | 0.57267 |
| PC2 | 0.04358 | 0.13352 | 0.70619 |
| PC3 | 0.02891 | 0.05876 | 0.76495 |
| PC4 | 0.02628 | 0.04856 | 0.81351 |
| PC5 | 0.02465 | 0.04273 | 0.85624 |
| PC6 | 0.02105 | 0.03116 | 0.8874 |
| PC7 | 0.01869 | 0.02455 | 0.91195 |
| PC8 | 0.01664 | 0.01948 | 0.93142 |
| PC9 | 0.014 | 0.01378 | 0.9452 |
| PC10 | 0.01298 | 0.01185 | 0.95705 |
| PC11 | 0.0111 | 0.00867 | 0.96571 |
| PC12 | 0.00976 | 0.0067 | 0.97241 |
| PC13 | 0.00894 | 0.00562 | 0.97803 |
| PC14 | 0.00818 | 0.0047 | 0.98273 |
| PC15 | 0.00684 | 0.00329 | 0.98602 |
| PC16 | 0.00593 | 0.00247 | 0.9885 |
| PC17 | 0.0057 | 0.00228 | 0.99078 |
| PC18 | 0.00554 | 0.00216 | 0.99294 |
| PC19 | 0.00471 | 0.00156 | 0.9945 |
| PC20 | 0.00409 | 0.00118 | 0.99567 |
| PC21 | 0.00379 | 0.00101 | 0.99669 |
| PC22 | 0.00345 | 0.00084 | 0.99752 |
| PC23 | 0.00335 | 0.00079 | 0.99831 |
| PC24 | 0.00286 | 0.00057 | 0.99889 |
| PC25 | 0.00217 | 0.00033 | 0.99922 |
| PC26 | 0.00201 | 0.00028 | 0.9995 |
| PC27 | 0.00146 | 0.00015 | 0.99965 |
| PC28 | 0.00138 | 0.00013 | 0.99978 |
| PC29 | 0.00121 | 0.0001 | 0.99989 |
| PC30 | 0.001 | 0.00007 | 0.99996 |
| PC31 | 0.00069 | 0.00003 | 0.99999 |

**Table S10**. Pairwise slope comparisons from homogeneity of slopes tests comparing mandibular allometry across genera, excluding *Mochlus*. The first set of contrasts and p-values is for differences in slope vector length (magnitude) and the second set is for differences in slope vector orientation. Significant differences are bolded.

|  | *Acontias* | *Feylinia* | *Scelotes* | *Sepsina* | *Typhlacontias* | *Typhlosaurus* |
| --- | --- | --- | --- | --- | --- | --- |
| Contrasts in slope vector length |  |  |  |  |  |  |
| *Acontias* | 0 | * | * | * | * | * |
| *Feylinia* | 0.2381 | 0 | * | * | * | * |
| *Scelotes* | 0.0128 | 0.0110 | 0 | * | * | * |
| *Sepsina* | 0.0221 | 0.0017 | 0.0092 | 0 | * | * |
| *Typhlacontias* | 0.0343 | 0.0105 | 0.0215 | 0.0122 | 0 | * |
| *Typhlosaurus* | 0.0572 | 0.0.334 | 0.0443 | 0.0351 | 0.0229 | 0 |
| p-values |  |  |  |  |  |  |
| *Acontias* | 1 | * | * | * | * | * |
| *Feylinia* | 0.8742 | 1 | * | * | * | * |
| *Scelotes* | 0.3257 | 0.8794 | 1 | * | * | * |
| *Sepsina* | 0.6579 | 0.9139 | 0.7252 | 1 | * | * |
| *Typhlacontias* | **0.0122** | 0.6896 | **0.0392** | 0.3568 | 1 | * |
| *Typhlosaurus* | **0.0087** | **0.0270** | **0.0170** | **0.0127** | 0.1695 | 1 |
| Angeles between slope vectors |  |  |  |  |  |  |
| *Acontias* | 0 | * | * | * | * | * |
| *Feylinia* | 110.29 | 0 | * | * | * | * |
| *Scelotes* | 60.602 | 99.717 | 0 | * | * | * |
| *Sepsina* | 76.704 | 92.679 | 90.781 | 0 | * | * |
| *Typhlacontias* | 93.890 | 66.585 | 72.043 | 98.375 | 0 | * |
| *Typhlosaurus* | 76.178 | 84.319 | 52.994 | 68.184 | 66.836 | 0 |
| p-values |  |  |  |  |  |  |
| *Acontias* | 1 | * | * | * | * | * |
| *Feylinia* | 0.1506 | 1 | * | * | * | * |
| *Scelotes* | 0.5943 | 0.2679 | 1 | * | * | * |
| *Sepsina* | 0.5112 | 0.4274 | 0.4125 | 1 | * | * |
| *Typhlacontias* | 0.2332 | 0.8672 | 0.7356 | 0.3025 | 1 | * |
| *Typhlosaurus* | 0.5472 | 0.5870 | 0.9606 | 0.8434 | 0.8549 | 1 |

**Table S11**. Results from ANCOVA tests run with the full dataset comparing trends in mandibular shape variation using genus, subfamily, number of limbs, number of digits, and substrate as the main effects and centroid size as the covariate. Interactions between each main effect and size are also reported. Significant results are bolded.

| Model | d.f. | SS | MS | *R*^2^ | *F* | *Z* | *P* value |
| --- | --- | --- | --- | --- | --- | --- | --- |
| Multivariate regression  **log(size)** | **1** | **0.04949** | **0.049494** | **0.09405** | **3.7374** | **2.2212** | **0.0167** |
| Residuals | 36 | 0.47675 | 0.013243 | 0.90595 |  |  |  |
| Total | 37 | 0.52624 |  |  |  |  |  |
| ANCOVA (genus)  **log(size)** | **1** | **0.04949** | **0.049494** | **0.09405** | **12.3416** | **3.9005** | **<0.001** |
| **genus** | **6** | **0.34557** | **0.057594** | **0.65667** | **14.3614** | **8.0278** | **<0.001** |
| **log(size):genus** | **5** | **0.03092** | **0.006185** | **0.05876** | **1.5422** | **8.1735** | **<0.001** |
| Residuals | 25 | 0.10026 | 0.004010 | 0.19052 |  |  |  |
| Total | 37 | 0.52624 |  |  |  |  |  |
| ANCOVA (subfamily)  **log(size)** | **1** | **0.04949** | **0.049494** | **0.09405** | **6.8437** | **3.0845** | **0.0011** |
| **subfamily** | **2** | **0.20813** | **0.104067** | **0.39551** | **14.3896** | **5.8543** | **<0.001** |
| **log(size):subfamily** | **1** | **0.02996** | **0.029956** | **0.05692** | **4.1421** | **3.7820** | **<0.001** |
| Residuals | 33 | 0.23866 | 0.007232 | 0.45351 |  |  |  |
| Total | 37 | 0.52624 |  |  |  |  |  |
| ANCOVA (limbs)  **log(size)** | **1** | **0.04949** | **0.049494** | **0.09405** | **5.9272** | **2.8749** | **0.0024** |
| **limbs** | **2** | **0.19446** | **0.097230** | **0.36953** | **11.6438** | **4.8014** | **<0.001** |
| log(size):limbs | 2 | 0.01507 | 0.007537 | 0.02865 | 0.9026 | 1.3426 | 0.0954 |
| Residuals | 32 | 0.26721 | 0.008350 | 0.50777 |  |  |  |
| Total | 37 | 0.52624 |  |  |  |  |  |
| ANCOVA (digits)  **log(size)** | **1** | **0.04949** | **0.049494** | **0.09405** | **5.0838** | **2.6603** | **0.0038** |
| **digits** | **1** | **0.11999** | **0.119987** | **0.22801** | **12.3244** | **3.9431** | **<0.001** |
| **log(size):digits** | **1** | **0.02575** | **0.025748** | **0.04893** | **2.6447** | **2.4058** | **0.0060** |
| Residuals | 34 | 0.33101 | 0.009736 | 0.62901 |  |  |  |
| Total | 37 | 0.52624 |  |  |  |  |  |
| ANCOVA (substrate)  **log(size)** | **1** | **0.04949** | **0.049494** | **0.09405** | **5.3017** | **2.6800** | **0.0040** |
| **substrate** | **3** | **0.15722** | **0.052408** | **0.29876** | **5.6137** | **3.9627** | **<0.001** |
| **log(size):substrate** | **3** | **0.03946** | **0.013153** | **0.07498** | **1.4089** | **2.2553** | **0.0124** |
| Residuals | 30 | 0.28007 | 0.009336 | 0.53220 |  |  |  |
| Total | 37 | 0.52624 |  |  |  |  |  |

**Table S12.** Results from ANCOVA and PGLS tests run with the reduced dataset comparing trends in mandibular shape variation using genus, subfamily, number of limbs, number of digits, and substrate as the main effects, and centroid size as the covariate. Interactions between each main effect and size are also reported. Significant results are bolded.

| Model | d.f. | SS | MS | *R*^2^ | *F* | *Z* | *P* value |
| --- | --- | --- | --- | --- | --- | --- | --- |
| **Multivariate regression**  log(size) | **1** | **0.05095** | **0.050951** | **0.12879** | **3.8436** | **2.2329** | **0.0172** |
| Residuals | 26 | 0.34465 | 0.013256 | 0.87121 |  |  |  |
| Total | 27 | 0.39561 |  |  |  |  |  |
| Phylogenetic regression  **log(size)** | **1** | **0.54732** | **0.54732** | **0.20064** | **6.5259** | **3.8811** | **<0.001** |
| Residuals | 26 | 2.18060 | 0.08387 | 0.79936 |  |  |  |
| Total | 27 | 2.72792 |  |  |  |  |  |
| ANCOVA (genus)  **log(size)** | **1** | **0.05095** | **0.050951** | **0.12879** | **12.2608** | **3.8103** | **<0.001** |
| **genus** | **6** | **0.25386** | **0.042310** | **0.64170** | **10.1815** | **7.2448** | **<0.001** |
| **log(size):genus** | **3** | **0.02015** | **0.006717** | **0.05093** | **1.6163** | **6.5534** | **<0.001** |
| Residuals | 17 | 0.07064 | 0.004156 | 0.17857 |  |  |  |
| Total | 27 | 0.39561 |  |  |  |  |  |
| PGLS (genus)  **log(size)** | **1** | **0.54732** | **0.54732** | **0.20064** | **6.3356** | **3.7876** | **<0.001** |
| genus | 6 | 0.32355 | 0.05393 | 0.11861 | 0.6242 | -0.8985 | 0.8160 |
| **log(size):genus** | **3** | **0.38844** | **0.12948** | **0.14239** | **1.4988** | **2.3900** | **0.0056** |
| Residuals | 17 | 1.46861 | 0.08639 | 0.53836 |  |  |  |
| Total | 27 | 2.72792 |  |  |  |  |  |
| ANCOVA (subfamily)  **log(size)** | **1** | **0.05095** | **0.050951** | **0.12879** | **7.1674** | **3.1037** | **<0.001** |
| **subfamily** | **2** | **0.15065** | **0.075323** | **0.38080** | **10.5961** | **4.7456** | **<0.001** |
| **log(size):subfamily** | **1** | **0.03051** | **0.030510** | **0.07712** | **4.2919** | **3.6914** | **<0.001** |
| Residuals | 23 | 0.16350 | 0.007109 | 0.41329 |  |  |  |
| Total | 27 | 0.39561 |  |  |  |  |  |
| PGLS (subfamily)  **log(size)** | **1** | **0.54732** | **0.54732** | **0.20064** | **6.2321** | **3.7844** | **<0.001** |
| subfamily | 2 | 0.08824 | 0.04412 | 0.03235 | 0.5024 | -1.0910 | 0.8690 |
| log(size):subfamily | 1 | 0.07241 | 0.07241 | 0.02654 | 0.8244 | 0.3651 | 0.3741 |
| Residuals | 23 | 2.01995 | 0.08782 | 0.74047 |  |  |  |
| Total | 27 | 2.72792 |  |  |  |  |  |
| ANCOVA (limbs)  **log(size)** | **1** | **0.05095** | **0.050951** | **0.12879** | **6.987** | **3.0564** | **<0.001** |
| **limbs** | **2** | **0.16902** | **0.084508** | **0.42723** | **11.589** | **4.882** | **<0.001** |
| **log(size):limbs** | **2** | **0.01521** | **0.007606** | **0.03845** | **1.043** | **2.2010** | **0.0179** |
| Residuals | 22 | 0.16043 | 0.007292 | 0.40552 |  |  |  |
| Total | 27 | 0.39561 |  |  |  |  |  |
| PGLS (limbs)  **log(size)** | **1** | **0.54732** | **0.54732** | **0.20064** | **6.0901** | **3.7410** | **<0.001** |
| limbs | 2 | 0.14369 | 0.07185 | 0.05267 | 0.7994 | 0.1709 | 0.4430 |
| log(size):limbs | 2 | 0.05976 | 0.02988 | 0.02191 | 0.3325 | -1.8984 | 0.9674 |
| Residuals | 22 | 1.97714 | 0.08987 | 0.72478 |  |  |  |
| Total | 27 | 2.72792 |  |  |  |  |  |
| ANCOVA (digits)  **log(size)** | **1** | **0.05095** | **0.050951** | **0.12879** | **5.3855** | **2.7021** | **0.0041** |
| **digits** | **1** | **0.08364** | **0.083644** | **0.21143** | **8.8413** | **3.5357** | **<0.001** |
| **log(size):digits** | **1** | **0.03396** | **0.033956** | **0.08583** | **3.5892** | **2.8759** | **0.0011** |
| Residuals | 24 | 0.22705 | 0.009461 | 0.57394 |  |  |  |
| Total | 27 | 0.39561 |  |  |  |  |  |
| PGLS (digits)  **log(size)** | **1** | **0.54732** | **0.54732** | **0.20064** | **6.2542** | **3.7934** | **<0.001** |
| digits | 1 | 0.06960 | 0.06960 | 0.02551 | 0.7953 | 0.2438 | 0.4181 |
| log(size):digits | 1 | 0.01068 | 0.01068 | 0.00391 | 0.1220 | -3.2147 | 0.9988 |
| Residuals | 24 | 2.10032 | 0.08751 | 0.76994 |  |  |  |
| Total | 27 | 2.72792 |  |  |  |  |  |
| ANCOVA (substrate)  **log(size)** | **1** | **0.05095** | **0.050951** | **0.12879** | **5.5577** | **2.7309** | **0.0036** |
| **substrate** | **3** | **0.12324** | **0.041081** | **0.31153** | **4.4812** | **3.4587** | **<0.001** |
| **log(size):substrate** | **3** | **0.03806** | **0.012687** | **0.09621** | **1.3840** | **2.3853** | **0.0083** |
| Residuals | 20 | 0.18335 | 0.009167 | 0.46347 |  |  |  |
| Total | 27 | 0.39561 |  |  |  |  |  |
| PGLS (substrate)  **log(size)** | **1** | **0.54732** | **0.54732** | **0.20064** | **6.9245** | **3.9544** | **<0.001** |
| **substrate** | **3** | **0.34272** | **0.11424** | **0.12564** | **1.4453** | **2.3081** | **0.0094** |
| **log(size):substrate** | **3** | **0.25704** | **0.08568** | **0.09423** | **1.0840** | **1.8357** | **0.0274** |
| Residuals | 20 | 1.58083 | 0.07904 | 0.57950 |  |  |  |
| Total | 27 | 2.72792 |  |  |  |  |  |

**Table S13**. P-values for pairwise comparisons of group means associated with ANCOVA results examining mandibular shape differences across genera, excluding *Mochlus*. The first set of p-values used data prior size correction and the second set used size-corrected data. Significant differences are bolded.

|  | *Acontias* | *Feylinia* | *Scelotes* | *Sepsina* | *Typhlacontias* | *Typhlosaurus* |
| --- | --- | --- | --- | --- | --- | --- |
| *Acontias* | 1 | * | * | * | * | * |
| *Feylinia* | 0.1555 | 1 | * | * | * | * |
| *Scelotes* | **<0.001** | **0.0229** | 1 | * | * | * |
| *Sepsina* | **0.0053** | 0.1322 | 0.6182 | 1 | * | * |
| *Typhlacontias* | **0.0231** | 0.2960 | **0.0011** | **0.0384** | 1 | * |
| *Typhlosaurus* | **0.0350** | **0.0565** | **<0.001** | **<0.001** | **0.0209** | 1 |
| *Acontias* | 1 | * | * | * | * | * |
| *Feylinia* | 0.1299 | 1 | * | * | * | * |
| *Scelotes* | **<0.001** | **0.0050** | 1 | * | * | * |
| *Sepsina* | **0.0021** | **0.0495** | 0.5317 | 1 | * | * |
| *Typhlacontias* | 0.0653 | 0.2991 | **0.0032** | 0.0958 | 1 | * |
| *Typhlosaurus* | 0.4280 | 0.1992 | **<0.001** | **0.0014** | **0.0216** | 1 |

**Table S14**. P-values for pairwise comparisons of group means associated with ANCOVA results examining mandibular shape differences across substrates in the full dataset. The first set of p-values used data prior size correction and the second set used size-corrected data. Significant differences are bolded.

|  | Leaf | Sand | Sandy soil | Soil |
| --- | --- | --- | --- | --- |
| Leaf | 1 | * | * | * |
| Sand | **0.0001** | 1 | * | * |
| Sandy soil | 0.1455 | **0.0042** | 1 | * |
| Soil | 0.0992 | **0.0047** | 0.2949 | 1 |
| Leaf | 1 | * | * | * |
| Sand | **0.0001** | 1 | * | * |
| Sandy soil | 0.1432 | **0.0032** | 1 | * |
| Soil | 0.3043 | **0.0233** | 0.7548 | 1 |

**Table S15**. P-values for pairwise comparisons of group means associated with ANCOVA results examining mandibular shape differences across substrates in the reduced dataset. The first set of p-values used data prior size correction and the second set used size-corrected data. Significant differences are bolded.

|  | Leaf | Sand | Sandy soil | Soil |
| --- | --- | --- | --- | --- |
| Leaf | 1 | * | * | * |
| Sand | **<0.001** | 1 | * | * |
| Sandy soil | 0.1011 | **0.0208** | 1 | * |
| Soil | 0.0597 | **0.0127** | 0.3290 | 1 |
| Leaf | 1 | * | * | * |
| Sand | **<0.001** | 1 | * | * |
| Sandy soil | 0.0935 | **0.0442** | 1 | * |
| Soil | 0.1306 | 0.2781 | 0.8718 | 1 |

**Table S16**. P-values for pairwise comparisons of group means associated with PGLS results examining mandibular shape differences across substrates in the reduced dataset. The first set of p-values used data prior size correction and the second set used size-corrected data. Significant differences are bolded.

|  | Leaf | Sand | Sandy soil | Soil |
| --- | --- | --- | --- | --- |
| Leaf | 1 | * | * | * |
| Sand | 0.5165 | 1 | * | * |
| Sandy soil | 0.8405 | **0.0256** | 1 | * |
| Soil | 0.8375 | **0.0216** | 0.1949 | 1 |
| Leaf | 1 | * | * | * |
| Sand | 0.5162 | 1 | * | * |
| Sandy soil | 0.8441 | **0.0381** | 1 | * |
| Soil | 0.9877 | 0.2215 | 0.6405 | 1 |

**Table S17**. Scanning information from close-up scans of skink skulls, including information on the scan voxel size and X-ray source settings. Most specimens were scanned using a GE V|tome|xm240 CT scanner. Four specimens (*Sepsina alberti*, *Sepsina angolensis*, *Sepsina bayoni*, and *Sepsina copei*) were scanned with a Phoenix VTome|x M CT scanner.

| Species | ID | Voxel size (μm) | Voltage (kV) | Current (µa) | Power (W) |
| --- | --- | --- | --- | --- | --- |
| *Acontias aurantiacus* | CAS 195463 | 18 | 80 | 170 | 13.6 |
| *Acontias cregoi* | CAS 234196 | 16 | 80 | 170 | 13.6 |
| *Acontias gariepensis* | CAS 214522 | 22 | 80 | 170 | 13.6 |
| *Acontias gracilicauda* | CAS 147466 | 24.5 | 80 | 170 | 13.6 |
| *Acontias kgalagadi* | CAS 125809 | 13 | 80 | 170 | 13.6 |
| *Acontias lineatus* | CAS 196393 | 13 | 80 | 170 | 13.6 |
| *Acontias litoralis* | CAS 206810 | 13 | 80 | 170 | 13.6 |
| *Acontias meleagris* | CAS 173310 | 15 | 80 | 170 | 13.6 |
| *Acontias namaquensis* | MCZ 100332 | 24.5 | 80 | 170 | 13.6 |
| *Acontias orientalis* | CAS 173261 | 13 | 80 | 170 | 13.6 |
| *Acontias occidentalis* | CAS 196430 | 18 | 80 | 170 | 13.6 |
| *Acontias plumbeus* | MCZ 18358 | 19.5 | 80 | 170 | 13.6 |
| *Acontias tristis* | CAS 200021 | 13 | 80 | 170 | 13.6 |
| *Feylinia currori* | MCZ 106991 | 20 | 80 | 170 | 13.6 |
| *Feylinia elegans* | MCZ 31078 | 24 | 80 | 170 | 13.6 |
| *Mochlus sundevalli* | MCZ 28682 | 24.5 | 80 | 170 | 13.6 |
| *Scelotes arenicola* | MCZ 21292 | 16 | 80 | 170 | 13.6 |
| *Scelotes bidigittatus* | CAS 248645 | 22 | 80 | 170 | 13.6 |
| *Scelotes bipes* | CAS 224005 | 14 | 80 | 170 | 13.6 |
| *Scelotes caffer* | CAS 113507 | 14 | 80 | 170 | 13.6 |
| *Scelotes capensis* | CAS 186353 | 15 | 80 | 170 | 13.6 |
| *Scelotes fitzsimonsi* | CAS 195524 | 15 | 80 | 170 | 13.6 |
| *Scelotes gronovi* | CAS 173306 | 15 | 80 | 170 | 13.6 |
| *Scelotes kasneri* | CAS 207016 | 13 | 80 | 170 | 13.6 |
| *Scelotes limpopoensis* | CAS 234150 | 13 | 80 | 170 | 13.6 |
| *Scelotes sexlineatus* | CAS 206813 | 13 | 80 | 170 | 13.6 |
| *Sepsina alberti* | CAS 263923 | 15 | 80 | 180 | 14.4 |
| *Sepsina angolensis* | AMB 10271 | 14 | 80 | 180 | 14.4 |
| *Sepsina bayoni* | MCZ 36485 | 15 | 80 | 180 | 14.4 |
| *Sepsina copei* | AMB 10314 | 14 | 80 | 180 | 14.4 |
| *Typhlacontias brevipes* | CAS 224004 | 13 | 80 | 170 | 13.6 |
| *Typhlacontias gracilis* | MCZ 18013 | 16 | 80 | 170 | 13.6 |
| *Typhlacontias punctatissimus* | CAS 254933 | 14 | 80 | 170 | 13.6 |
| *Typhlacontias rohani* | MCZ 190458 | 16 | 80 | 170 | 13.6 |
| *Typhlosaurus braini* | CAS 214579 | 22 | 80 | 170 | 13.6 |
| *Typhlosaurus caecus* | CAS 224006 | 22 | 80 | 170 | 13.6 |
| *Typhlosaurus lomiae* | CAS 206878 | 14 | 80 | 170 | 13.6 |
| *Typhlosaurus meyeri* | CAS 201902 | 14 | 80 | 170 | 13.6 |
| *Typhlosaurus vermis* | CAS 196406 | 18 | 80 | 170 | 13.6 |

**Table S18.** Description of cranial landmarks used in study.

|  | Landmark position |
| --- | --- |
| 1 | Anteriormost point of premaxilla |
| 2 | Anteriormost point of left nasal |
| 3 | Anterior edge of nasal-maxillary suture on left maxilla |
| 4 | Center of frontal-nasal suture |
| 5 | Left lateral edge of fronto-parietal suture on frontal |
| 6 | Center of parietal notch |
| 7 | Posteriormost point of parietal, at the end of left posterior process |
| 8 | Dorsalmost point of left lacrimal foramen, placed on prefrontal |
| 9 | Posteriormost point of left maxilla, on posterior process |
| 10 | Ventralmost point of left postfrontal, on lateral process |
| 11 | Ventralmost point of left descending process of parietal |
| 12 | Posteriormost point of left postfrontal |
| 13 | Point where left epipterygoid contacts pterygoid, edge of fossa columella |
| 14 | Dorsalmost point of left quadrate |
| 15 | Posteriormost point of left quadrate, on cephalic condyle |
| 16 | Lateralmost point of mandibular condyle of left quadrate |
| 17 | Center of posteroventral edge of occipital condyle |
| 18 | Anteriormost point of left basipterygoid process of sphenoid |
| 19 | Posteriormost point of left pterygoid |
| 20 | Posterior edge of left suborbital fenestra |
| 21 | Anterolateral edge of left palatine where it borders the suborbital fenestra |

**Table S19.** Description of mandible landmarks used in study. All landmarks were placed on the right mandible.

|  | Landmark position |
| --- | --- |
| 1 | Posteriormost point of dorsal edge of retroarticular process |
| 2 | Dorsalmost point of articular |
| 3 | Posteriormost point of dentary |
| 4 | Dorsalmost point of coronoid |
| 5 | Dorsalmost point of coronoid process of dentary |
| 6 | Anteriormost point of dentary |
| 7 | Ventralmost point of mandibular symphysis of dentary |
| 8 | Posterodorsal point of mandibular symphysis of dentary |
| 9 | End of toothrow at ventral edge of final tooth or tooth loci |
| 10 | Anteroventral point of anterior process of coronoid |
| 11 | Anterodorsal point of splenial |
| 12 | Posteriormost point of posterior process of coronoid |
| 13 | Ventralmost point of retroarticular process |
